# Simple Zn(ii) complexes for the production and degradation of polyesters†

**DOI:** 10.1039/d1ra09087a

**Published:** 2022-01-07

**Authors:** Jack Stewart, Martin Fuchs, Jack Payne, Oliver Driscoll, Gabrielle Kociok-Köhn, Benjamin D. Ward, Sonja Herres-Pawlis, Matthew D. Jones

**Affiliations:** Department of Chemistry, University of Bath Claverton Down Bath BA27AY UK mj205@bath.ac.uk; Lehrstuhl für Bioanorganische Chemie, Institut für Anorganische Chemie, RWTH Aachen University Landoltweg 1 52074 Aachen Germany; Department of Chemistry, Cardiff University Park Place Cardiff CF10 3AT UK

## Abstract

Nine new complexes based on thioether appended iminophenolate (ONS) ligands have been prepared and fully characterized in solution by NMR spectroscopy. Solid-state structures were also obtained for seven complexes. In solution, all complexes were monomeric. The complexes were highly active for the polymerization of purified *rac*-lactide ([M] : [Zn] : [BnOH] = 10 000 : 1 : 30 at 180 °C) reaching TOF values up to 250 000 h^−1^. The kinetics of the polymerization have been probed by *in situ* Raman spectroscopy. The rate of reaction was dramatically reduced using technical grade *rac*-lactide with increased initiator loading. To move towards a circular economy, it is vital that catalysts are developed to facilitate chemical recycling of commodity and emerging polymeric materials. In this vein, the complexes have been assessed for their ability to break down poly(lactic acid) and poly(ethylene terephthalate). The results from both the polymerization and degradation reactions are discussed in terms of ligand functionality.

## Abstract 2

Nine new complexes based on thioether appended iminophenolate (ONS) ligands have been prepared and fully characterized in solution by NMR spectroscopy.

## Graphical abstract

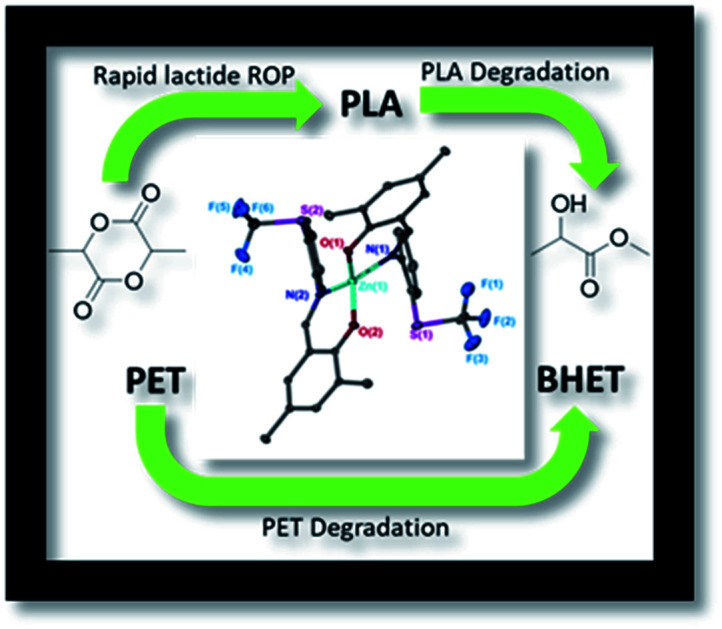

## Introduction

The widespread use of polymers has revolutionised almost every aspect of modern life. However, the proliferation of these materials has come with serious ecological implications.^1–3^ Reducing our reliance on the fossil fuel feedstocks which are necessary for producing nearly all commercial plastics is imperative due to the catastrophic environmental impact of extracting and processing these finite resources.^4^ The use of renewable feedstocks to create new polymers or to replace hydrocarbon components of existing products is therefore one of the most important scientific challenges of the 21^st^ century.^5–8^

Poly(lactic acid) (PLA) is an important bio-renewable polymer that is sourced from starch-rich materials and is amenable to enzymatic degradation or chemical recycling.^9,10^ It is established as a packaging material and has also found use in the agricultural industry and the bio-medical industry where biocompatible materials are required.^9,11–14^ PLA is typically produced through the ring-opening polymerization (ROP) of lactide initiated by a metal complex. Industrially, Sn(Oct)_2_ is used but there are toxicity issues associated with tin residues and so the current focus is to achieve industrially relevant activity with environmentally benign metal initiators. Poly(l-lactide) dominates the PLA market due to the ease of l-lactic acid biosynthesis. However, the stereoselective ROP of *rac*-lactide can improve the material properties of the polymer and thus it is often studied in academic research. A diverse range of metals has been applied to lactide ROP including Mg(ii),^15,16^ group IV,^17–24^ Fe(ii/iii),^25–32^ Al(iii),^33–49^ and In(iii).^50–52^

Initiators based on zinc(ii) have consistently shown high activity under solvent-free conditions and, in some cases, stereocontrol is observed. Coates and co-workers published a β-diiminate zinc complex that produced heterotactic PLA (*P*_r_ = 0.94) at ambient conditions.^53^ The most isoselective zinc initiator was reported by Ma and co-workers (*P*_r_ = 0.08) using an aminophenolate complex in toluene at −20 °C.^54^ However, the low temperature required for high selectivity reduced the activity (TOF = 117 h^−1^) and high stereoselectivity was not maintained under solvent-free conditions (*P*_r_ = 0.19–0.20). A dizinc bis(imino)diphenylamido initiator reported by Williams and co-workers remains the most active solution-based zinc initiator for lactide ROP (TOF = 60 000 h^−1^). Using high temperature (130–180 °C), solvent-free, industrial conditions, Jones *et al.* reported simple ethylenediamine monophenolate complexes that polymerised l-lactide with TOF values in excess of 100 000 h^−1^.^55^ Further work by the same group demonstrated the introduction of a propyl linker which increased the activity at 180 °C with l-lactide giving high conversion after just 1 minute at low initiator loading (10 000 : 1 : 33).^56^ The most active zinc initiator to date for lactide ROP was reported by Herres-Pawlis and co-workers using a bisguanidine complex which significantly outperformed Sn(Oct)_2_ and gave highly crystalline PLLA whilst also being active for technical grade *rac*-lactide.^57^ Further work using similar complexes from Pellecchia and co-workers further demonstrated their effectiveness at industrial conditions whilst also achieving lactide copolymerisation with ε-caprolactone.^58^

Most oil-based commodity plastics do not readily decompose in the environment and end up in landfill or accumulate in the oceans where plastic microparticles are devastating to marine ecosystems.^59^ Mechanical recycling can be effective in some cases but leads to material downcycling over time and this limits the number of potential cycles.^60^ Products must ultimately be repurposed and so this is a short-term solution to retaining value in the polymer economy. Conversely, chemical recycling to monomer or value-added products offers a much more long-term solution to value retention either through depolymerisation to virgin monomer or degradation to value-added products.^61^ Both offer an intrinsic economic incentive to industry either through a potentially infinite number of monomer to polymer cycles or through the upgrading of waste polymer to value-added and industrially relevant chemicals in the case of degradation.^62^ This approach has huge potential to offer a more sustainable approach to plastic usage and is crucial to attaining a circular economy for polymers.

Simple metal salts have been reported for PLA methanolysis and can give good methyl lactate yields (*Y*_Me–LA_ = 87% for FeCl_3_) but require high temperatures (130–180 °C).^63–65^ The first example of a zinc complex for PLA methanolysis was reported by Avilés *et al.* with a dizinc NHC complex capable of producing methyl lactate over 24 hours at ambient temperature.^66^ More recently, Jones and co-workers have reported several homoleptic zinc monophenolate complexes that are active for the degradation of PLA into alkyl lactates at mild conditions.^56,67–71^ An imine-monophenolate complex with a propylene diamine linker was able to achieve a high yield of methyl lactate (*Y*_Me–La_ = 89%) after just 30 minutes at 50 °C.^56^ This far exceeds the activity shown by the ethylenediamine-based analogues which required several hours to reach high conversion at comparable temperatures.^70^ The most effective propylenediamine catalyst was tested for the degradation of post-consumer PLA products into ethyl lactate.^67^ Although activity was lower than with the clear PLA cup used for the methanolysis experiments, good yields of ethyl lactate were observed and the variation in activity was attributed to additives and the ease of dissolution of each product. More recent work has demonstrated an ethylene diamine-based ligand with a catam (N–H) moiety that far exceeds the activity of the direct imine-based counterpart, achieving 85% Me–LA yields after 30 minutes at 50 °C.^70,72^ The aforementioned ligand is a building block of the tetradentate catalen system, zinc complexes of which were also active for PLA degradation to methyl lactate at moderate yields after 8 hours at 80 °C.^71^ Recent examples have also achieved PLA alcoholysis at ambient conditions and with no solvent, in some cases demonstrating full PLA conversion after 1 hour.^73,74^ Organocatalysts, such as DMAP,^75^ TBD,^76^ TMC^77^ and ionic liquids,^78–80^ have also been reported but typically require high catalyst loading and can be limited by issues relating to corrosivity, toxicity and high cost.

**Scheme 1:**
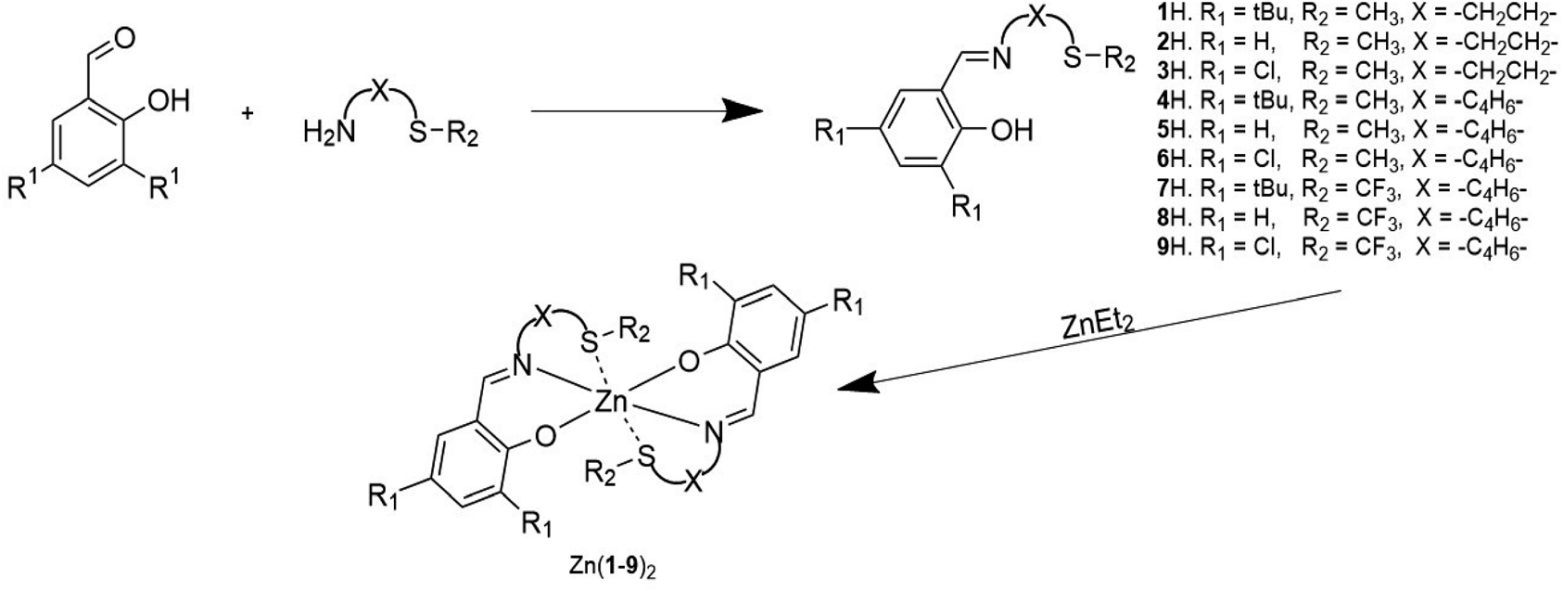
Ligands and complexes used in this study.

Herein, we report a series of nine Schiff base ligands each bearing a thioether motif where the phenolate substituents, thioether substituent and linking group have been varied systematically (Scheme 1). The homoleptic zinc(ii) complexes were applied to lactide ROP under solvent-free conditions as well as the methanolysis of commercial PLLA and the glycolysis of PET.

## Results and discussion

### Synthesis and characterisation of complexes

Ligands 1–9H were prepared *via* an imine condensation reaction between the relevant amine and salicylaldehyde derivative, with purity and structure confirmed through ^1^H NMR, ^13^C{^1^H} NMR and ESI-MS (Scheme 1 and Fig. SI1–SI18†). Complexation was performed under air-sensitive conditions in anhydrous toluene and the products were crystallised from varying mixtures of hexane and toluene. In all cases, the ^1^H NMR spectra were consistent with homoleptic complexes showing no evidence of ethyl groups remaining at the zinc centre (Fig. SI19–SI36†). This was supported by CHN analysis for all complexes.

**Fig. 1:**
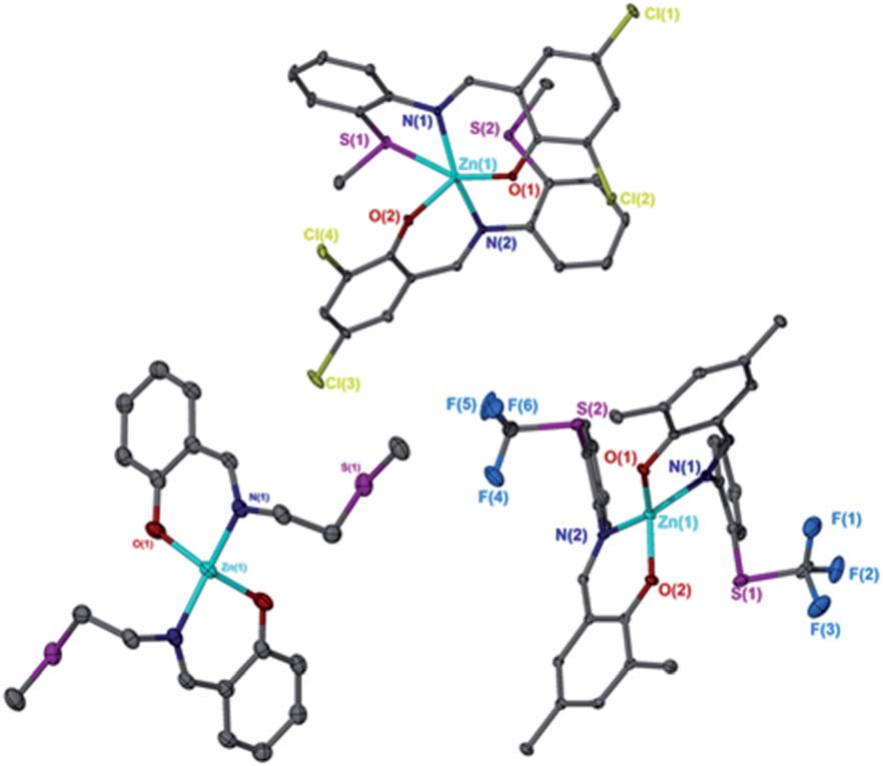
Solid-state structures of Zn(2)_2_ (left), Zn(7)_2_ (right) and Zn(6)_2_ (top). Ellipsoids shown at the 30% probability level. H atoms and ^*t*^Bu groups have been removed for clarity.

**Fig. 2:**
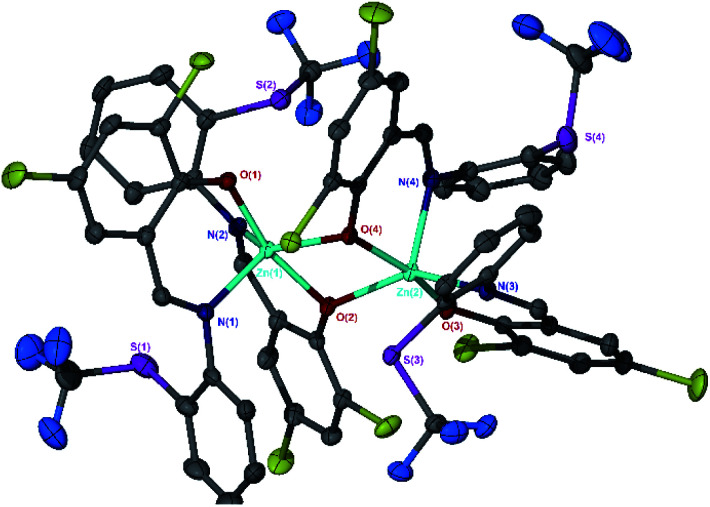
Solid-state structure of Zn(9)_2_ {Zn_2_(9)_4_}. Ellipsoids are shown at the 50% probability level. H atoms have been removed for clarity.

Solid state structures were obtained for seven complexes of which five, Zn(1, 2, 4, 5, 7)_2_, were shown to be four-coordinate with no Zn–S bonding (Table 1). Of all complexes, Zn(1)_2_ (R_1_ = ^*t*^Bu, R_2_ = CH_3_, X = –C_2_H_4_–) was closest to tetrahedral geometry 
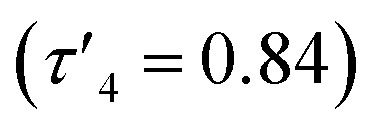
 with coordination angles relatively close to the ideal tetrahedral angle of 109.5° {95.98(10)°–121.50(10)°}. The other ethylene-bridged structure, Zn(2)_2_ (R_1_ = ^*t*^Bu, R_2_ = CH_3_, X = –C_2_H_4_–) (Fig. 1), deviates further from the tetrahedral ideal 
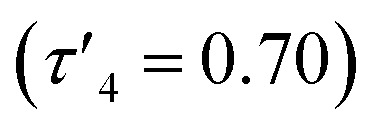
 with a large coordination angle between the two nitrogen donors and the zinc centre {N(1)–Zn–N(2) = 136.49(13)}. For both Zn(1)_2_ and Zn(2)_2_, the average interatomic Zn–S distance is large: 4.4 Å and 4.5 Å respectively. These are greater than the sum of the van der Waals radii (Zn: 2.39 Å, S: 1.89)^81^ and thus it is reasonable to assume that there is no bonding interaction. The geometry of Zn(4)_2_ deviates further from tetrahedral 
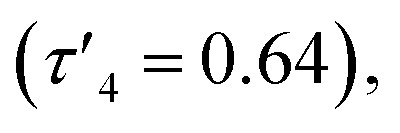
 probably due to the rigid phenylene linker constraining chelation. This is also true for Zn(5)_2_ where two crystallographic structures were present in the sample, both with a relatively low geometry index {Zn(5)_2_A, 
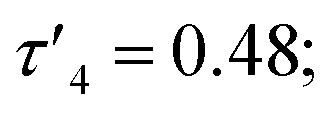
 Zn(5)_2_B, 
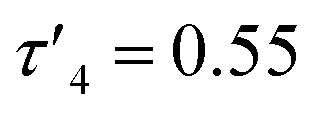
}. The average distance between zinc and sulphur atoms is considerably shorter than with the ethylene-bridged analogues, with values below the sum of van der Waals radii {Zn(4)_2_, 3.1 Å; Zn(5)_2_A, 3.1 Å; Zn(5)_2_B, 3.2 Å} suggesting that there could be a degree of bonding interaction. Again, this is likely a consequence of the more rigid structure forcing the sulphur to approach the metal centre more closely. Modification of the thioether substituent to CF_3_ {Zn(7)_2_: R_1_ = ^*t*^Bu, R_2_ = CF_3_, X = –C_6_H_4_–} (Fig. 1) caused the geometry to become more tetrahedral 
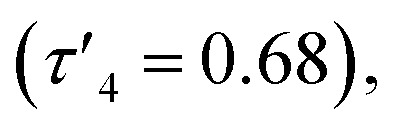
 and caused the average Zn–S distance to increase to 3.4 Å. This reflects a combination of the modification in electron density at the potential sulphur donor atom and the steric difference between the substituents. The solid-state structure of Zn(6)_2_ indicates a five-coordinate geometry with one sulphur atom formally bonded to zinc (Fig. 1). The geometry index for a five-coordinate complex, *τ*_5_, was calculated as 0.02, indicating an almost perfectly square pyramidal geometry. Considering the similarity of the Zn–S bond lengths {Zn(1)–S(1) = 2.8032(18) Å, Zn(1)–S(2) = 2.9 Å}, an octahedral geometry is a more feasible description of the structure. This is supported by Bader's quantum theory of atoms in molecules (QTAIM) analysis, in which a bond critical point (BCP) was located between Zn(1) and S(2) with *ρ* = 0.145 e Å^−3^ (*cf.* BCP from Zn(1) to S(1) has *ρ* = 0.185 e Å^−3^). Assuming an octahedral geometry, the ligands wrap in a *mer*–*mer* orientation with the thioether and phenoxy donors of the respective ligands adopting a pseudo axial position {O(1)–Zn–S(1) = 157.45(3)°, O(2)–Zn–S(2) = 153.02°}. The complex based on ligand 9H gave a dimeric structure of the form Zn_2_(9)_4_ with two five coordinate zinc centres (Fig. 2). Zn(1) tended slightly towards trigonal bipyramidal (*τ*_5_ = 0.54) and Zn(2) was much closer to square pyramidal (*τ*_5_ = 0.33). There are a range of Zn–S interatomic distances with the closest (Zn–S = 3.4 Å) situated *trans* to the apical Zn(2)–N(4) bond. As with Zn(6)_2_, some degree of Zn–S interaction could be influencing the geometry at Zn(2). DOSY NMR was used to assess whether the dimeric structure is maintained in solution through comparison with Zn(4)_2_ and Zn(7)_2_. One species was observed for each complex. Comparison between the diffusion constant of the complex and the solvent (Table SI2†) was used to calculate the hydrodynamic radii of the complexes. All were sufficiently similar to indicate a consistent structure {*r*Zn(4)_2_ = 7.25 Å, *r*Zn(7)_2_ = 6.30 Å, *r*Zn(9)_2_ = 7.13 Å}. Based on an approximate measurement of the complex diameter from the solid-state structures, it is likely that the monomeric homoleptic complex is present exclusively in solution.

**Table 1:** Selected bond lengths (Å) and angles (°) for Zn(1, 2, 4, 5, 7)_2_

	Zn(1)_2_	Zn(2)_2_	Zn(4)_2_	Zn(5)_2_A	Zn(5)_2_B	Zn(7)_2_
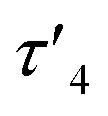 a	0.84	0.70	0.64	0.48	0.55	0.68
Zn–S^b^	4.4	4.5	3.1	3.1	3.2	3.4
Zn–O(1)	1.896(2)	1.944(2)	1.9304(11)	1.9555(15)	1.9251(14)	1.9188(17)
Zn–O(2)	1.921(2)	1.944(2)	1.9304(11)	1.9478(15)	1.9251(14)	1.9199(17)
Zn–N(1)	1.989(3)	1.993(2)	2.0539(14)	2.0755(17)	2.0454(16)	2.019(2)
Zn–N(2)	2.003(3)	1.933(2)	2.0539(14)	2.0701(17)	2.0454(16)	2.028(2)
O(1)–Zn–O(2)	121.50(10)	115.34(13)	118.05(7)	97.62(6)	97.03(9)	105.10(7)
O(1)–Zn–N(1)	97.49(10)	95.89(9)	93.10(5)	91.34(7)	94.70(6)	94.46(8)
O(1)–Zn–N(2)	115.15(10)	107.08(9)	106.63(5)	149.11(7)	140.88(7)	133.98(8)
O(2)–Zn–N(1)	119.01(10)	107.08(9)	106.63(5)	139.28(7)	140.88(7)	127.79(8)
O(2)–Zn–N(2)	95.98(10)	95.89(9)	93.10(5)	91.84(7)	94.70(6)	94.13(8)
N(1)–Zn–N(2)	108.15(10)	136.49(13)	141.39(8)	100.47(7)	99.30(9)	106.11(9)

a Calculated from the two largest coordination angles.

b Average of Zn–S interatomic distance.

### Lactide polymerisation

Complexes Zn(1–9)_2_ were all active for solvent-free, *rac*-lactide polymerisation at relatively high initiator loading ([LA] : [I] : [BnOH] = 300 : 1 : 1) (Table 2). The complexes with ethylene linkers, Zn(1–3)_2_, were less active than their more rigid, phenylene bridged counterparts at this loading. The introduction of an electron withdrawing CF_3_ group, Zn(7–9)_2_, had minimal effect on the activity giving broadly similar results to the CH_3_ substituted complexes, Zn(4–6)_2_. Interestingly, the complexes with chloro-phenolate substitution were the least active in each series, despite the electron withdrawing properties which might be expected to increase the Lewis acidity at the zinc centre and hence the activity. Dispersities ranged from 1.21–1.88 with the unsubstituted complexes, Zn(2, 5, 8)_2_, giving the highest dispersities in their respective series, as may be expected from the lack of steric hindrance. The PLA produced by Zn(1–9)_2_ was essentially atactic (*P*_r_ = 0.51–0.60) and this is in keeping with other zinc monophenolate complexes under these conditions.^56,69,71^ When the lactide to initiator ratio was increased, ([LA] : [I] : [BnOH] = 3000 : 1 : 10) the activity differences between the complexes became more apparent (Table 3). Ethylene bridge complexes, Zn(1–3)_2_, took much longer to reach high conversion (*t* = 30–60 min) than the phenylene bridge equivalents. The chlorinated analogues were the least active in each series whilst also showing a slight increase in heterotacticity compared to the other initiators. This reflects an alteration of the coordination sphere, facilitated by the chloride substituents, that increases steric crowding of the zinc centre. The octahedral structure of Zn(6)_2_ is evidence of this effect, which could also apply to Zn(3)_2_. There is no significant difference in activity between the –CH_3_ and –CF_3_ substituted complexes, except in the case of Zn(9)_2_, where the CF_3_ group appears to partially negate the drop in activity that is associated with the chloride substituents. Phenylene bridged complexes, where R_1_ = H or ^*t*^Bu [Zn(4, 5, 7, 8)], were clearly the most active initiators converting sufficient lactide after less than 5 minutes to stop stirring. Reasonable molecular weight control was maintained throughout and there was a narrowing of dispersity, (*Đ* = 1.08–1.48) that is often associated with increasing the monomer to initiator ratio. The differences in activity between the complexes show some correlation with the measured Zn–S interatomic distance (Table 1) in a volcano-style relationship (Fig. SI47†). Ethylene bridge complexes, Zn(1, 2)_2_, where the Zn–S distance is large (4.4–4.5 Å), and there is presumably no interaction, are comparatively slow. Conversely, the direct phenylene-bridged analogues {Zn(4, 5, 7)_2_} have a smaller average Zn–S distance (3.1–3.4 Å) and are the most active for lactide polymerisation. Zn(9)_2_ has an average Zn–S distance of 3.9 Å, intermediate to the two aforementioned groups of complexes, and this is reflected in the activity. The zinc centre in Zn(6)_2_ is considerably closer to the two sulphur atoms (Zn–S = 2.8–2.9 Å). The activity is similar to the ethylene-bridged complexes. This trend can be rationalised by considering the coordination of lactide to the saturated zinc centre. When the Zn–S distance is small, the complexes tend to distort further from tetrahedral geometry (*e.g.* Zn(1)_2_: 
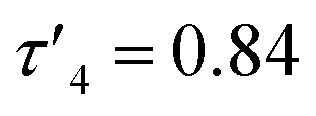
, TOF = 2080 h^−1^; Zn(1)_2_: 
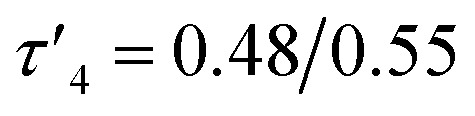
, TOF = 124 200 h^−1^). The displacement of weakly bound sulphur by lactide is easier than coordination to an undistorted tetrahedral centre. This explains the increase in activity up to a Zn–S distance of around 3 Å, after which the sulphur becomes more difficult to displace and the activity drops sharply.

**Table 2:** Solvent-free polymerisation of *rac*-lactide at 130 °C using Zn(1–9)_2_, [LA]/[Zn]/[BnOH] = 300 : 1 : 1^a^

Init.	Time/min	Conv.^b^%	*P*_r_ c	*M*_n_ e	*M*_n_ calc.^d^	*Đ* e	TOF/h^−1^^f^
Zn(1)_2_	3	59	0.55	25 600	25 600	1.32	3550
Zn(2)_2_	7	73	0.55	24 450	31 650	1.34	1900
Zn(3)_2_	25	60	0.60	22 400	26 050	1.26	450
Zn(4)_2_	2	81	0.57	22 500	35 100	1.59	7300
Zn(5)_2_	<1	56	0.57	21 050	24 300	1.88	10 100
Zn(6)_2_	3	61	0.57	31 350	26 500	1.21	3650
Zn(7)_2_	1	72	0.59	24 950	31 250	1.52	12 950
Zn(8)_2_	2	72	0.51	25 500	31 250	1.66	6500
Zn(9)_2_	10	46	0.58	17 950	26 600	1.27	850

a Conditions: *rac*-LA (1 g), [LA]/[Zn]/[BnOH] = 300 : 1 : 1, solvent free.

b Determined by ^1^H NMR spectroscopy.

c Probability of racemic enchainment, determined by ^1^H{^1^H} NMR spectroscopy.

d Theoretical molecular weight calculated from conversion (rounded to the nearest 50): {(conversion × 3 × *M*_n_ [LA]) + *M*_n_ [BnOH]}.

e Determined from GPC (in tetrahydrofuran) referenced against polystyrene standards × 0.58.

f TOF = [LA]_*t*_/([Zn] × *t*).

**Table 3:** Solvent-free polymerisation of *rac*-lactide at 130 °C using Zn(1–9)_2_, [LA]/[Zn]/[BnOH] = 3000 : 1 : 10^a^

Init.	Time/min	Conv.^b^%	*P*_m_ c	*M*_n_ e	*M*_n_ calc.^d^	*Đ* e	TOF/h^−1^^f^
Zn(1)_2_	45	52	0.56	20 100	22 600	1.17	2080
Zn(2)_2_	30	73	0.58	11 150	31 650	1.14	4380
Zn(3)_2_	60	40	0.62	4750	5850	1.08	1200
Zn(4)_2_	4	71	0.60	23 750	30 800	1.23	31 950
Zn(5)_2_	1	69	0.57	25 150	29 950	1.28	124 200
Zn(6)_2_	40	53	0.61	22 250	23 050	1.16	2385
Zn(7)_2_	2	73	0.57	16 950	31 650	1.48	65 700
Zn(8)_2_	4	70	0.56	27 800	30 400	1.38	31 500
Zn(9)_2_	12	64	0.60	20 200	27 800	1.36	9600

a Conditions: *rac*-LA (1.5 g), [LA]/[Zn]/[BnOH] = 3000 : 1 : 10, solvent free.

b Determined by ^1^H NMR spectroscopy.

c Probability of racemic enchainment, determined by ^1^H{^1^H} NMR spectroscopy.

d Theoretical molecular weight calculated from conversion (rounded to the nearest 50): {(conversion × 3 × *M*_n_ [LA]) + *M*_n_ [BnOH]}.

e Determined from GPC (in tetrahydrofuran) referenced against polystyrene standards × 0.58.

f TOF = [LA]_*t*_/([Zn] × *t*).

The polymer produced by Zn(3)_2_ at this loading was suitable for MALDI-ToF analysis (Fig. SI43†). The repeat unit of the main series is 144 g mol^−1^, and the spectrum is centred around 4457.9 g mol^−1^; similar to that measured by GPC and to the theoretical molecular weight (*M*_n GPC_ = 4750 g mol^−1^, *M*_n calc._ = 5850 g mol^−1^). At low molecular weight there is evidence of ionisation by incidental potassium and a small amount of transesterified PLA.

The most active initiators, Zn(4, 5, 7, 8, 9)_2_, were tested for *rac*-lactide polymerisation at very low catalyst loading ([LA] : [I] : [BnOH] = 10 000 : 1 : 30); similar to the concentrations employed in an industrial setting with Sn(Oct)_2_ (Table 4).^82^ Under these conditions, Zn(5)_2_ (R_1_ = H, R_2_ = CH_3_, X = C_6_H_4_) and Zn(7)_2_ (R_1_ = ^*t*^Bu, R_2_ = CF_3_, X = C_6_H_4_) were clearly the most active initiators achieving 68% and 51% conversion in 3 and 9 minutes respectively. These were selected for further kinetic analysis. With the exception of Zn(5)_2_, conversion was limited to around 50%, suggesting some initiator decomposition is taking place. The molecular weight control was maintained at this loading and the dispersities remained consistent (*Đ* = 1.20–1.44). To further probe the effectiveness of these complexes at industrial conditions, the most active complexes, Zn(4, 5, 7, 8, 9)_2_, were tested at 180 °C (Table 5). All the initiators tested were sufficiently robust to show activity at this temperature and were consistently more active than at 130 °C (*t* = 2–12 min, conversion = 56–83%), due both to the increase in temperature and the decreased viscosity allowing for better mixing at higher conversions. As might be expected at elevated temperatures, the dispersities were broader (*Đ* = 1.45–1.68) although reasonable molecular weight control was maintained. Zn(5)_2_ and Zn(7)_2_ were also tested for the polymerisation of l-lactide, the most commonly used industrial monomer. There was a small reduction in activity with l-lactide for both initiators, although the activity was still high. The reaction was very well controlled with comparatively narrow dispersities (Zn(5)_2_, *Đ* = 1.16; Zn(7)_2_, *Đ* = 1.15) and excellent molecular weight control (Zn(5)_2_, *M*_n_ = 32 050 g mol^−1^, *M*_n (theo.)_ = 35 750 g mol^−1^; Zn(7)_2_, *M*_n_ = 28 050 g mol^−1^, *M*_n (theo.)_ = 27 050 g mol^−1^). DSC analysis showed that the melting point of PLLA produced by Zn(5)_2_ and Zn(7)_2_ was 162 °C and 167 °C respectively (Fig. SI45 and SI46†). This suggests that there is limited epimerisation and the presence of a single methine peak in the ^1^H{^1^H} NMR spectrum supports this conclusion. Despite the yellow colouring of the initiators, white PLA was produced at the highest ratios employed and this is desirable from a commercial standpoint (Fig. SI44†). The performance of Zn(5)_2_ and Zn(7)_2_ with l-lactide provides a direct comparison with analogous zinc ONN complexes. Ethylene-bridged complexes reported by Jones and co-workers gave comparable results at similar conditions (180 °C, [LA] : [I] : [BnOH] = 10 000 : 1 : 33) taking 3 minutes to achieve a higher conversion of 94%.^55^

**Table 4:** Solvent-free polymerisation of *rac*-lactide at 130 °C using Zn(4, 5, 7, 8, 9)_2_. [LA]/[Zn]/[BnOH] = 10 000 : 1 : 30^a^

Init.	Time/min	Conv.^b^%	*P*_m_ c	*M*_n_ e	*M*_n_ calc.^d^	*Đ* e	TOF/h^−1^^f^
Zn(4)_2_	30	51	0.58	23 600	24 600	1.23	10 200
Zn(5)_2_	3	68	0.57	29 750	32 850	1.20	136 000
Zn(7)_2_	9	51	0.58	21 100	24 450	1.28	34 000
Zn(8)_2_	45	54	0.58	14 400	26 100	1.44	7200
Zn(9)_2_	30	55	0.61	17 200	26 600	1.31	11 000

a Conditions: *rac*-LA (3 g), [LA]/[Zn]/[BnOH] = 10 000 : 1 : 30, solvent free.

b Determined by ^1^H NMR spectroscopy.

c Probability of racemic enchainment, determined by ^1^H{^1^H} NMR spectroscopy.

d Theoretical molecular weight calculated from conversion (rounded to the nearest 50): {(conversion × 3 × *M*_n_ [LA]) + *M*_n_ [BnOH]}.

e Determined from GPC (in tetrahydrofuran) referenced against polystyrene standards × 0.58.

f TOF = [LA]_*t*_/([Zn] × *t*).

**Table 5:** Solvent-free polymerisation of *rac*-lactide at 180 °C using Zn(4, 5, 7, 8, 9)_2_. [LA]/[Zn]/[BnOH] = 10 000 : 1 : 30^a^

Init.	Time/min	Conv.^b^%	*P*_m_ c	*M*_n_ e	*M*_n_ calc.^d^	*Đ* e	TOF/h^−1^^h^
Zn(4)_2_	3	74	0.57	28 100	35 750	1.62	148 000
Zn(5)_2_	2	83	0.57	31 350	40 050	1.53	249 000
Zn(5)_2_^f^	4	74	0.00^g^	32 050	35 750	1.16	111 000
Zn(7)_2_	6	67	0.58	26 850	32 350	1.45	67 000
Zn(7)_2_^f^	6	56	0.00^g^	28 050	27 050	1.15	56 000
Zn(8)_2_	3	69	0.58	27 150	33 300	1.60	138 000
Zn(9)_2_	12	65	0.62	24 400	31 400	1.68	32 500

a Conditions: *rac*-LA (3 g), [LA]/[Zn]/[BnOH] = 10 000 : 1 : 30, solvent free.

b Determined by ^1^H NMR spectroscopy.

c Probability of racemic enchainment, determined by ^1^H{^1^H} NMR spectroscopy.

d Theoretical molecular weight calculated from conversion (rounded to the nearest 50): {(conversion × 3 × *M*_n_ [LA]) + *M*_n_ [BnOH]}.

e Determined from GPC (in tetrahydrofuran) referenced against polystyrene standards × 0.58.

f l-Lactide used.

g One peak present in ^1^H{^1^H} NMR spectrum.

h TOF = [LA]_*t*_/([Zn] × *t*).

In order to probe the polymerisation mechanism and the role of the co-initiator, a stoichiometric reaction was carried out. A 1 : 1 solution of *rac*-lactide and Zn(7)_2_ in CDCl_3_ was heated to 50 °C for 90 min and monitored by NMR spectroscopy (Fig. SI48†). Resonances similar to those of opened lactide increased significantly upon addition of BnOH to 60% in ten minutes at room temperature and 94% conversion after a further 90 min at 50 °C. This shows that a co-initiator is required for high activity but that a degree of activity might be observed in its absence. This could be explained through initiation by impurities in *rac*-lactide or through a coordination–insertion mechanism facilitated by a dissociated ligand as observed by McKeown *et al.* with zinc ONN complexes.^55^ Some evidence of ligand dissociation was observed in the methyl region of the ^1^H NMR spectrum where two minor resonances corresponding to *t*-butyl groups steadily increased throughout the reaction (Fig. SI49†). Furthermore, a new signal was present in the ^19^F NMR spectrum at the end of the reaction (Fig. SI50†). DOSY NMR analysis of the final reaction mixture showed that the ring-opened lactide had a distinctly different diffusion constant to the metal complex (Fig. SI51†). This suggests an activated monomer mechanism wherein the components of the reaction are never fully bonded to the metal and the co-initiator is exogenous. Therefore, the best explanation for the conversion observed prior to BnOH addition is through initiation by impurities in the monomer. The observation of additional resonances in the ^1^H and ^19^F NMR spectra is evidence of complex decomposition and could account for the limited conversion achieved by these systems.

### Raman kinetics

To further probe the effectiveness of these complexes at industrial conditions, the kinetics were investigated using *in situ* Raman spectroscopy. In these experiments Zn(5)_2_ and Zn(7)_2_ were taken forward, initially testing at 150 °C at a *rac*-LA : Zn ratio of 2500 : 1 using technical grade lactide, as a direct comparison to previous systems reported by Herres-Pawlis.^57,83,84^ Results were disappointing with low conversion (*ca.* 15–40%) being achieved after 3 h. This may indicate that the complexes herein are less stable to impurities in the monomer. More promising results were achieved using recrystallised l-LA and 4-methylbenzyl alcohol in a 3000 : 1 : 10 ([LA] : [Zn] : [4-MeBnOH]) ratio (Fig. SI53†). Zn(5)_2_ proved more active with a conversion of 48% (*M*_n_ = 17 900 g mol^−1^, *Đ* = 1.06 after 3 h), and a *k*_app_ = 1.3 × 10^−3^ s^−1^. The corresponding value for Zn(7)_2_ was *k*_app_ = 1.7 × 10^−4^ s^−1^. The process was modified to mimic that of the smaller scale testing with Zn(7)_2_ and benzyl alcohol as a co-initiator (Fig. SI55†). A marked increase in activity was observed (*k*_app_ = 2.2 × 10^−3^ s^−1^) and a conversion of 75% was achieved after 45 min (*M*_n_ = 31 200 g mol^−1^, *Đ* = 1.22). Zn(7)_2_ was further tested at a ratio of 10 000 : 1 : 100 ([LA] : [Zn] : BnOH) achieving 34% after 6 hours (*k*_app_ = 9.95 × 10^−5^ s^−1^) (Fig. SI57†).

### Polyester degradation

Zn(1–9)_2_ were investigated for PLA methanolysis at 80 °C in THF. The product, Me–LA, can be converted to lactide for the production of virgin PLA and is also an emerging green solvent.^62^ Furthermore, Me–LA can replace lactic acid in many transformations so has potential as a platform chemical.^85^ Waste polymer from a commercial source (0.25 g, PLLA cup, *M*_n_ = 45 510 g mol^−1^) and catalyst (20 mg, 8 wt%) were added in a glovebox and subsequently dissolved in THF. When the polymer was fully solubilised, methanol was added to initiate the degradation reaction.

This reaction has been shown to proceed *via* a two-step mechanism with PLA initially broken down into oligomers before being converted to the product.^70^ Analysis of the methine region (*ca. δ* = 4.2–5.2 ppm) of the ^1^H NMR spectrum gives three key parameters: internal methine conversion (*X*_int_), Me–LA yield (*Y*_Me–LA_) and the selectivity to Me–LA (*S*_Me–LA_).

All complexes were active for the methanolysis of PLA (Table 6). Of the ethylene bridge complexes, Zn(1–3)_2_, the highest activity was recorded for Zn(2)_2_ (*X*_int_ = 87%) and this complex produced the most Me–LA of all the catalysts that were tested (*S*_Me–LA_ = 72%, *Y*_Me–LA_ = 63%). This contrasts with the polymerisation studies where Zn(2)_2_ significantly underperformed most of the phenylene-bridged analogues. This can be rationalised by considering the equilibrium between oligomer and methyl lactate where the backward reaction (*k*_−2_) will be slower with a less effective polymerisation catalyst thus driving the reaction towards the product. The most active catalysts were Zn(4)_2_ (R_1_ = ^*t*^Bu, R_2_ = CH_3_, X = C_6_H_4_) and Zn(4)_2_ (R_1_ = H, R_2_ = CH_3_, X = C_6_H_4_) which gave internal methine conversions of 94% and 96% respectively. The latter also gave high selectivity and yield of Me–LA (*Y*_Me–LA_ = 59%, *S*_Me–LA_ = 62%). As observed during polymerisation, Zn(6)_2_ was the slowest in this series (R_1_ = Cl, R_2_ = CH_3_, X = C_6_H_4_) presumably resulting from steric crowding from the pseudo-octahedral structure. Interestingly, the introduction of a CF_3_ group at the R_2_ position resulted in a sharp decline in activity, particularly for the non-halogenated complexes Zn(7–8)_2_, both of which converted 39% of internal methine after 8 hours with correspondingly low Me–LA yields. However, Zn(9)_2_ (R_1_ = Cl, R_2_ = CF_3_, X = C_6_H_4_) achieved good activity and selectivity (*X*_int_ = 81%, *S*_Me–LA_ = 50%, *Y*_Me–LA_ = 41%) under the same conditions.

**Table 6:** Degradation of PLLA cup to Me–LA using Zn(1–9)_2_ at 80 °C^a^

Cat.	Time/h	Cat. loading/wt%	*Y*_Me–LA/%_	*S*_Me–LA/%_	*X*_int/%_
Zn(1)_2_	8	8	24	33	72
Zn(2)_2_	8	8	63	72	87
Zn(3)_2_	8	8	13	20	65
Zn(4)_2_	8	8	47	50	94
Zn(5)_2_	8	8	59	62	96
Zn(6)_2_	8	8	32	41	78
Zn(7)_2_	8	8	6	16	39
Zn(8)_2_	8	8	7	17	39
Zn(9)_2_	8	8	41	50	81

a Reaction conditions: 0.25 g of PLLA cup (*M*_n_ = 45 510 g mol^−1^), *V*_THF_ : *V*_MeOH_ = 4 : 1, *n*_MeOH_ : *n*_ester_ = 7 : 1, 8 wt% cat. loading (1.3–2.1 mol% relative to ester linkages). *Y*_Me–LA_, *S*_Me–LA_ and *X*_int_ determined by ^1^H NMR upon solvent removal.

The most active catalysts [Zn(2, 4, 5, 9)_2_ were tested at 50 °C for 18 hours (Table 7). There was a reduction in activity and selectivity compared with the 80 °C reactions and this is consistent with literature examples.^69,86^ Performance particularly decreased for Zn(4)_2_ (*X*_int_ = 57%, *S*_Me–LA_ = 21%, *Y*_Me–LA_ = 12%) and Zn(5)_2_ (*X*_int_ = 56%, *S*_Me–LA_ = 25%, *Y*_Me–LA_ = 14%). There was an indication that Zn(4–5)_2_ are sensitive to residual moisture in the THF and this could lead to deactivation over extended reaction times. Due to the lack of labile alkyl groups around the metal, it is likely that this hydrolytic sensitivity comes from the active species or a reaction intermediate. Zn(2)_2_ remained the most selective catalyst and gave reasonable conversion of internal methine (*X*_int_ = 77%).

**Table 7:** Degradation of PLLA cup to Me–LA using Zn(2, 4, 5, 9)_2_ at 50 °C^a^

Cat.	Time/h	Cat. loading/wt%	*Y*_Me–LA/%_	*S*_Me–LA/%_	*X*_int/%_
Zn(2)_2_	18	8	31	41	77
Zn(4)_2_	18	8	12	21	57
Zn(5)_2_	18	8	14	25	56
Zn(9)_2_	18	8	16	26	62

a Reaction conditions: 0.25 g of PLLA cup (*M*_n_ = 45 510 g mol^−1^), *V*_THF_ : *V*_MeOH_ = 4 : 1, *n*_MeOH_ : *n*_ester_ = 7 : 1, 8 wt% cat. loading (1.3–2.1 mol% relative to ester linkages). *Y*_Me–LA_, *S*_Me–LA_ and *X*_int_ determined by ^1^H NMR upon solvent removal.

Kinetic analysis of Zn(2, 4, 5, 9)_2_ was attempted through taking aliquots every hour for 4–6 hours with a final point measured after 8 hours. Based on previous literature, pseudo first-order kinetics of PLA consumption were assumed.^70^ Zn(4)_2_ and Zn(5)_2_ were not amenable to sampling and the reactivity was quenched.

To expand the scope of polyester degradation, the most active PLA degradation catalysts were applied to the glycolysis of PET to bis(2-hydroxyethyl) terephthalate (BHET); a useful product that can be polymerised to make virgin PET or can be used to make unsaturated polymer resins.^87,88^ PET is one of the most prevalent commodity plastics accounting for 9% of global plastic demand in 2015.^89^ Although widely mechanically recycled, it is still a major contributor to plastic pollution and is not degradable in the environment.^90^ Zn[2,4,5,9]_2_ were all active for the glycolysis of PET, with full dissolution observed after 1.5–4 hours for a standard carbonated drinks bottle (Table 8). Full degradation of PET was assumed at this point and BHET was isolated through recrystallisation from water. Isolated yields between 42% and 55% were observed, and this is consistent with previous literature examples using zinc catalysts.^71,72^ The most active catalyst {Zn(2)_2_: *t* = 1.5 h, *Y*_BHET_ = 0.16 g (48%) was tested with thin-film PET; a proxy for manufacturing waste. As expected, the reaction time was significantly reduced (*t* = 0.75 h) and a percentage yield of 51% was attained, again in keeping with previous results by Payne *et al.*^71,72^

**Table 8:** Degradation of PET into BHET using Zn(1–9)_2_ at 180 °C^a^

Cat.	Time/hours	*T*/°C	Cat. loading/wt%	*Y*_BHET_ (g/%)
Zn(2)_2_	1.5	180	8	0.16 (48%)
Zn(2)_2_^b^	0.75	180	8	0.17 (51%)
Zn(4)_2_	4	180	8	0.18 (55%)
Zn(5)_2_	4	180	8	0.14 (42%)
Zn(9)_2_	3.5	180	8	0.17 (51%)

a Reaction conditions: 0.25 g of carbonated drinks bottle (*M*_n_ ∼ 40 000 g mol^−1^), 27.5 equivalents of EG (relative to ester linkages), 8 wt% cat. loading (0.02 g, 1.9–3.4 mol% relative to ester linkages).

b 0.25 g PET thin film.

In the reaction with Zn(2)_2_, colourless BHET was obtained after recrystallisation and this is important when considering the quality of PET if it were repolymerized (Fig. SI62†).^91^

## Conclusions

A series of nine ONS zinc complexes were prepared and characterized through NMR spectroscopy and single-crystal X-ray diffraction. The rapid polymerization of *rac*-lactide was demonstrated under industrially relevant, solvent-free conditions. Up to 83% conversion was achieved in 2 minutes at 180 °C with TOF values up to 250 000 h^−1^. Crystalline PLLA was also produced rapidly with minimal epimerization. Good control of molecular weight and dispersity (*Đ* = 1.08–1.88) was maintained throughout. Raman kinetic experiments showed that the activity is significantly reduced when using technical grade *rac*-lactide even at relatively high catalyst loading. For the optimised preparation with Zn(7)_2_, an apparent rate constant *k*_app_ = 2.23 × 10^−3^ s^−1^ was measured. All complexes were active for the degradation of PLA to methyl lactate under mild conditions. Two waste sources of PET were also degraded to colourless BHET with full PET consumption observed after 0.75–4 hours (Scheme 1).

## Experimental section

All experimental details are provided in the ESI† with the original data.

## Conflicts of interest

There are no conflicts to declare.

## Supplementary Material

RA-012-D1RA09087A-s001

RA-012-D1RA09087A-s002
