# Supplementary material for: Simple Zn(ii) complexes for the production and degradation of polyesters
Source: RSC Adv. 2022 Jan 7;12(3):1416–24. doi: 10.1039/d1ra09087a (PMC8979053; doi:10.1039/d1ra09087a)
Supplement: RA-012-D1RA09087A-s001 [file RA-012-D1RA09087A-s001.pdf]

Electronic Supplementary Material (ESI) for RSC Advances.
This journal is © The Royal Society of Chemistry 2021

Supporting information

Simple Zn(II) Complexes for the Production and Degradation of Polyesters

Jack Stewart,[a] Martin Fuchs,[b] Jack Payne,[a] Oliver Driscoll,[a] Gabrielle Kociok-Köhn, Benjamin Ward, Sonja Herres-Pawlis[b] and Matthew D. Jones*[a]

[a] Department of Chemistry, University of Bath, Claverton Down, Bath BA27AY, United Kingdom E-mail: mj205@bath.ac.uk

[b] Lehrstuhl für Bioanorganische Chemie, Institut für Anorganische Chemie, RWTH Aachen University Landoltweg 1, 52074 Aachen, Germany

[c] Dr. Benjamin D. Ward. Department of Chemistry, Cardiff University, Park Place, Cardiff, CF10 3AT, United Kingdom

General experimental methods 2-3

Ligand synthesis and characterisation 4-17

Complex synthesis and characterisation 18-31

DOSY NMR 32

Polymer characterisation

- *Homonuclear decoupled spectra* 33-34
- *GPC* 35-36
- *MALDI-ToF spectra* 36
- *White polymer example* 36
- *DSC* 37

Zn – S distance vs. TOF volcano plot 38

Raman kinetics 39-41

Degradation characterisation 42-43

QTAIM analysis 45

Crystallographic data 46

References 46

General experimental methods

All chemicals were commercially obtained from Sigma-Aldrich and used as received. This is with the exception of the *rac*-lactide, which was singly recrystallised from dry toluene and stored under argon. For the synthesis of metal complexes under anhydrous conditions, dry solvents, MBraun LABmaster dp glovebox, standard Schlenk line techniques and oven-dried glassware were used. Dried and degassed reaction solvents, used in the preparation of these complexes, were collected under inert gas conditions from a Solvent Purification System (SPS).

^{1}H NMR spectra of ligands, complexes and polymerisations were recorded on a Bruker 400 II MHz or 500 MHz instrument and referenced to residual solvent peaks. Polymerisation conversion was recorded from the integration of the methine region of the polymer (5.12 - 5.20 ppm) against that of the monomer (4.94 – 5.01). The tacticity of polymers was determined from its ^{1}H{^{1}H} NMR spectrum, decoupling from the polymer doublet at 1.62 ppm. ^{1}H{^{1}H} NMR was recorded on a Bruker AV 400 MHz spectrometer. The following abbreviations are used in the report of spectra: s, singlet; d, doublet; dd, doublet of doublets; t, triplet; q, quartet; m, multiplet.

Electrospray ionisation (ESI) mass spectra of ligands were collected using a MicroToF electrospray quadrupole time-of-flight mass spectrometer, with the sample dissolved in acetonitrile at approximately 1 μgmL^{-1} concentration. Spectra were recorded in positive loop injection mode set for a range of 50 - 1500 m/z.

Typical polymerisation procedure (melt, zinc complexes): lactide (0.4 g, 2.8 x 10^{-3} mol) was added to an ampule with a Young's cap in 4 mL of toluene, with initiator (2.8 × 10^{-5} mol), benzyl alcohol (2.88 μL, 2.8 × 10^{-5} mol), and triethylamine (3.87 μL, 2.8 × 10^{-5} mol). An oil bath was heated to the appropriate temperature and the polymerisation ran for the chosen time. Once complete the solvent was immediately removed in vacuo and the crude product analysed via ^{1}H NMR. The pure polymer was obtained by washing with > 30 mL methanol.

Degradation reactions were performed in a Young's ampoule under argon. The flask containing PLA (0.25 g, Vegware™, PLLA cup, M_n = 45,510 g mol^{-1}), was taken into a glovebox and loaded with metal complex (8 wt%, 0.02 g). The polymer was then dissolved in THF (4 mL) with heating and stirring assisting dissolution. The flask was then submerged in a preheated oil bath (80 °C) to which MeOH (1 mL) was added. Aliquots were taken for ^{1}H NMR ($CDCl_3$) analysis of the methine region. After the reaction, the solvent was removed in vacuo and the residual methyl lactate (Me-La) was analysed further. PET reactions were carried out in a Young's ampoule containing 0.25 g of carbonated drinks bottle or thin films. Catalyst (8 wt%, 0.02 g) was added in a glove box. EG (27.5 eq, 1.5 mL) was added and the flask was submerged in a pre-heated oil bath at 180 °C. when full dissolution of the PET was observed, water was added and the mixture was filtered. BHET crystallised from the mixture and was collected, dried at 100°C *in vacuo* for 4 hours and weighed to obtain isolated yields.

GPC was carried out on an Agilent 1260 Infinity series instrument at 1 mL min^{-1} at 35 °C with a THF eluent using a PL gel 5 μm MIXED-D 300 x 7.5 mm column. Detection was carried out using a differential refractive index detector (referenced to 11 polystyrene standards of narrow molecular weight, ranging from M_w 615-568000 Da).

MALDI-ToF analysis was carried out on a Bruker Autoflex speed instrument in reflector positive mode, using DCTB as the matrix at a concentration of 10 mg mL^{-1}. 50 μL of this solution was co-applied with 2 μL of 0.1 M NaTFA solution and 10 μL of the analyte at a concentration of 10 mg mL^{-1}. 1 μL of this homogenised solution was applied to a steel target plate for analysis. Materials characterization (GPC, MALDI-ToF) facilities were provided through MC^2 at the University of Bath.

The DSC analyses were recorded on a TA Instruments DSC Q20. The sample was held at 40 °C for 1 minute, heated to 200 °C at 10 °C/min held at this temperature for 1 minute, cooled to 40 °C at 5 °C/min held at this temperature for 1 minute and finally heated to 200 °C at 10 °C/min - the T_m values are quoted for the second heating cycle. TGA analysis was recorded on a Setaram KEP Technologies Setsys Evolution TGA-DTA/DSC instrument. The sample chamber was purged with Ar (20 mL min^{-1}) for 20 minutes before being heated to 500 °C at 10 °C/min.

All crystallographic data was collected on a SuperNova or Excalibur, EOS detector diffractometer using radiation CuKα (λ = 1.54184 Å) or Mo-Kα (λ = 0.71073 Å) radiation all recorded at 150(2) K. All structures were solved by direct methods and refined on all F^2 data using the SHELXL-2014 suite of programs. All hydrogen atoms were included in idealised positions and refined using the riding model, all refinement details are given in the .cif file.

Experimental procedure for Raman kinetics

All polymerisation samples were prepared in a N_2 filled glovebox. The catalysts, co-initiator (4-MeBnOH or BnOH) and the lactide were stored in the same glovebox. Technical grade L- and D-lactide were stored at -30 °C. Technical grade lactide was used without further purification. L-lactide was recrystallised one time from toluene and dried under vacuum before use. All polymerisations were carried out under Ar atmosphere in a stainless-steel reactor. The reactor was heated to 150 °C under vacuum and flushed with Ar three times prior to the reaction. In all cases a stirrer speed of 260 rpm was used. All

polymerisations were monitored by *in situ*-Raman spectroscopy using a Kaiser Optical Systems RXN1 spectrometer equipped with a probe head with a sapphire lens (d = 0.1 mm) at a wavelength of 785 nm and 450 mW. *Peaxact 4* was used to calculate the kinetic data by integration of the Raman spectrum. The boundaries for lactide were 627 – 713 cm^{-1}. To determine the average molar masses and the mass distributions of these polylactide samples gel permeation chromatography (GPC) was used. A GPCmax VE 2001 from Viscotek was used with THF as the mobile phase and a flow rate of 1 $mlmin^{-1}$, combining an HPLC pump with two Malvern Viscotek T columns (porous styrene divinylbenzene copolymer) with a maximum pore size of 500 and 5000 a, a refractive index detector (VE 3580) and a viscometer (Viscotek 270 Dual Detector).

Sample preparation:

a) Polymerisation of technical grade *rac*-lactide:
Technical grade *rac*-lactide (4.0 g, 27.75 mmol, 1250 eq), technical grade *rac*-lactide (4.0 g, 27.75 mmol, 1250 eq) and the respective catalyst (0.022 mmol, 1.0 eq) were weighed out and thoroughly combined using an agate mortar. The mixture was placed in a screw cup vial and transferred to the reactor under Ar flow. As soon as the reactor was closed the Raman measurements were started. Spectra collection was conducted every 15 seconds. After 3 hours of reaction time the heating and stirring were stopped and the reaction mixture was exposed to air to end the polymerisation. An aliquot of the mixture was used to determine the conversion *via* ^{1}H-NMR spectroscopy in $CDCl_3$. A further portion of the reaction mixture was dissolved in CH_2Cl_2 (2.0 ml) and PLA was precipitated from cold EtOH (0 °C). The polymer was dried in vacuum and analysed *via* GPC.

b) Polymerisation of recrystallised l-lactide:
Recrystallised l-lactide (8.0 g, 55.5 mmol, 2500 eq),) and the respective catalyst (0.022 mmol, 1.0 eq) were weighed out and thoroughly combined using an agate mortar. The mixture was placed in a screw cup vial and transferred to the reactor under Ar flow. As soon as the reactor was closed the Raman measurements were started. Spectra collection was conducted every 15 seconds. After 3 hours of reaction time the heating and stirring were stopped and the reaction mixture was exposed to air to end the polymerisation. An aliquot of the mixture was used to determine the conversion *via* ^{1}H-NMR spectroscopy in $CDCl_3$. A further portion of the reaction mixture was dissolved in CH_2Cl_2 (2.0 ml) and PLA was precipitated from cold EtOH (0 °C). The polymer was dried in vacuum and analysed *via* GPC.

c) Polymerisation of recrystallised L-lactide with added co-initiator (4-MeBnOH):
Recrystallised L-lactide (8.0 g,55.5 mmol, 3000 eq),) 4-MeBnOH (22.6 mg, 0.19 mmol, 10 eq) and the respective catalyst (0.019 mmol, 1.0 eq) were weighed out and thoroughly combined using an agate mortar. The mixture was placed in a screw cup vial and transferred to the reactor under Ar flow. As soon as the reactor was closed the Raman measurements were started. Spectra collection was conducted every 15 seconds. After 3 hours of reaction time the heating and stirring were stopped and the reaction mixture was exposed to air to end the polymerisation. An aliquot of the mixture was used to determine the conversion *via* ^{1}H-NMR spectroscopy in $CDCl_3$. A further portion of the reaction mixture was dissolved in CH_2Cl_2 (2.0 ml) and PLA was precipitated from cold EtOH (0 °C). The polymer was dried in vacuum and analysed *via* GPC.

d) Polymerisation of recrystallised L-lactide with added co-initiator (BnOH)
Recrystallised l-lactide (8.0 g, 55.5 mmol, 3000 or 10000 eq) and $Zn(7)_2$ (1.0 eq) were weighed out and placed in a screw cap vial. The mixture was transferred to the reactor und Ar flow. The reactor was closed and the mixture was allowed to melt. After 1 min BnOH (10.0 or 100.0 eq) was added thereto and the Raman measurement was started. Spectra collection was conducted every 15 seconds. After the desired reaction time the heating and stirring was stopped and the reaction mixture was exposed to air to end the polymerisation. An aliquot of the mixture was used to determine the conversion *via* ^{1}H-NMR spectroscopy in $CDCl_3$. A further portion of the reaction mixture was dissolved in CH_2Cl_2 (2.0 ml) and PLA was precipitated from cold EtOH (0 °C). The polymer was dried in vacuum and analysed *via* GPC.

Ligand synthesis and characterisation

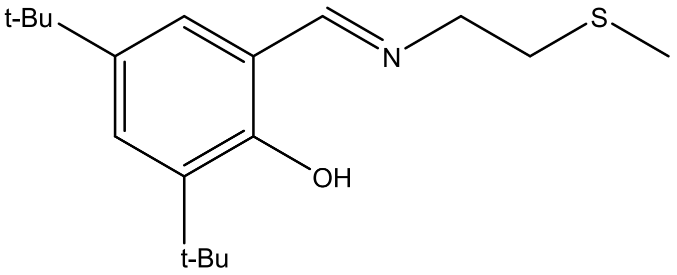

1H A solution of 3,5-di-*tert*-butyl-2-hydroxybenzaldehyde (2.34 g, 10 mmol) and 2-(methylthio) ethylamine (0.91 g, 10 mmol) in methanol (30 mL) was stirred at room temperature for two hours. The solvent was removed *in vacuo* to give a yellow oil. The crude product was recrystallised from methanol to give a yellow powder (2.21 g, 72%).

^{1}H NMR (400 MHz, C_6D_6) δ 14.07 (s, 1H, OH), 7.76 (s, 1H, CH), 7.58 (d, J = 2.5 Hz, 1H, Ar-H), 6.98 (d, J = 2.5 Hz, 1H, Ar-H), 3.25 (t, J = 6.9 Hz, 2H, CH_2), 2.32 (t, J = 6.9 Hz, 2H), 1.71 (s, 3H, CH_3), 1.66 (s, 9H, $C(CH_3)_3$), 1.33 (s, 9H, $C(CH_3)_3$).

^{13}C{^{1}H} NMR (101 MHz, $CDCl_3$) δ 166.9 (C=N), 158.1, 140.1, 136.7, 127.1, 126.0, 117.8 (Ar), 59.1, 35.2 (CH_2), 35.0, 34.1 (CH), 31.5, 29.5 (CH_3), 16.0 (S-CH_3).

m/z calc. $[C_{18}H_{30}NOS]^+$ (acetonitrile) = 308.2043, found = 308.2051.

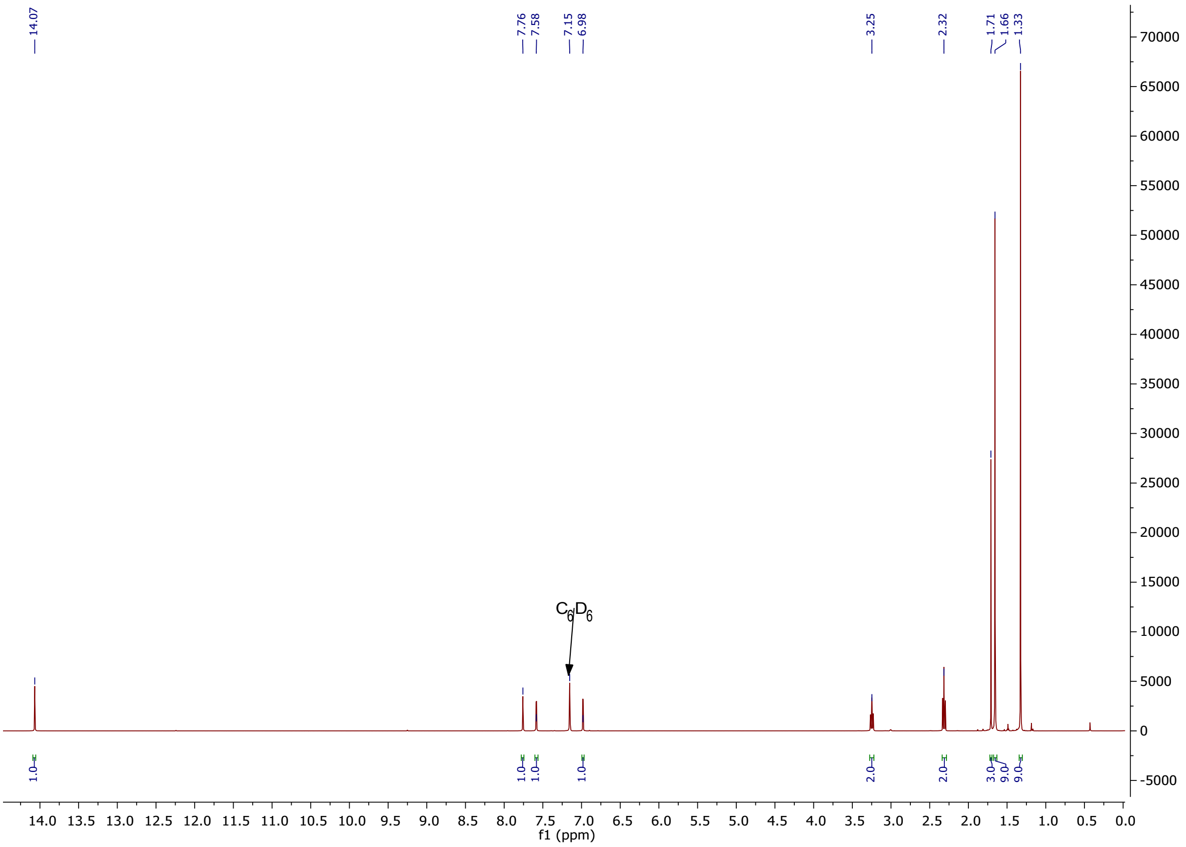

Figure SI1 ^{1}H NMR (400 MHz, C_6D_6, 298K) spectrum of **1**H.

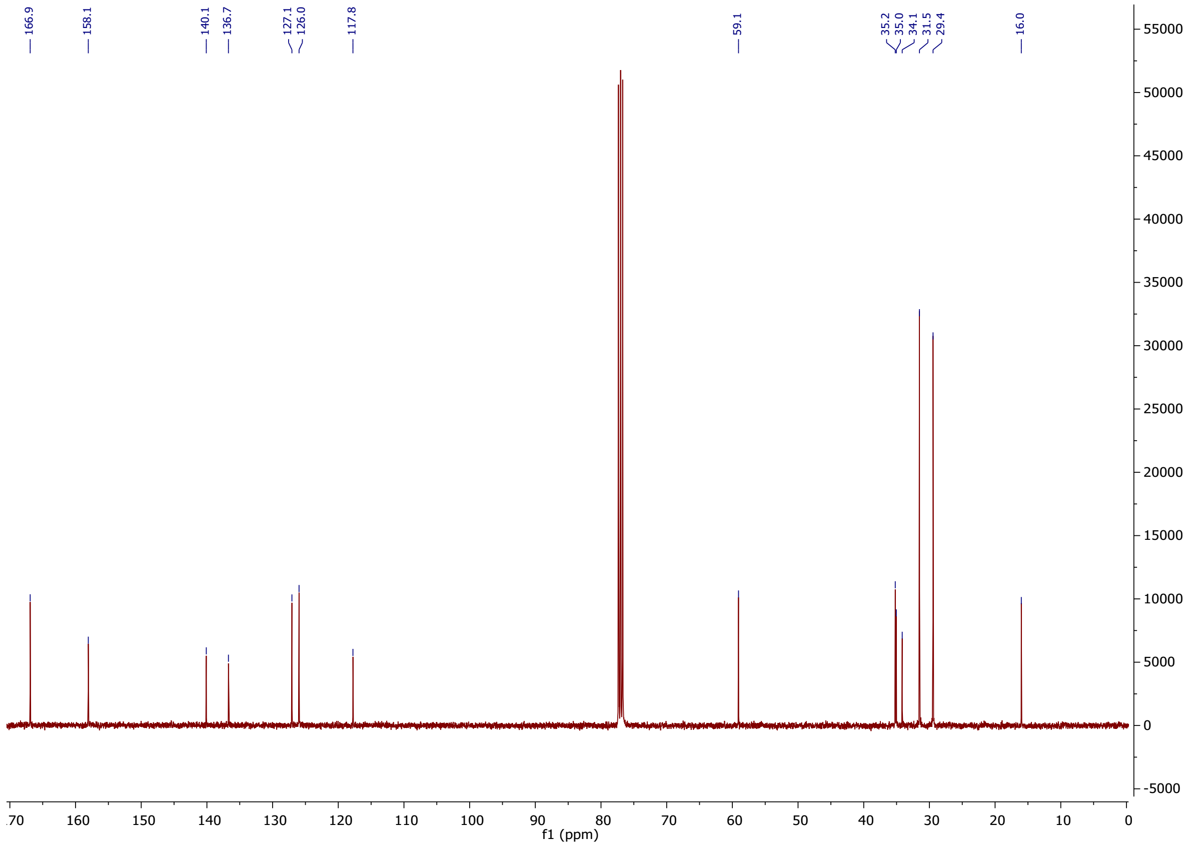

Figure SI2 $^{13}C\{^{1}H\}$ NMR (101 MHz, $CDCl_3$) of **1**H.

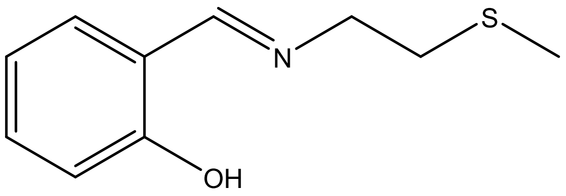

2H A solution of salicylaldehyde (1.22 g, 1.04 mL, 10.0 mmol) and 2-(methylthio) ethylamine (0.91 g, 10.0 mmol) in methanol (30 mL) was stirred at room temperature for two hours. The solvent was removed *in vacuo* to give a yellow oil (0.87 g, 45%).

^{1}H NMR (400 MHz, $CDCl_3$) δ 8.39 (s, 1H, CH), 7.33 (m, 1H, Ar-H), 7.30 – 7.27 (m, 1H, Ar-H), 6.98 (dd, J = 8.2, 1.0 Hz, 1H, Ar-H), 6.90 (td, J = 7.5, 1.1 Hz, 1H, Ar-H), 3.82 (t, J = 6.7 Hz, 2H, CH_2), 2.85 (t, J = 6.8 Hz, 2H, CH_2), 2.16 (s, 3H, CH_3).

$^{13}C\{^{1}H\}$ NMR (101 MHz, $CDCl_3$) δ 165.8 (C=N), 161.1, 132.4, 131.4, 118.7, 118.6, 117.0 (Ar), 59.0, 35.1 (CH_2), 16.0 (S-CH_3).

m/z calc. $[C_{10}H_{14}NOS]^{+}$ (acetonitrile) = 196.0791, found = 196.0803.

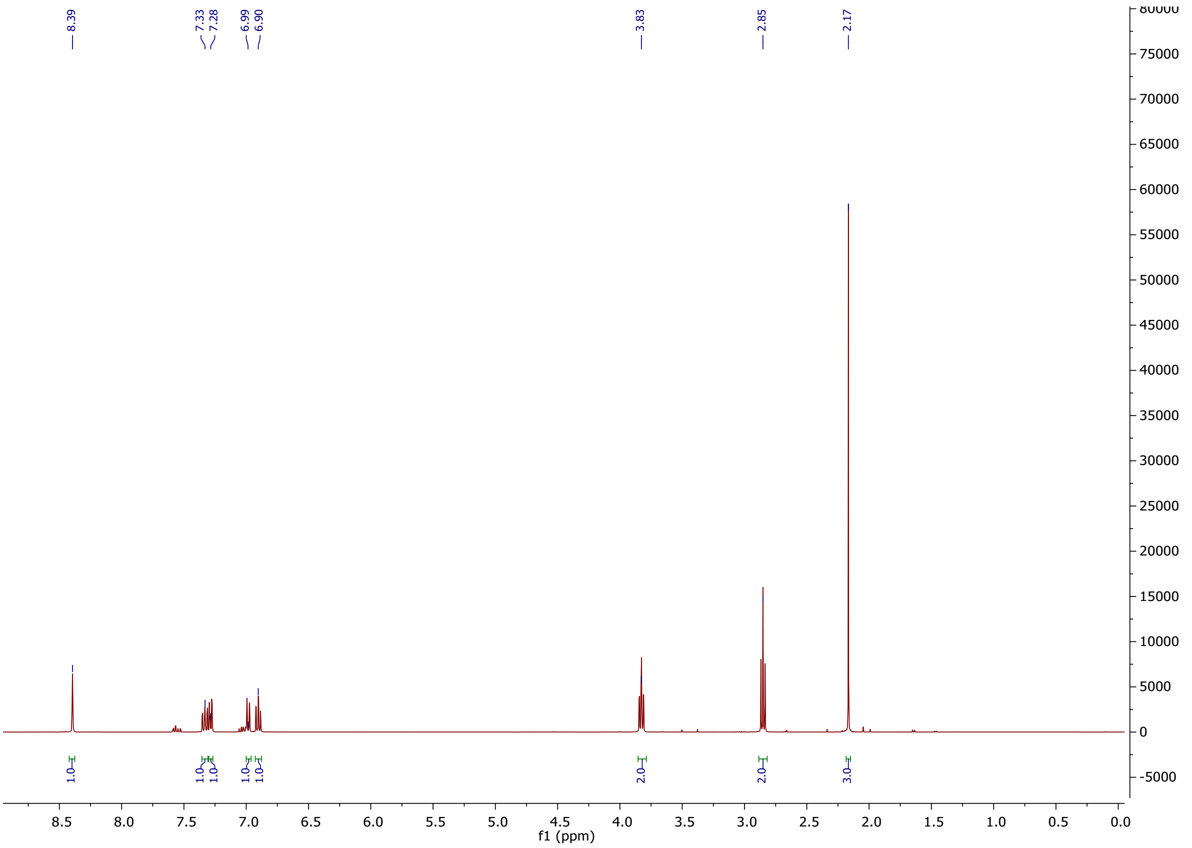

Figure SI3 ^{1}H NMR (400 MHz, $CDCl_3$, 298K) spectrum of **2**H.

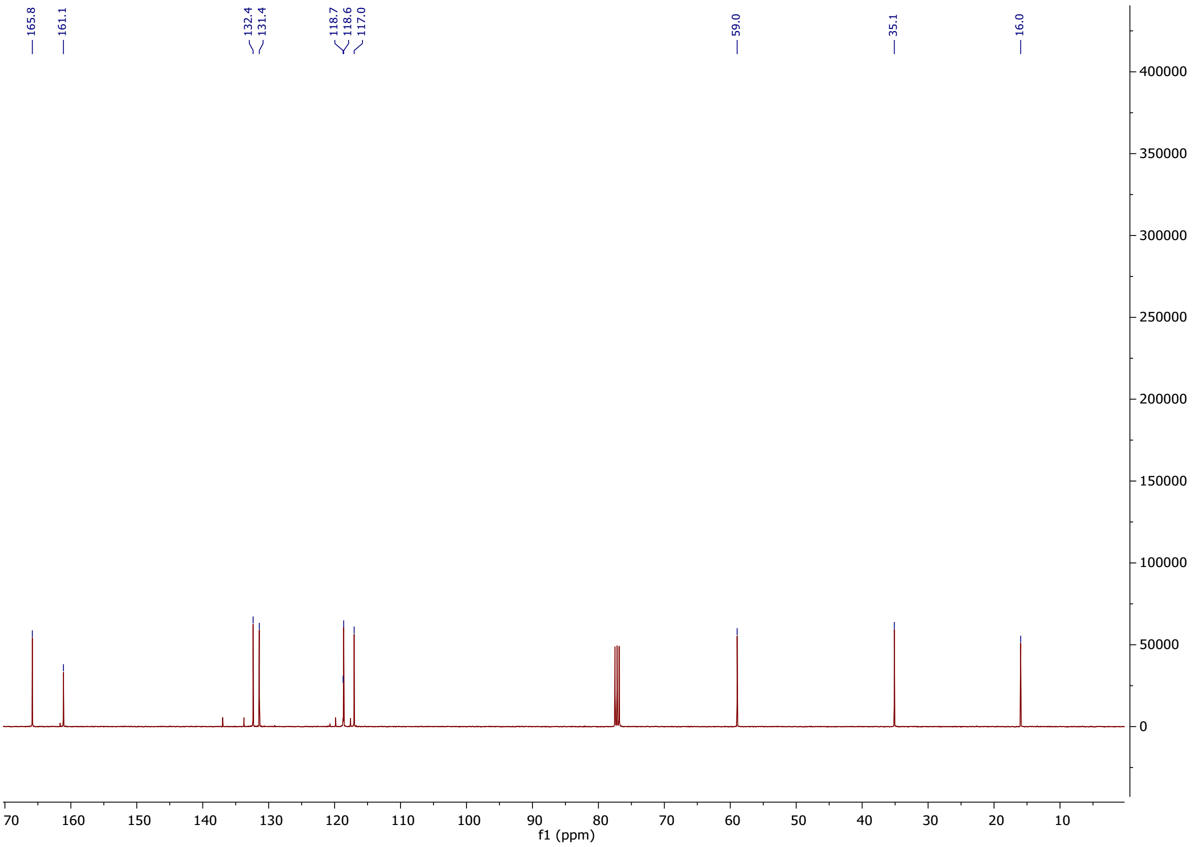

Figure SI4 $^{13}C\{^{1}H\}$ NMR (101 MHz, $CDCl_3$) of **2**H.

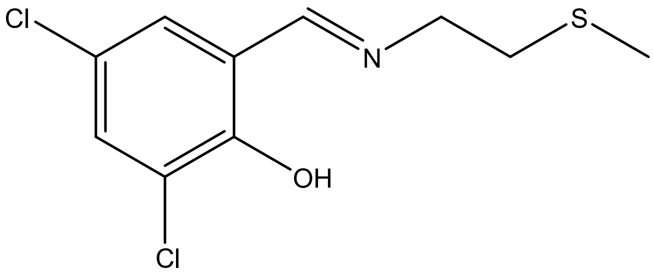

3H A solution of 3,5-dichloro-2-hydroxybenzaldehyde (1.91 g, 10.0 mmol) and 2-(methylthio) ethylamine (0.91 g, 10.0 mmol) in methanol (30 mL) was stirred at room temperature for two hours. The solvent was removed *in vacuo* to give a yellow oil. The crude product was recrystallised from methanol to give a yellow powder (1.81 g, 69%).

^{1}H NMR (400 MHz, $CDCl_3$) δ 8.30 (t, J = 1.2 Hz, 1H, CH), 7.43 (d, J = 2.5 Hz, 1H, Ar-H), 7.19 (d, J = 2.5 Hz, 1H, Ar-H), 3.89 – 3.84 (m, 2H, CH_2), 2.86 (t, J = 6.6 Hz, 2H, CH_2), 2.15 (s, 3H, CH_3).

$^{13}C\{^1H\}$ NMR (101 MHz, $CDCl_3$) δ 164.3 (C=N), 156.9, 132.3, 129.1, 123.0, 122.6, 119.3 (Ar), 57.9, 34.9 (CH_2), 15.9 (S-CH_3).

m/z calc. $[C_{10}H_{11}Cl_2NOS]^+$ (acetonitrile) = 264.0011, found = 264.0020.

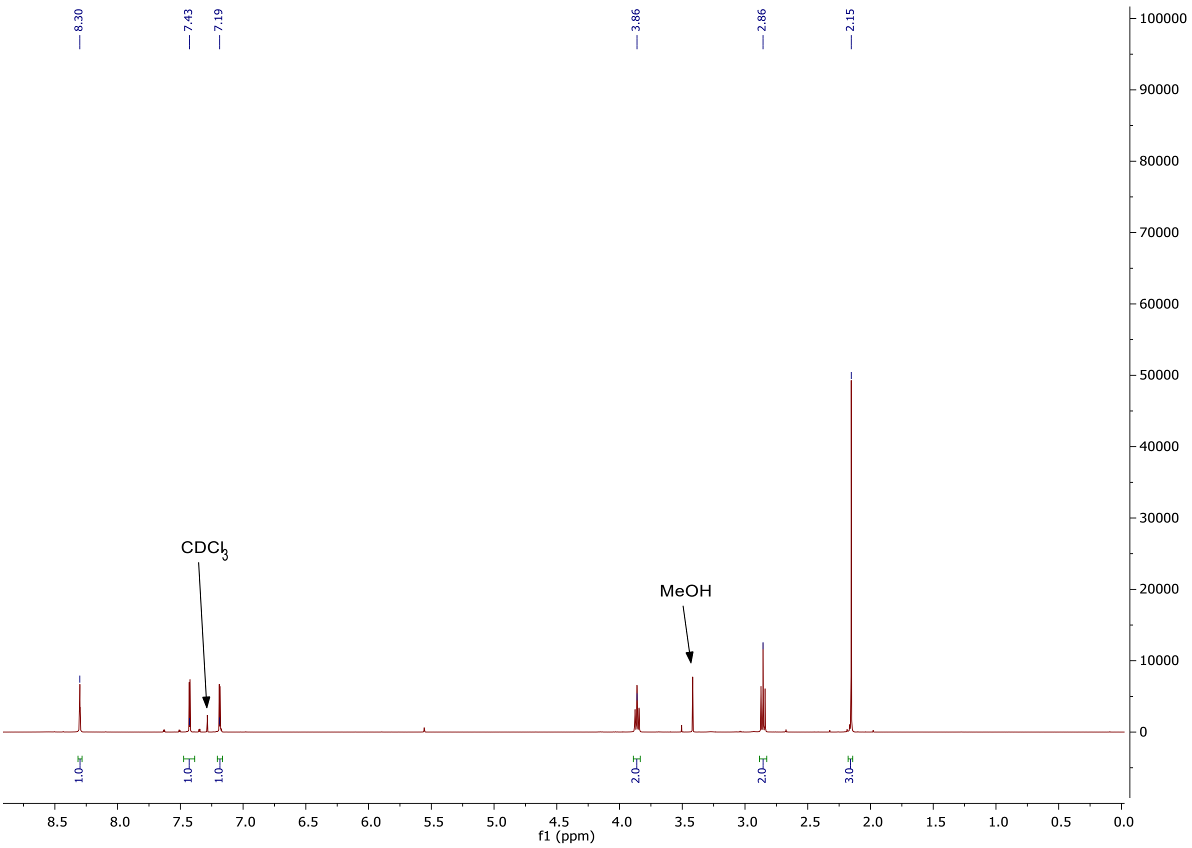

Figure SI5 ^{1}H NMR (400 MHz, $CDCl_3$) of **3**H.

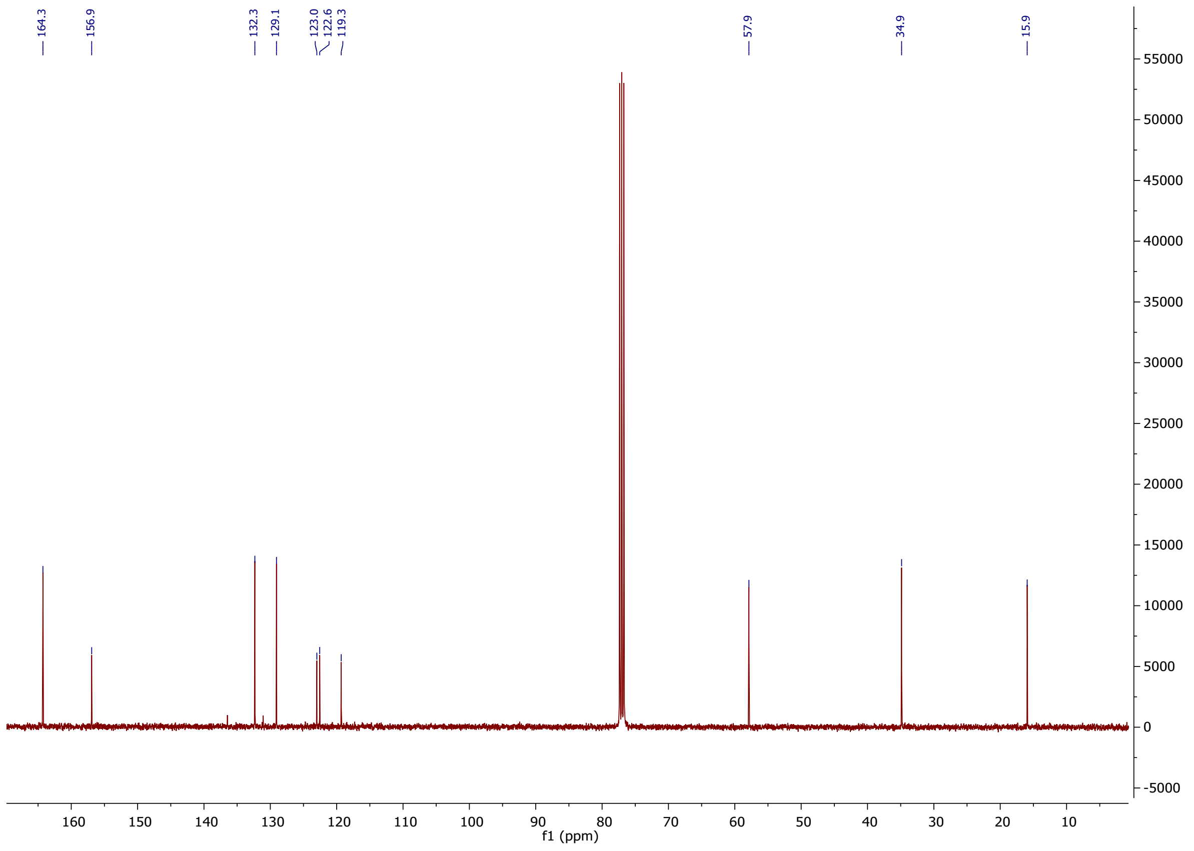

Figure SI6 $^{13}C\{^{1}H\}$ NMR (101 MHz, $CDCl_3$) of **3**H.

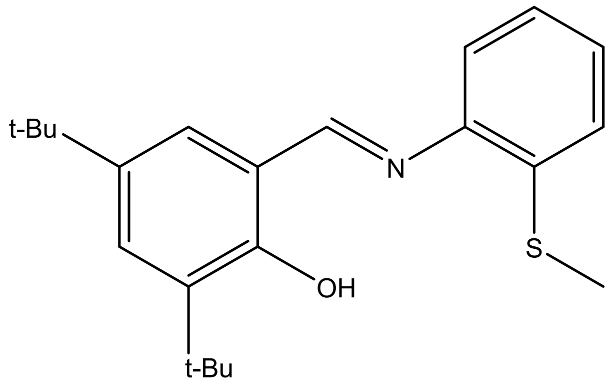

4H A solution of 3,5-di-*tert*-butyl-2-hydroxybenzaldehyde (2.34 g, 10.0 mmol) and 2-(methylthio) aniline (1.39 g, 1.25 mL, 10.0 mmol) in methanol (30 mL) was stirred at room temperature for two hours until a precipitate formed. The crude product was collected and recrystallised from methanol to give a yellow powder (3.12 g, 88%).

^{1}H NMR (500 MHz, $CDCl_3$) δ 8.55 (s, 1H, CH), 7.39 (d, J = 2.5 Hz, 1H, Ar-H), 7.17 (d, J = 4.7 Hz, 1H, Ar-H), 7.17 (d, J = 1.3 Hz, 1H, Ar-H), 7.15 (d, J = 2.4 Hz, 1H, Ar-H), 7.13 – 7.10 (m, 1H, Ar-H), 7.06 (dd, J = 7.5, 1.2 Hz, 1H, Ar-H), 2.40 (s, 3H, CH_3), 1.42 (s, 9H, $C(CH_3)_3$), 1.26 (s, 9H, $C(CH_3)_3$).

$^{13}C\{^{1}H\}$ NMR (126 MHz, $CDCl_3$) δ 163.3 (C=N), 158.4, 146.0, 140.5, 137.1, 134.6, 128.3, 127.0, 126.9, 125.2, 124.8, 118.4, 117.5 (Ar), 35.2 (CH_2), 34.2 (CH_2), 31.5 (CH_3), 31.3 (CH), 29.5 (CH_3), 29.3 (CH), 14.8 (S-CH_3).

m/z calc. $[C_{22}H_{30}NOS]^+$ (acetonitrile) = 356.2043, found = 356.2061.

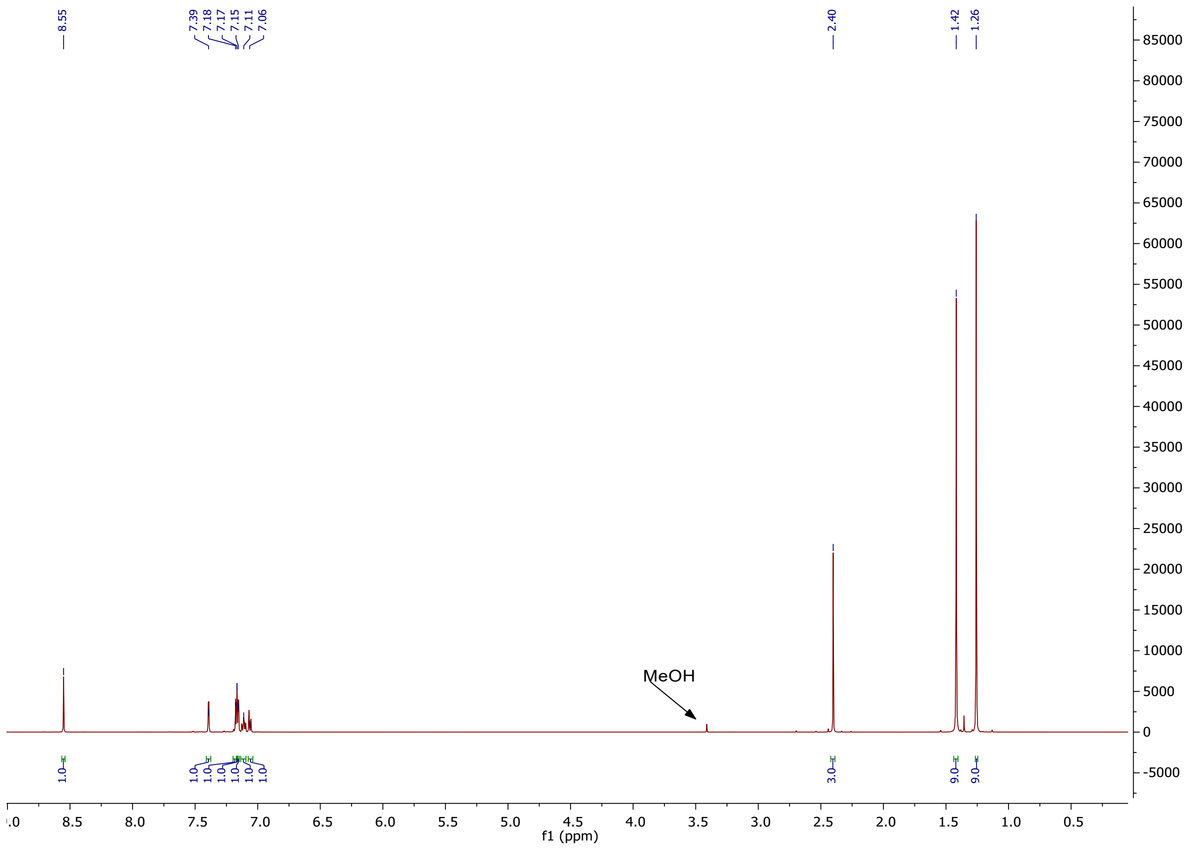

Figure SI7 ^{1}H NMR (500 MHz, $CDCl_3$) of **4**H.

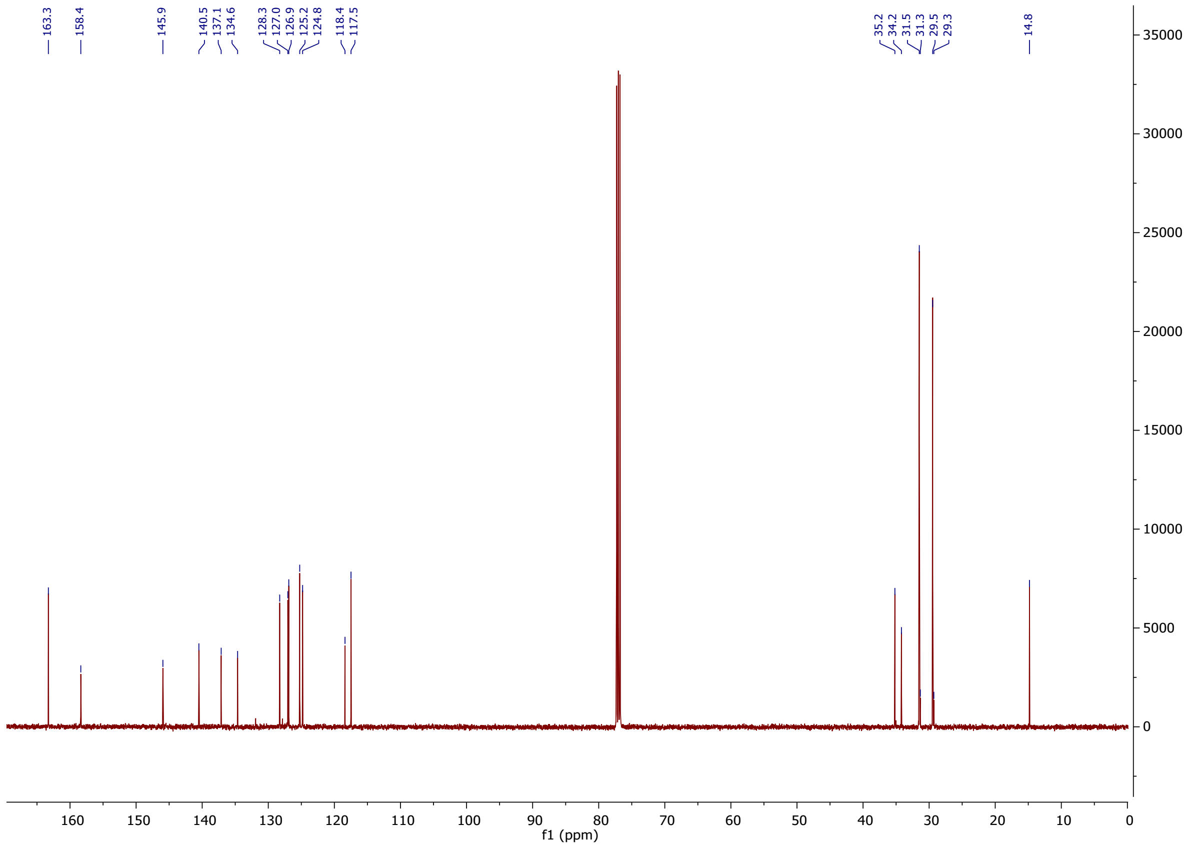

Figure SI8 $^{13}C\{^{1}H\}$ NMR (126 MHz, $CDCl_3$) of **4**H.

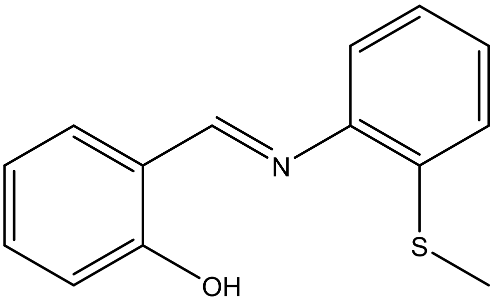

5H A solution salicylaldehyde (1.22 g, 1.04 mL, 10.0 mmol) and 2-(methylthio) aniline (1.39 g, 1.25 mL, 10.0 mmol) in methanol (30 mL) was stirred at room temperature for two hours. A yellow oil precipitated out of solution and was collected through separation. The crude product was washed with methanol to give a yellow oil which solidified overnight (1.74 g, 72%).

^{1}H NMR (500 MHz, $CDCl_3$) δ 8.56 (s, 1H, CH), 7.33 (m, 1H, Ar-H), 7.32 – 7.29 (m, 1H, Ar-H), 7.22 – 7.15 (m, 2H, Ar-H), 7.15 – 7.08 (m, 2H, Ar-H), 6.99 (dd, J = 8.3, 1.0 Hz, 1H, Ar-H), 6.86 (td, J = 7.5, 1.1 Hz, 1H, Ar-H), 2.40 (s, 3H, CH_3).

^{13}C{^{1}H} NMR (126 MHz, $CDCl_3$) δ 161.8 (C=N), 161.2, 145.3, 134.9, 133.4, 132.4, 127.5, 125.4, 125.1, 119.2, 119.1, 117.5, 117.2 (Ar), 14.9 (S-CH_3).

m/z calc. $[C_{14}H_{14}NOS]^+$ (acetonitrile) = 244.0791, found = 244.0788.

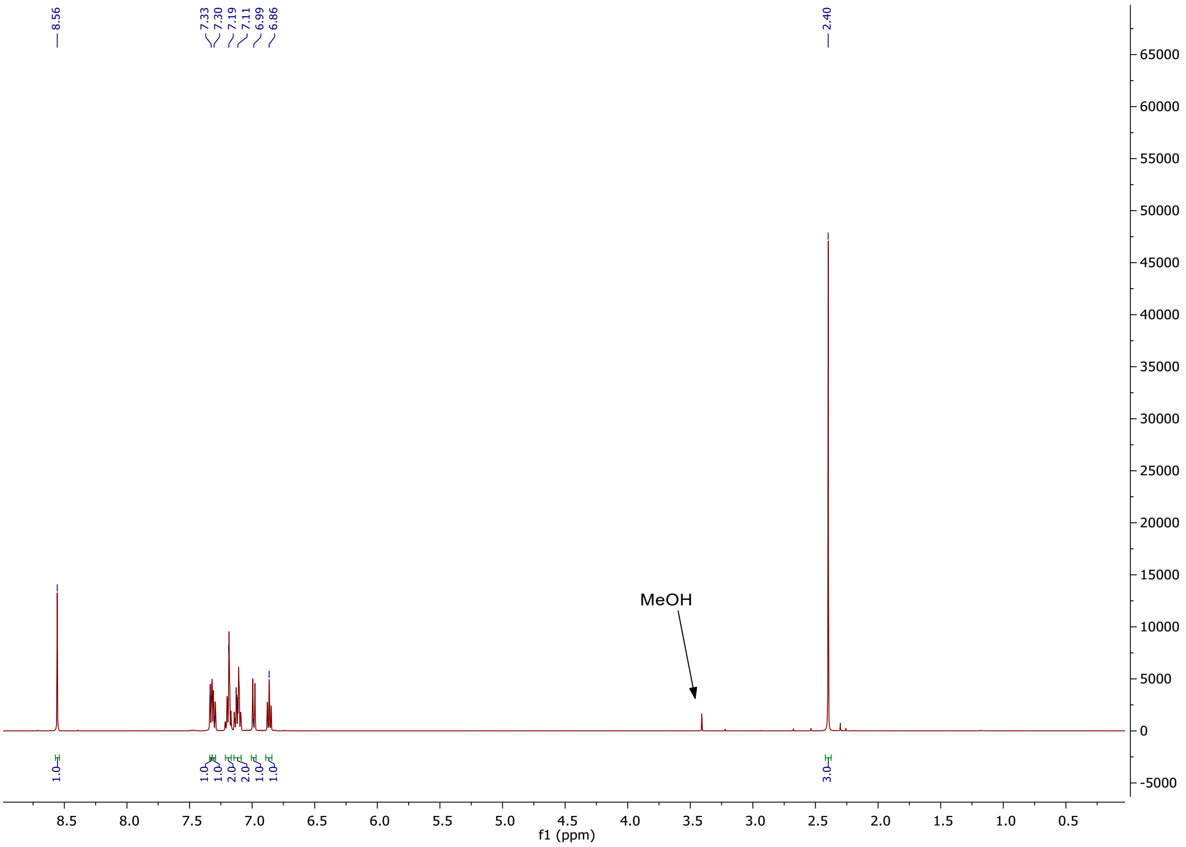

Figure SI9 ^{1}H NMR (500 MHz, $CDCl_3$) of **5**H.

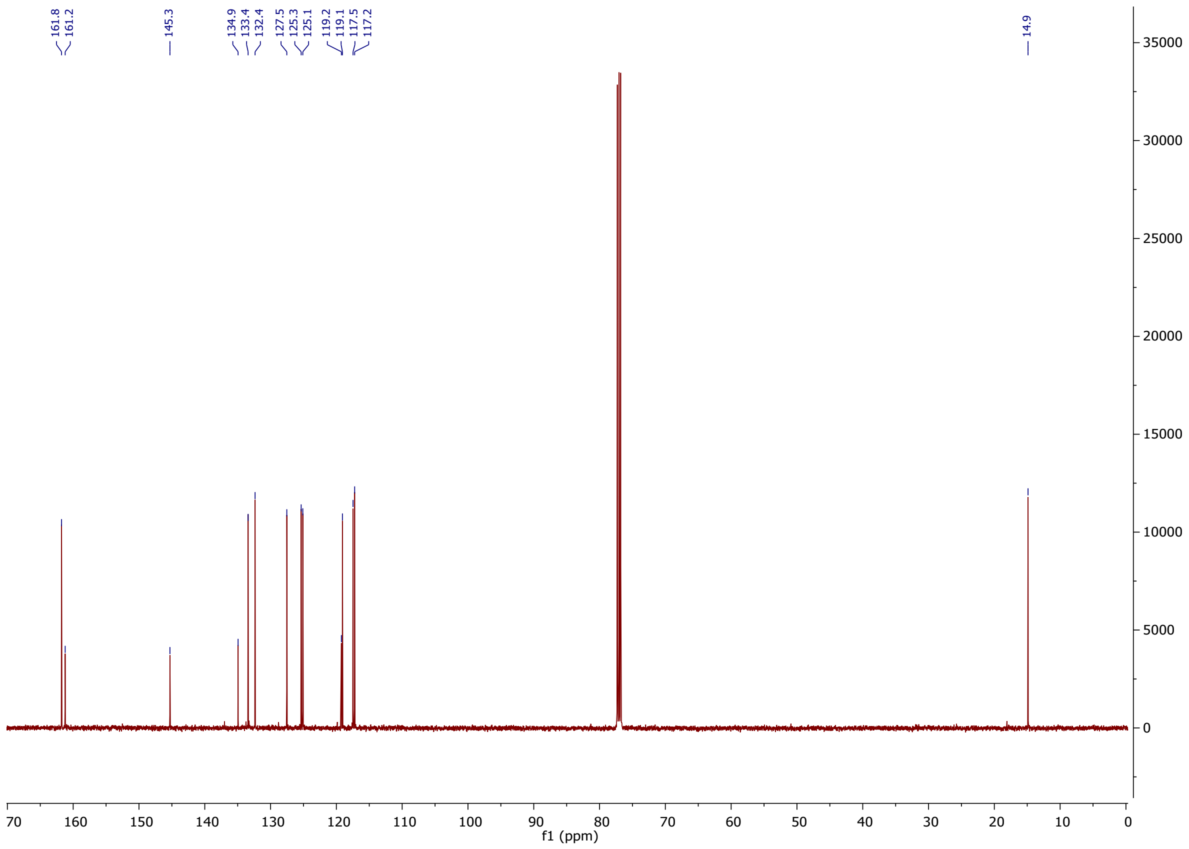

Figure SI10 $^{13}C\{^1H\}$ NMR (126 MHz, $CDCl_3$) of **5**H.

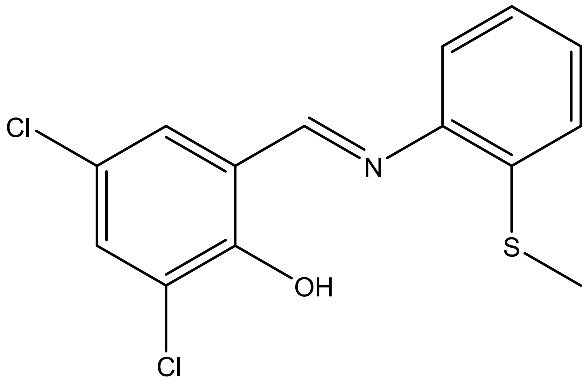

6H A solution of 3,5-dichloro-2-hydroxybenzaldehyde (1.91 g, 10.0 mmol)) and 2-(methylthio) aniline (1.39 g, 1.25 mL, 10.0 mmol) in methanol (30 mL) was stirred at room temperature for one minute before an orange precipitate was formed. He crude product was collected and recrystallised from methanol to give a bright orange product (1.5 g, 48%).

^1H NMR (400 MHz, $CDCl_3$) δ 8.66 (s, 1H), 7.48 – 7.36 (m, 2H), 7.26 – 7.15 (m, 2H), 7.09 (dd, *J* = 8.2, 1.1 Hz, 1H), 6.97 (td, *J* = 7.5, 1.1 Hz, 1H), 2.50 (s, 3H).

$^{13}C\{^1H\}$ NMR (126 MHz, $CDCl_3$) δ 161.8 (C=N), 161.2, 145.3, 134.9, 133.4, 132.4, 127.53, 125.4, 125.1, 119.2, 119.1, 117.5, 117.2 (Ar), 14.9 (S-CH_3).

m/z calc. $[C_{14}H_{11}Cl_2NOS]^+$ (acetonitrile) = 312.0011, found = 312.0006.

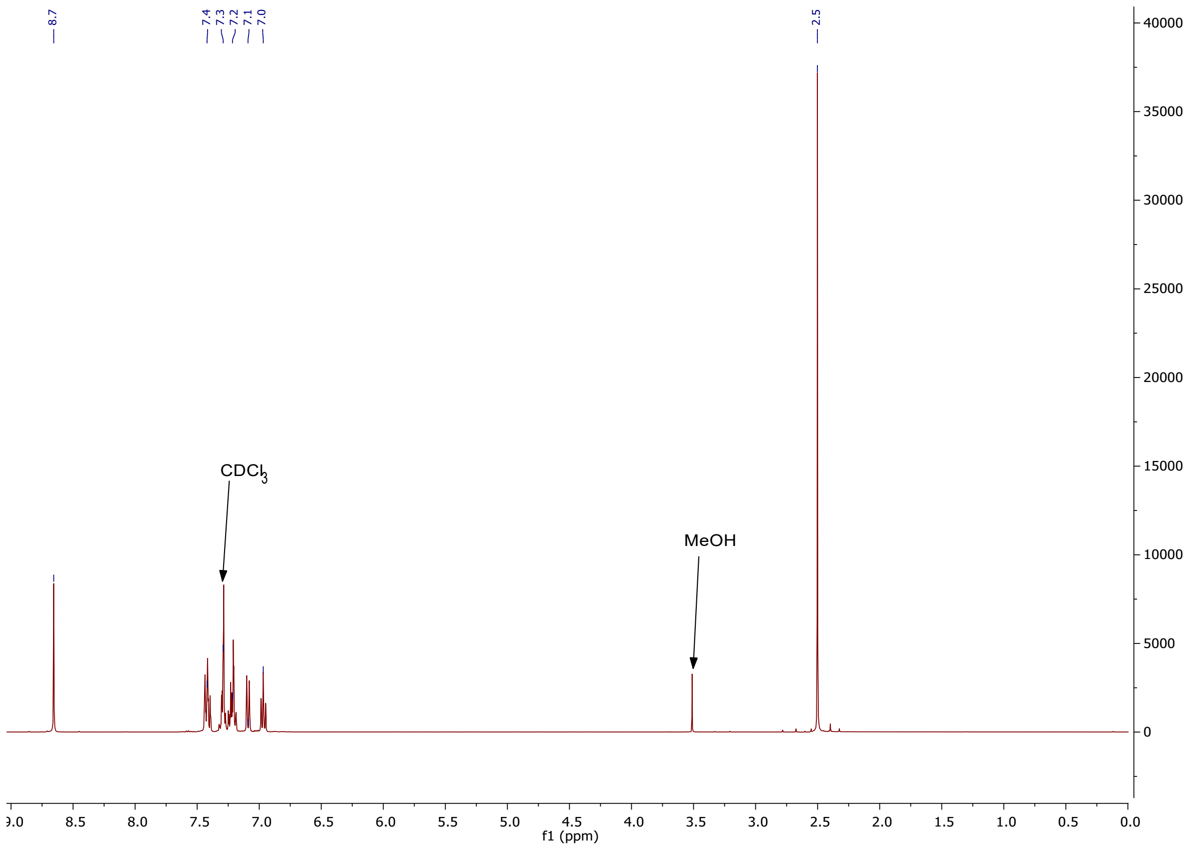

Figure SI11 ^{1}H NMR (400 MHz, $CDCl_3$) of **6**H.

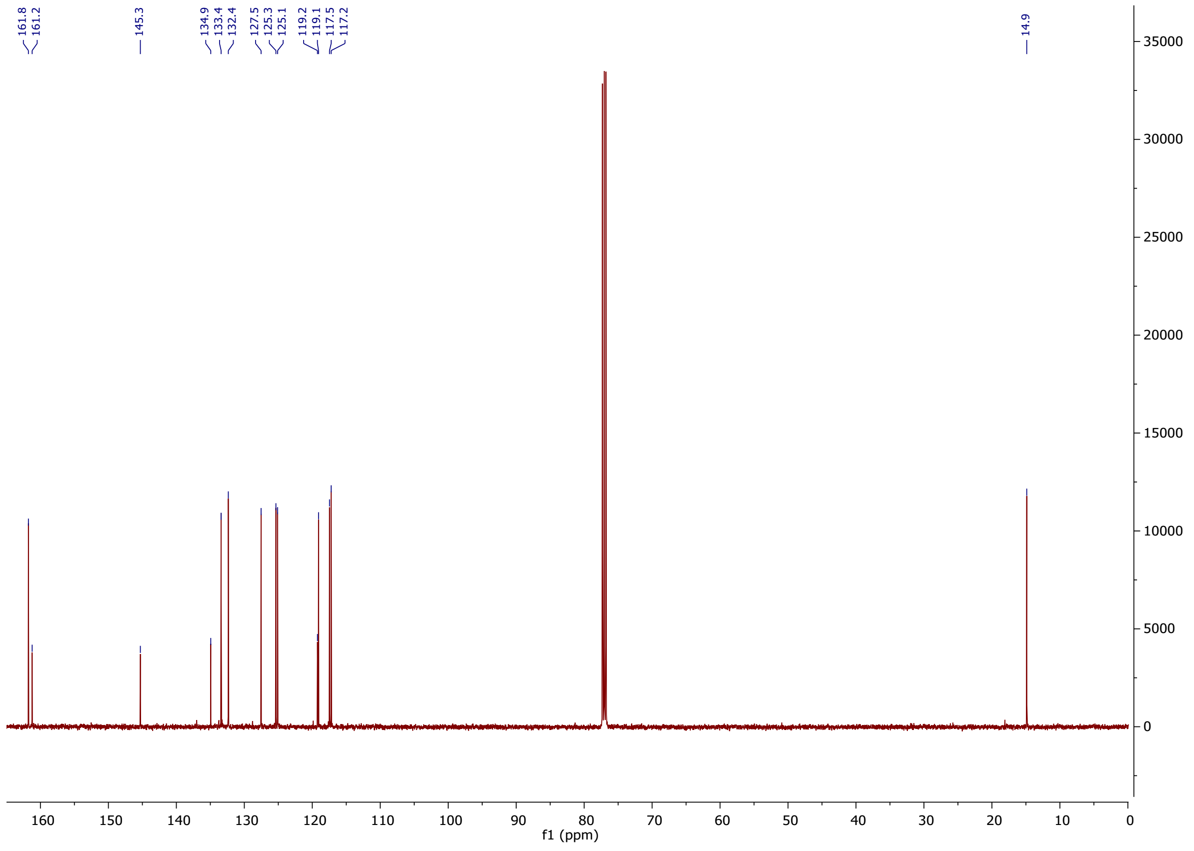

Figure SI12 $^{13}C\{^{1}H\}$ NMR (126 MHz, $CDCl_3$) of **6**H.

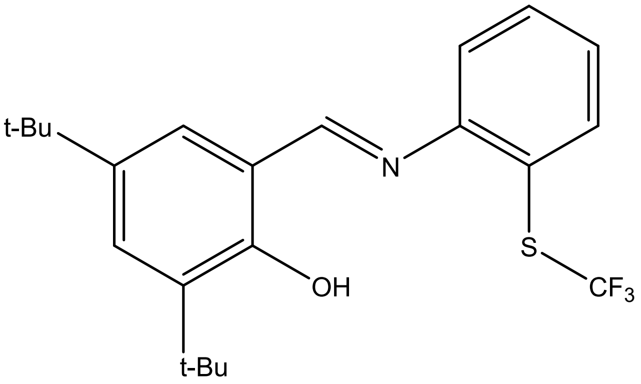

7H A solution of 3,5-di-*tert*-butyl-2-hydroxybenzaldehyde (2.34 g, 10.0 mmol) and 2-(trifluoromethylthio) aniline (1.93 g, 10.0 mmol) in methanol (30 mL) was stirred at room temperature for two hours. The solvent was removed *in vacuo* to give a yellow oil. The crude product was recrystallised from methanol to give a yellow powder (2.57 g, 63%).

^{1}H NMR (500 MHz, $CDCl_3$) δ 13.14 (s, 1H, OH), 8.50 (s, 1H, CH), 7.70 – 7.68 (m, 1H, Ar-H), 7.46 (td, J = 7.7, 1.5 Hz, 1H, Ar-H), 7.43 (d, J = 2.5 Hz, 1H, Ar-H), 7.23 (td, J = 7.7, 1.4 Hz, 1H, Ar-H), 7.18 – 7.17 (m, 1H, Ar-H), 1.42 (s, 9H, $C(CH_3)_3$), 1.26 (s, 9H, $C(CH_3)_3$).

^{13}C{^{1}H} NMR (126 MHz, $CDCl_3$) δ 165.2 (CH), 158.6, 152.2, 140.7, 137.6, 137.3, 132.4, 128.9, 127.1, 127.0, 119.6, 119.6, 119.5, 118.1 (Ar), 35.2, 34.2 (CH), 31.5, 29.4 (CH_3).

^{19}F NMR (471 MHz, $CDCl_3$) δ -41.80.

m/z calc. $[C_{22}H_{27}F_3NOS]^+$ (acetonitrile) = 410.1760, found = 410.1778.

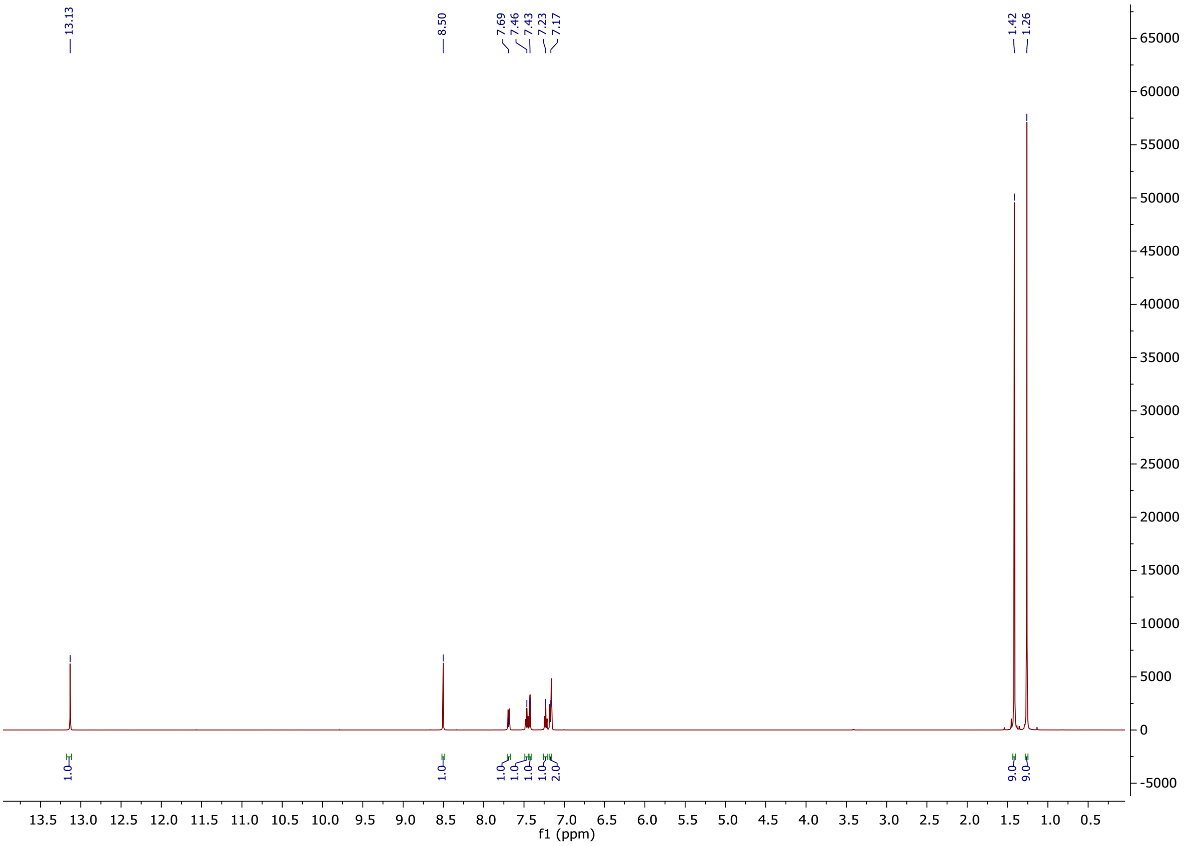

Figure SI13 ^{1}H NMR (500 MHz, $CDCl_3$) of **7**H.

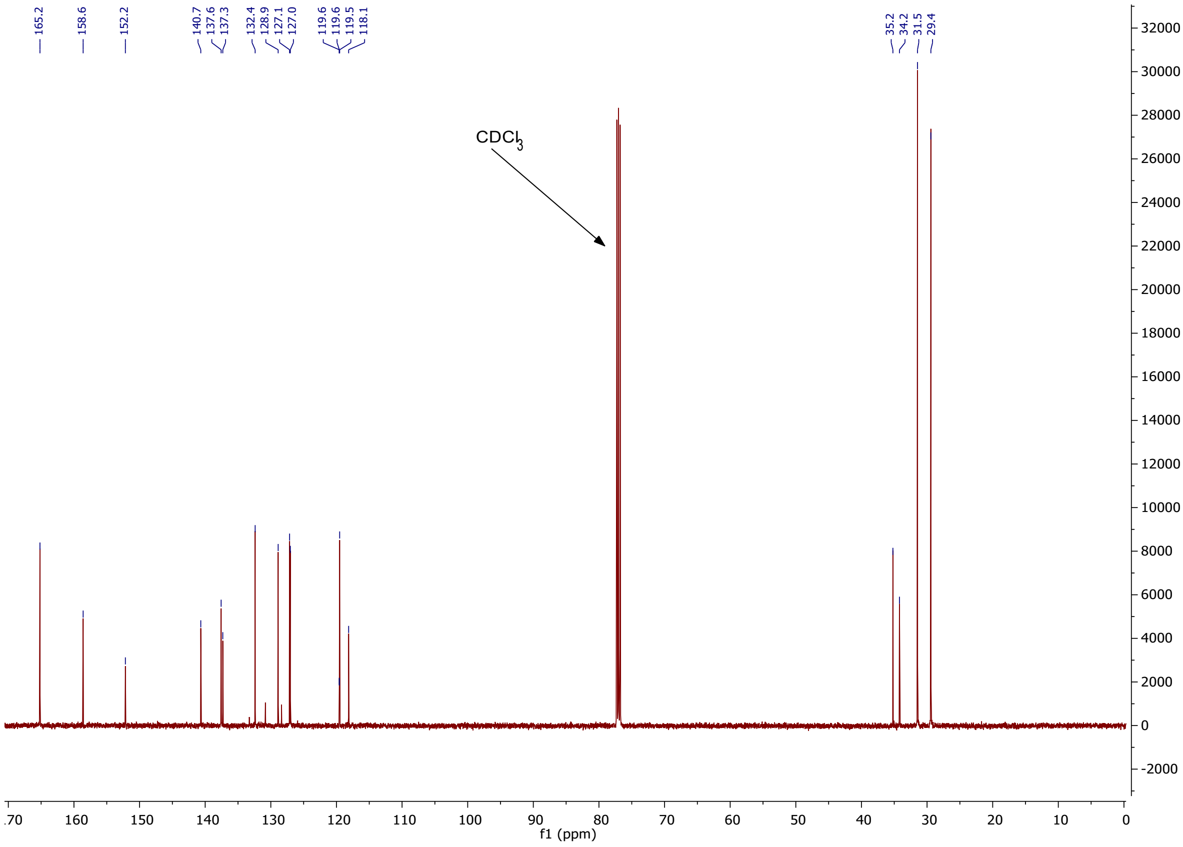

Figure SI14 $^{13}C\{^1H\}$ NMR (126 MHz, $CDCl_3$) of **7**H.

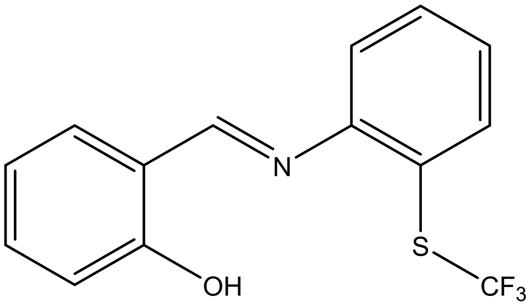

8H A solution of salicylaldehyde (1.22 g, 1.04 mL, 10.0 mmol) and 2-(trifluoromethylthio) aniline (1.93 g, 10.0 mmol) in methanol (30 mL) was stirred at room temperature for two hours. The solvent was removed *in* vacuo to give a pale-yellow oil. The crude product was washed with methanol (0.80 g, 22%).

^1H NMR (500 MHz, $CDCl_3$) δ 8.51 (s, 1H, CH), 7.71 (dd, J = 7.8, 1.4 Hz, 1H, Ar-H), 7.49 (td, J = 7.7, 1.5 Hz, 1H, Ar-H), 7.37 – 7.32 (m, 2H, Ar-H), 7.26 (td, J = 7.6, 1.3 Hz, 1H, Ar-H), 7.21 (dd, J = 8.1, 1.3 Hz, 1H, Ar-H), 6.99 (dd, J = 8.7, 1.0 Hz, 1H, Ar-H), 6.88 (td, J = 7.5, 1.1 Hz, 1H, Ar-H).

$^{13}C\{^1H\}$ NMR (126 MHz, $CDCl_3$) δ 163.9 (C=N), 161.4, 151.6, 138.0, 134.02, 132.7, 132.6, 127.5, 119.7, 119.3, 119.2, 118.0, 117.6 (Ar).

m/z calc. $[C_{14}H_{11}F_3NOS]^+$ (acetonitrile) = 298.0508, found = 298.0505.

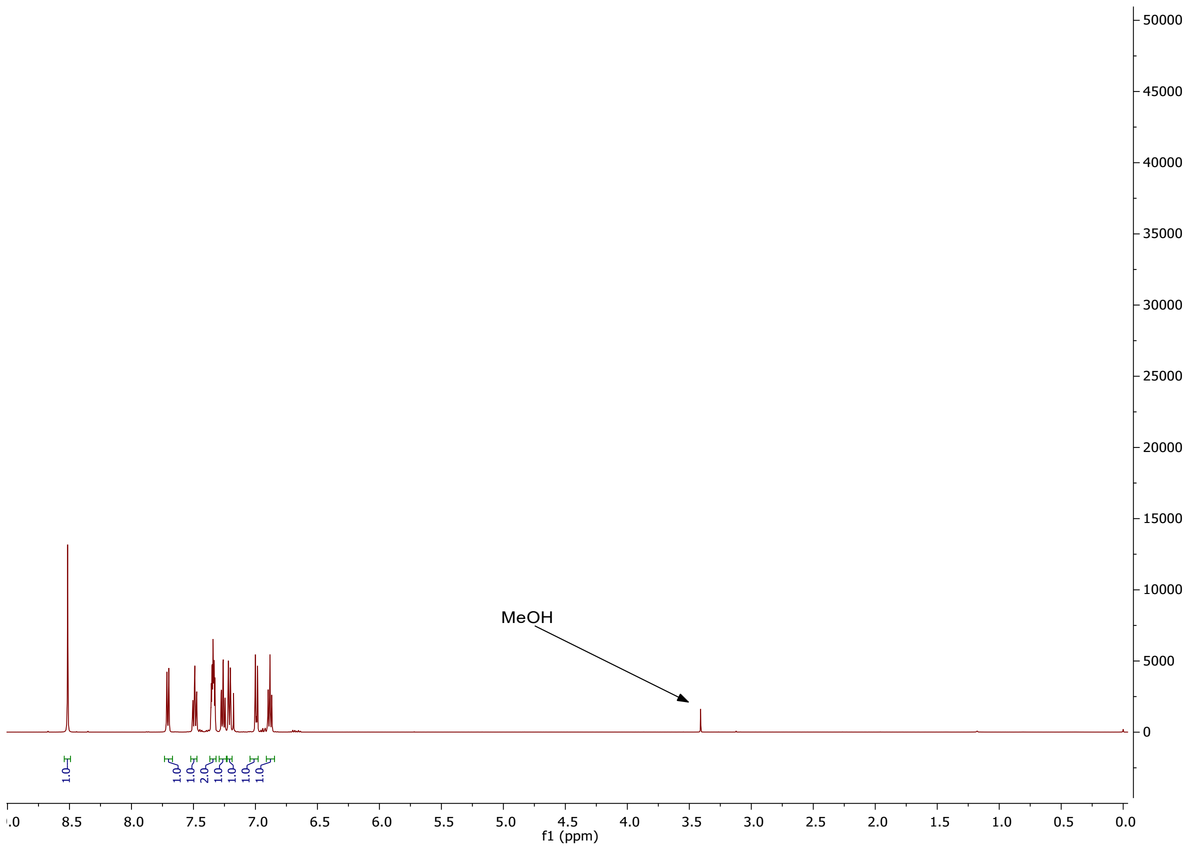

Figure SI15 ^{1}H NMR (500 MHz, $CDCl_3$) of **8**H.

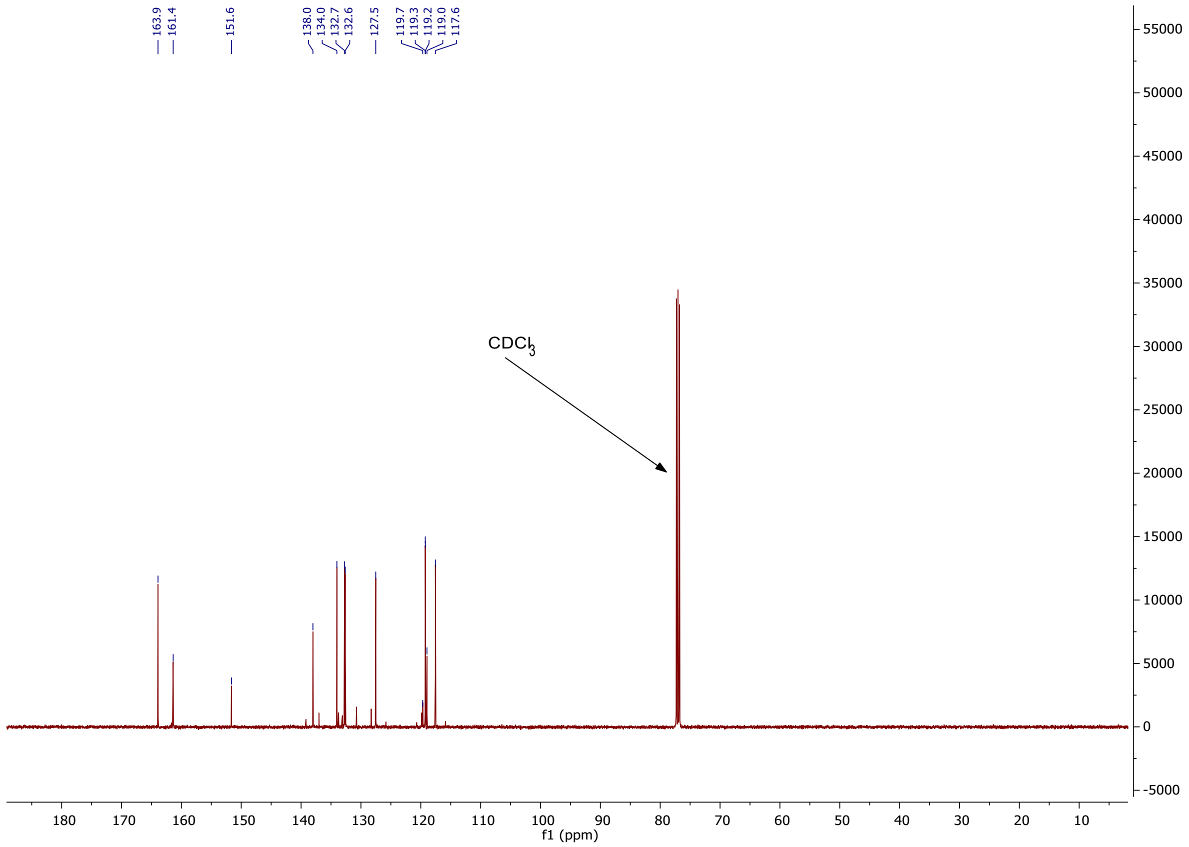

Figure SI 16 $^{13}C\{^{1}H\}$ NMR (126 MHz, $CDCl_3$) of **8**H.

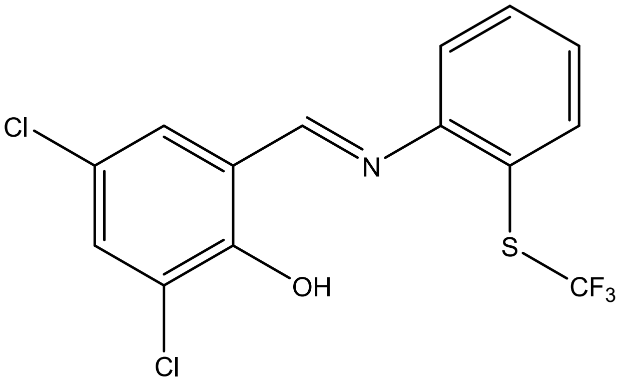

9H A solution of 3,5-dichloro-2-hydroxybenzaldehyde (1.91 g, 10.0 mmol) and 2-(trifluoromethylthio) aniline (1.93 g, 10.0 mmol) in methanol (30 mL) was stirred at room temperature for two hours until a precipitate formed. The crude product was collected and recrystallised from methanol to give a yellow powder (2.72 g, 74%).

^{1}H NMR (500 MHz, $CDCl_3$) δ 8.45 (s, 1H, CH), 7.73 (dd, J = 7.8, 1.3 Hz, 1H, Ar-H), 7.52 (td, J = 7.8, 1.5 Hz, 1H, Ar-H), 7.42 (d, J = 2.5 Hz, 1H, Ar-H), 7.32 (td, J = 7.6, 1.3 Hz, 1H, Ar-H), 7.25 (d, J = 2.5 Hz, 1H, Ar-H), 7.21 (dd, J = 7.9, 1.3 Hz, 1H, Ar-H).

^{13}C{^{1}H} NMR (126 MHz, $CDCl_3$) δ 162.0 (CH), 155.83, 150.7, 138.3, 133.4, 132.8, 130.2, 128.4, 123.7, 123.1, 120.2, 120.0, 119.2 (Ar).

m/z calc. $[C_{14}H_9F_3Cl_2NOS]^+$ (acetonitrile) = 365.9729, found = 365.9737.

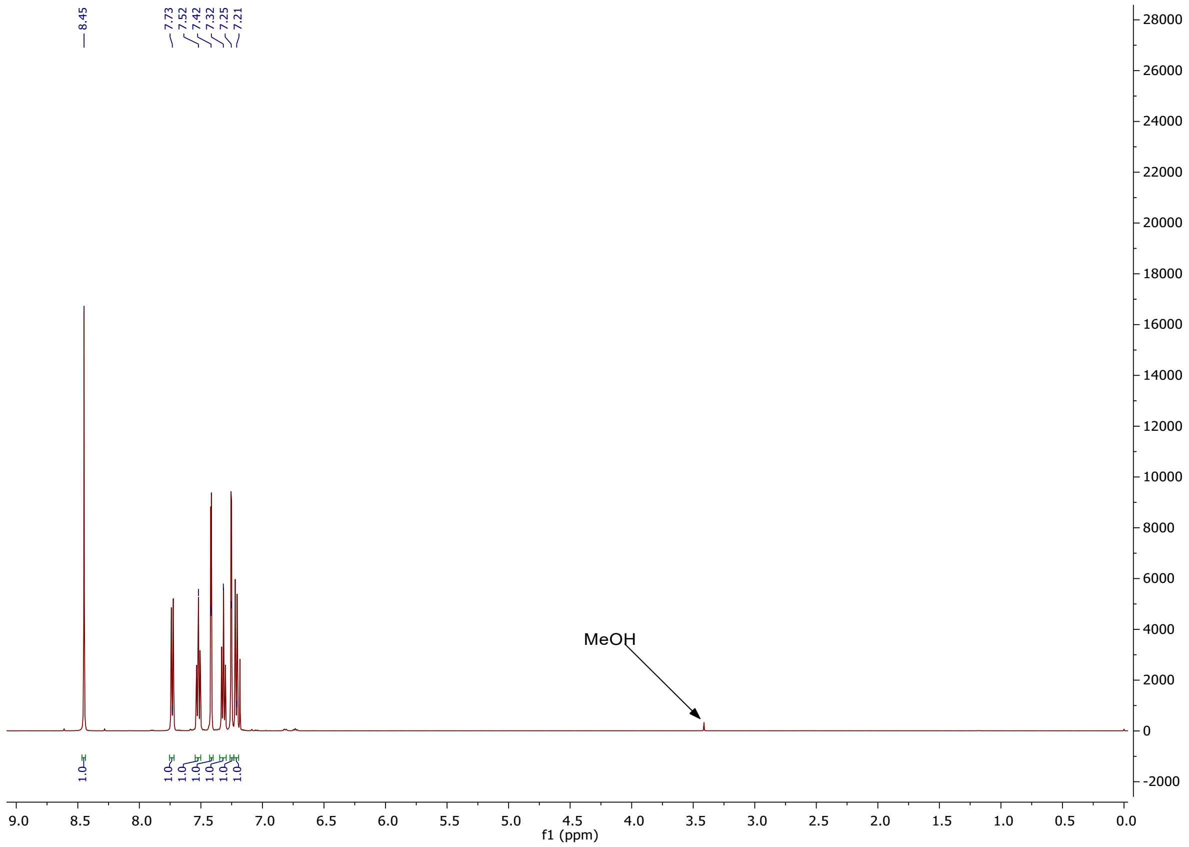

Figure SI17 ^{1}H NMR (500 MHz, $CDCl_3$ of **9**H.

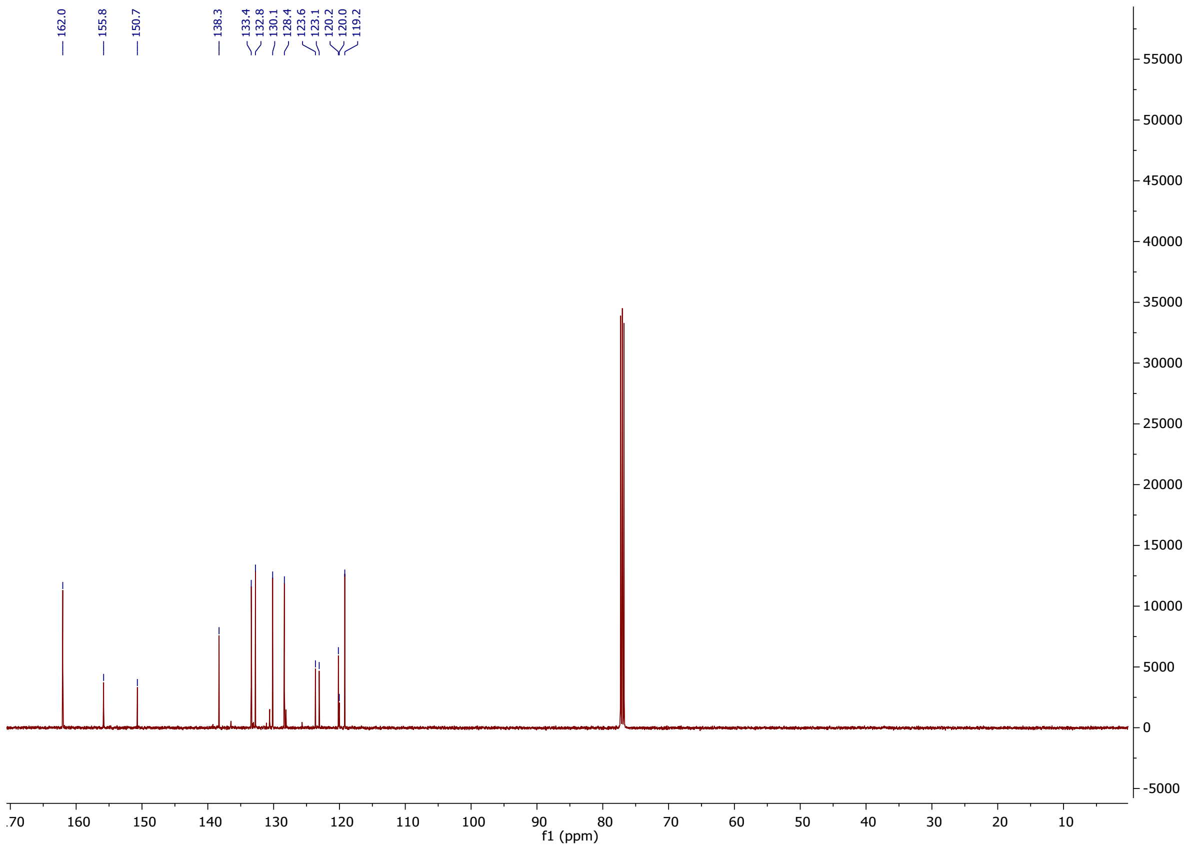

Figure SI18 $^{13}C\{^{1}H\}$ NMR (126 MHz, $CDCl_3$) of **9**H.

Complex synthesis and characterisation

Zn(**1**)$_2$ Ligand **1**H (2 mmol, 0.62 g) was dissolved in toluene (10 mL). $ZnEt_2$ (1 mmol, 1 mL, 1.0 M) was added dropwise and the solution stirred for two hours. The product crystallised from toluene as a pale-yellow solid (0.15 g, 22%).

^{1}H NMR (400 MHz, C_6D_6) δ 7.68 (d, *J* = 2.7 Hz, 1H, Ar-H), 7.62 (s, 1H, CH), 6.85 (d, *J* = 2.6 Hz, 1H, Ar-H), 3.32 (m, 2H, CH_2), 2.67 – 2.27 (m, 2H, CH_2), 1.70 (s, 9H, $C(CH_3)_3$), 1.57 (s, 3H, CH_3), 1.36 (s, 9H, $C(CH_3)_3$).

^{13}C{^{1}H} NMR (101 MHz, C_6D6) δ 172.6 (CH), 169.3, 141.7, 135.5, 130.0, 125.5, 117.5 (Ar), 59.6, 35.9 (CH_2), 34.7, 33.9 (CH), 31.6, 29.8 (CH_3), 15.1 (S-CH_3).

Elemental analysis ($C_{36}H_{56}N_2O_2S_2Zn$) Calcd in %: C, 63.75; H, 8.32; N, 4.13. Found: C, 61.56; H, 8.39; N, 3.87.

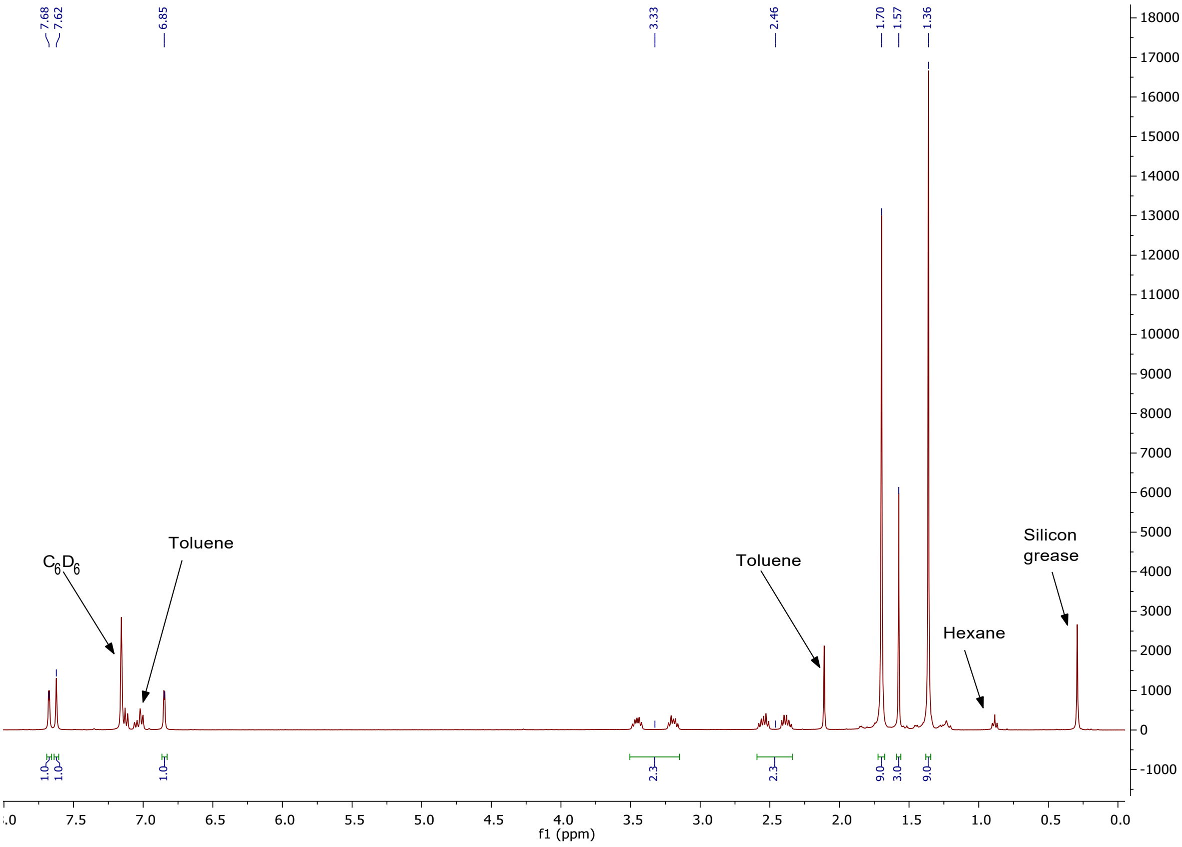

Figure SI19 ^{1}H NMR (400 MHz, C_6D_6) of Zn(**1**)$_2$.

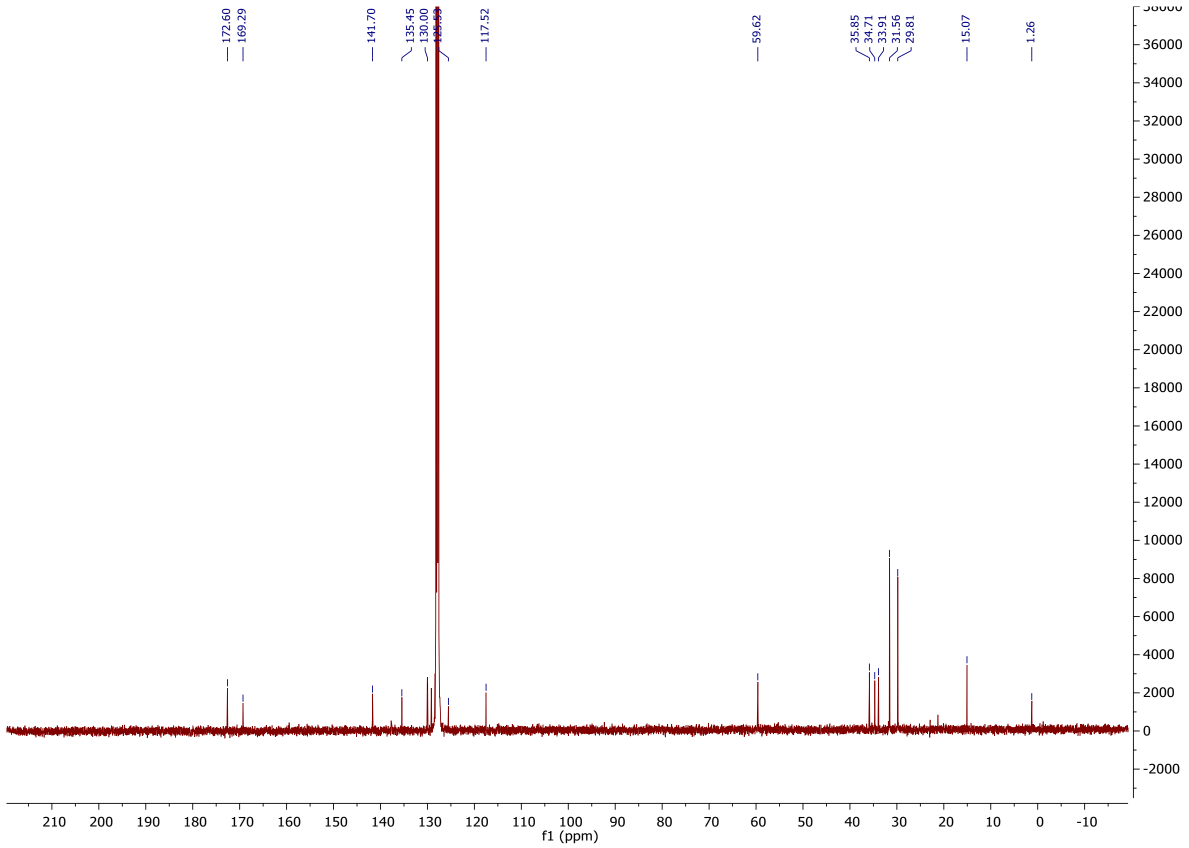

Figure SI20 $^{13}C\{^1H\}$ NMR (101 MHz, C_6D6 of Zn(**1**)$_2$.

Zn(**2**)$_2$ Ligand **2**H (2 mmol, 0.39 g) was dissolved in toluene (10 mL). $ZnEt_2$ (1 mmol, 1 mL, 1.0 M) was added dropwise and the solution stirred for two hours. The product crystallised from toluene as an off-white solid (0.37 g, 82%).

^1H NMR (500 MHz, $CDCl_3$) δ 8.12 (s, 1H, CH), 7.23 (ddd, *J* = 8.7, 7.0, 1.9 Hz, 1H, Ar), 7.05 (dd, *J* = 7.8, 1.9 Hz, 1H, Ar), 6.77 (d, *J* = 8.5 Hz, 1H, Ar), 6.53 (ddd, *J* = 8.0, 7.0, 1.2 Hz, 1H, Ar), 3.69 (t, *J* = 6.7 Hz, 2H, CH_2), 2.68 (t, *J* = 6.7 Hz, 2H, CH_2), 1.90 (s, 3H, CH_3).

$^{13}C\{^1H\}$ NMR (126 MHz, $CDCl_3$) δ 171.9 (CH), 170.7, 135.8, 135.2, 123.2, 117.9, 114.6 (Ar), 59.5, 34.7 (CH_2), 15.5 (S-CH_3).

Elemental analysis ($C_{20}H_{24}N_2O_2S_2Zn$) Calcd in %: C, 52.92; H, 5.33; N, 6.17. Found: C, 53.12; H, 5.33; N, 6.17.

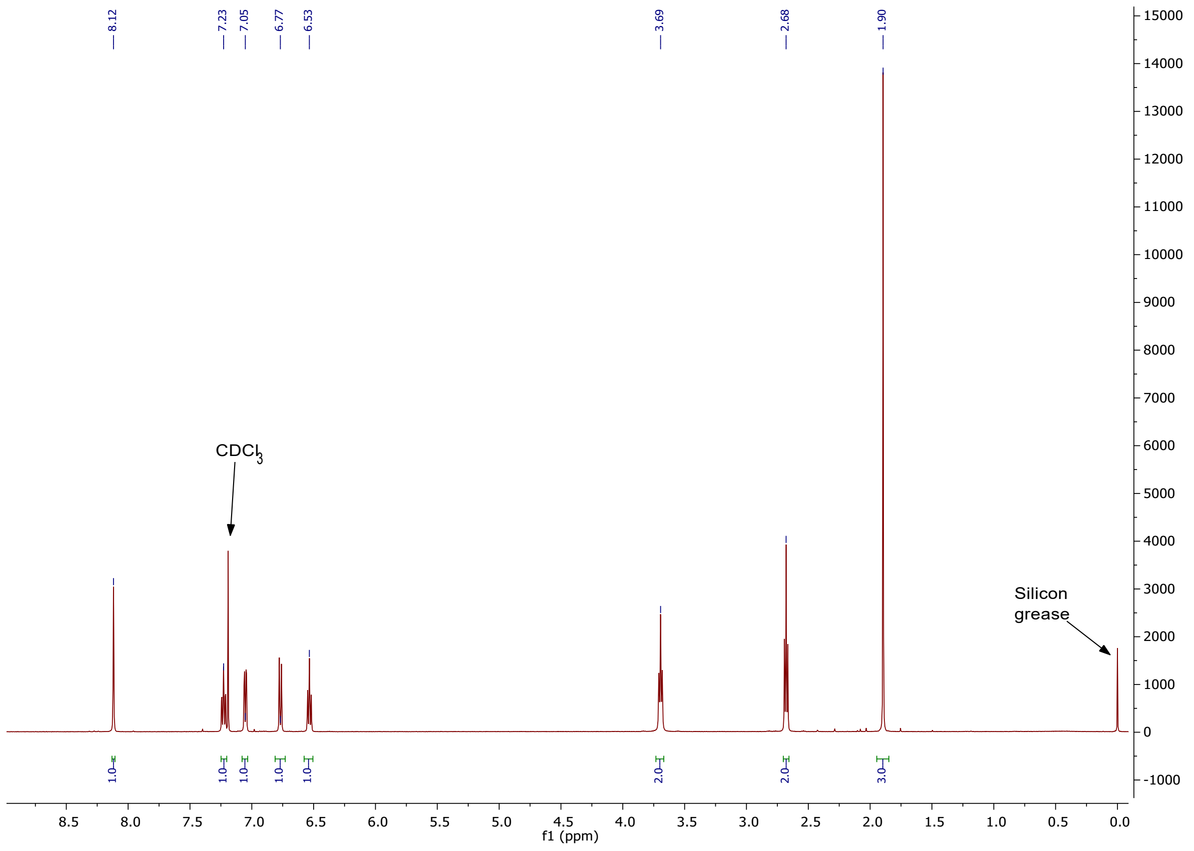

Figure SI21 ^{1}H NMR (500 MHz, $CDCl_3$) of Zn(**2**)$_2$.

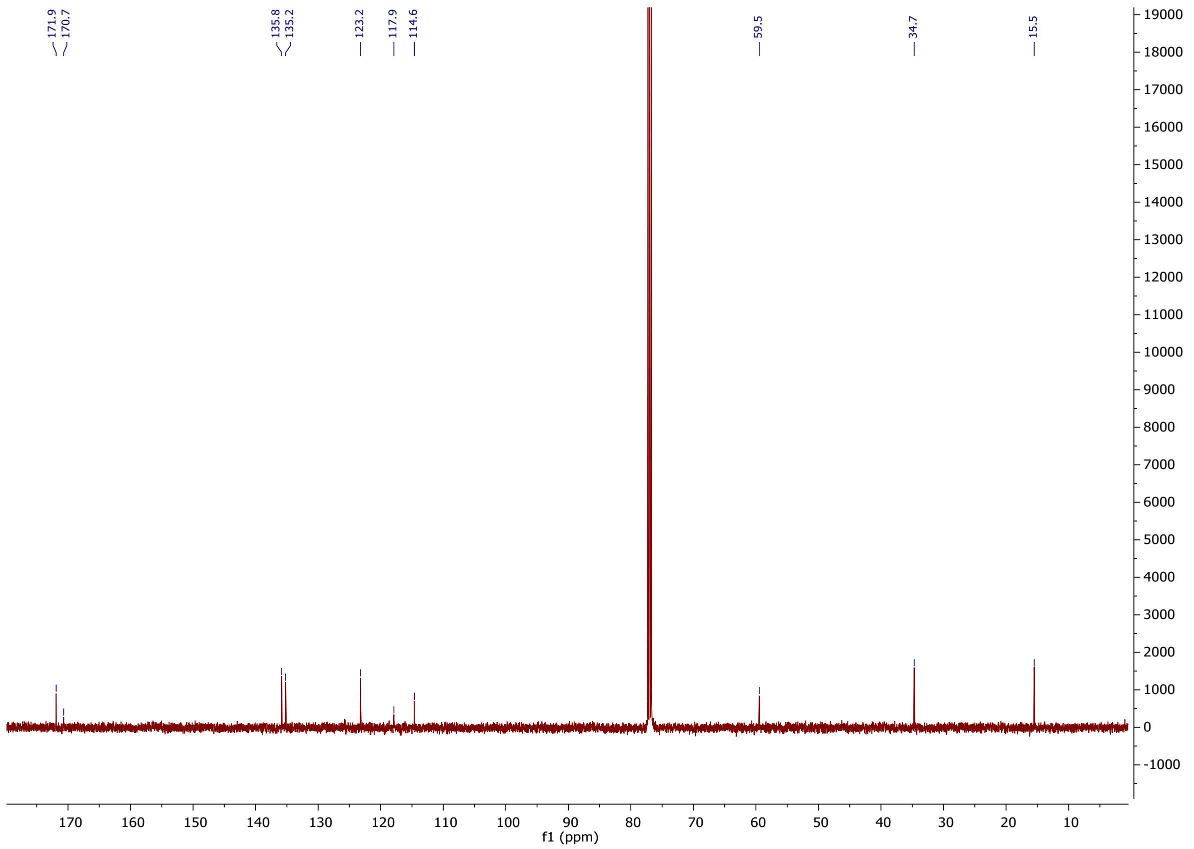

Figure SI22 $^{13}C\{^{1}H\}$ NMR (126 MHz, $CDCl_3$) of Zn(**2**)$_2$.

Zn(**3**)$_2$ Ligand **3**H (2 mmol, 0.53 g) was dissolved in toluene (10 mL). $ZnEt_2$ (1 mmol, 1 mL, 1.0 M) was added dropwise and the solution stirred for two hours. The product crystallised from toluene as a bright yellow solid (0.44 g, 74%).

^{1}H NMR (500 MHz, $CDCl_3$) δ 8.08 (s, 1H, CH), 7.36 (d, *J* = 2.8 Hz, 1H, Ar), 6.97 (d, *J* = 2.8 Hz, 1H, Ar), 3.81 (t, *J* = 6.0 Hz, 2H, CH_2), 2.71 (t, *J* = 6.0 Hz, 2H, CH_2), 1.83 (s, 3H, CH_3).

^{13}C{^{1}H} NMR (126 MHz, $CDCl_3$) δ 169.8 (CH), 164.1, 133.9, 132.7, 127.7, 118.8, 117.3 (Ar), 58.4, 35.1 (CH_2), 15.2 (S-CH_3).

Elemental analysis ($C_{20}H_{20}N_2O_2S_2Cl_4Zn$) Calcd in %: C, 40.60; H, 3.41; N, 4.73. Found: C, 40.57; H, 3.41; N, 4.64.

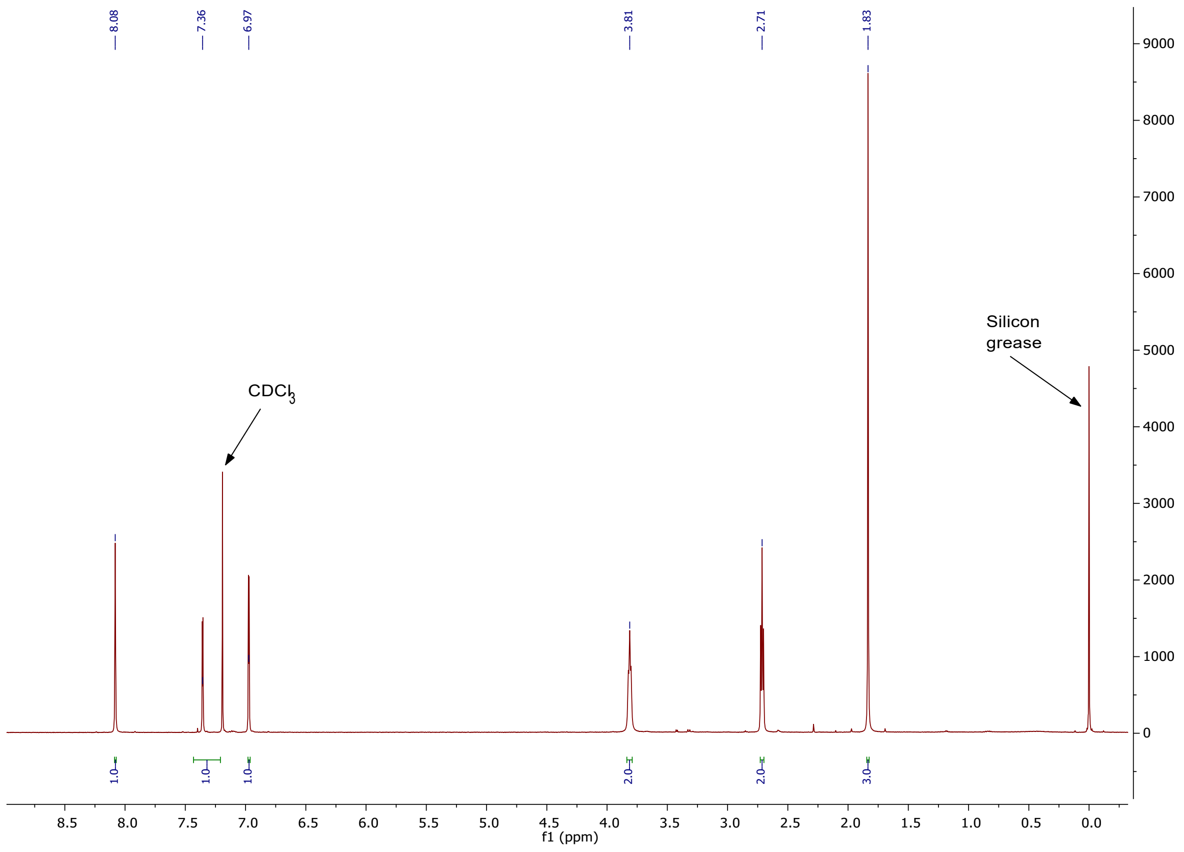

Figure SI23 ^{1}H NMR (500 MHz, $CDCl_3$) of Zn(**3**)$_2$.

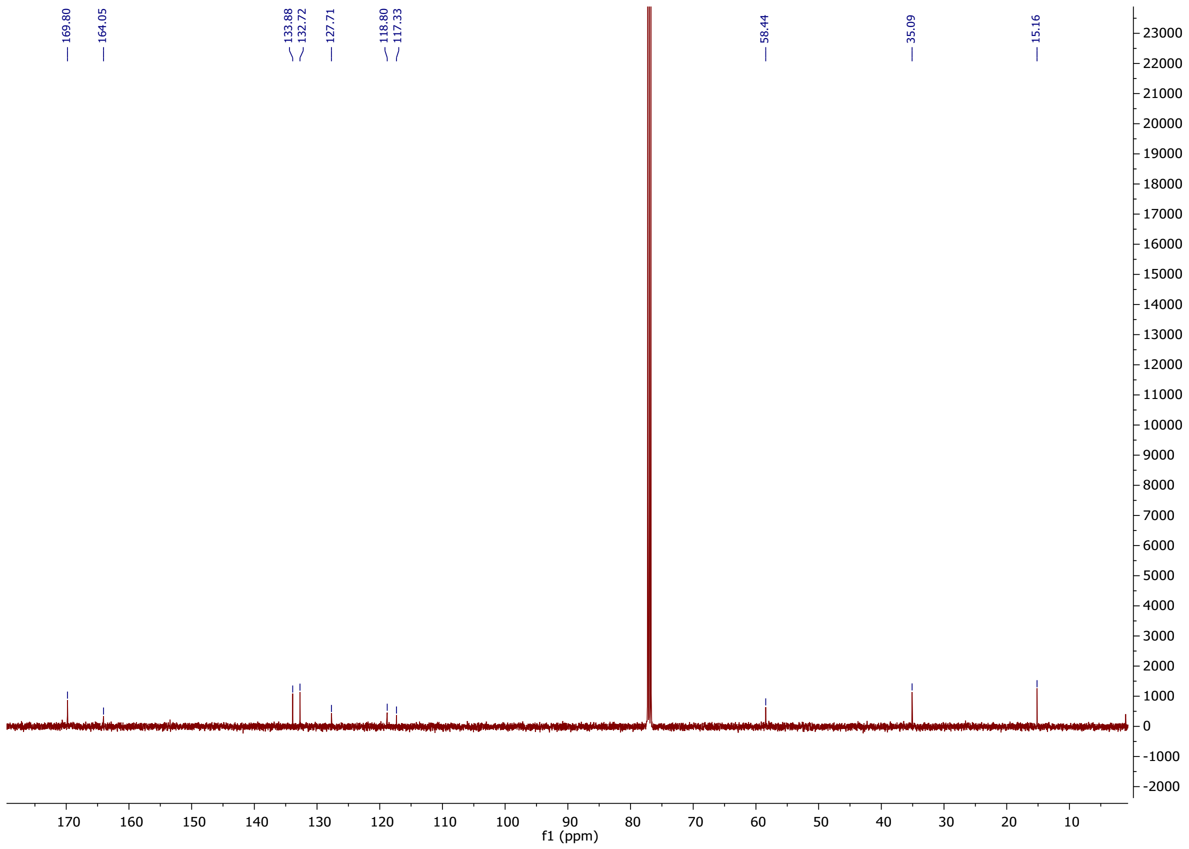

Figure SI24 ^{13}C NMR (126 MHz, $CDCl_3$) of Zn(**3**)$_2$.

Zn(**4**)$_2$ Ligand **4**H (2 mmol, 0.71 g) was dissolved in toluene (10 mL). $ZnEt_2$ (1 mmol, 1 mL, 1.0 M) was added dropwise and the solution stirred for two hours. The product crystallised from a mixture of toluene and hexane as a yellow solid (0.31 g, 40%).

^{1}H NMR (400 MHz, $CDCl_3$) δ 8.15 (s, 1H, CH), 7.44 (d, *J* = 2.6 Hz, 1H, Ar), 7.20 – 7.04 (m, 2H, Ar), 7.03 – 6.91 (m, 1H, Ar), 6.90 (d, *J* = 2.6 Hz, 1H, Ar), 6.53 (d, *J* = 7.8 Hz, 1H, Ar), 2.04 (s, 3H, CH_3), 1.38 (s, 9H, $C(CH_3)_3$), 1.30 (s, 9H, $C(CH_3)_3$).

$^{13}C\{^{1}H\}$ NMR (101 MHz, $CDCl_3$) δ 170.9 (CH), 170.8, 148.5, 142.1, 134.7, 131.8, 130.5, 129.4, 127.1, 126.5, 126.0, 121.9, 117.7 (Ar), 35.5, 33.8 (CH), 31.3, 29.3 (CH_3), 16.8 (S-CH_3).

Elemental analysis ($C_{44}H_{56}N_2O_2S_2Zn$) Calcd in %: C, 68.24; H, 7.29; N, 3.62. Found: C, 65.08; H, 7.25; N, 3.41.

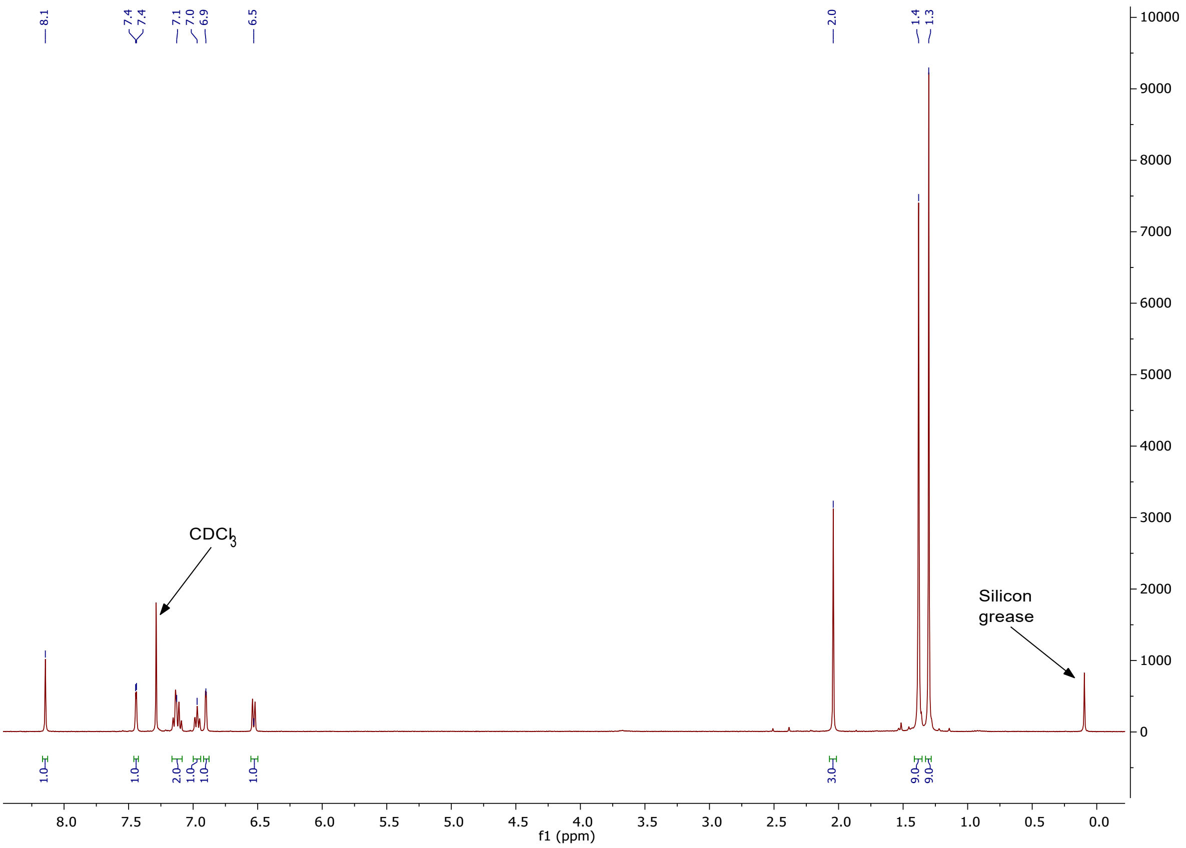

Figure SI25 ^{1}H NMR (400 MHz, $CDCl_3$) of Zn(**4**)$_2$.

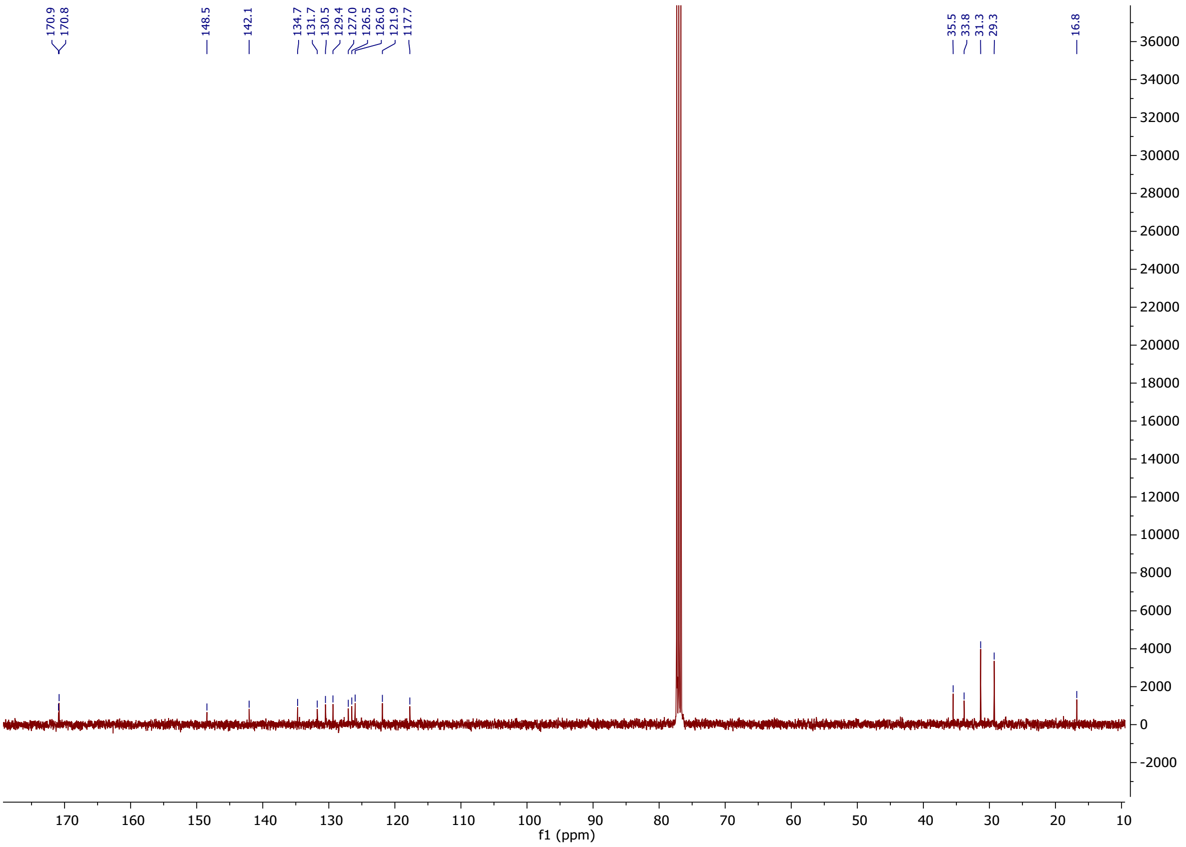

Figure SI26 ^{13}C{^{1}H} NMR (101 MHz, $CDCl_3$) of Zn(**4**)$_2$.

Zn(**5**)$_2$ Ligand **5**H (2 mmol, 0.48 g) was dissolved in toluene (10 mL). $ZnEt_2$ (1 mmol, 1 mL, 1.0 M) was added dropwise and the solution stirred for two hours. The product crystallised from a mixture of toluene and hexane as a pale-yellow solid (0.28 g, 50%).

^{1}H NMR (400 MHz, $CDCl_3$) δ 8.13 (s, 1H, CH), 7.34 (ddd, *J* = 8.8, 6.9, 2.0 Hz, 1H, Ar), 7.31 – 7.24 (m, 1H, Ar), 7.25 – 7.18 (m, 1H, Ar), 7.10 (dd, *J* = 7.9, 2.0 Hz, 1H, Ar), 7.04 – 6.90 (m, 2H, Ar), 6.66 – 6.53 (m, 1H, Ar), 6.48 (d, *J* = 7.6 Hz, 1H, Ar), 2.11 (s, 3H, CH_3).

^{13}C{^{1}H} NMR (101 MHz, $CDCl_3$) δ 172.0 (CH), 169.2, 146.6, 134.9, 130.4, 128.0, 127.2, 126.4, 125.8, 125.5, 123.1, 120.4, 117.8, 113.1 (Ar), 16.1 (S-CH_3). Signals corresponding to toluene are also present.

Elemental analysis ($C_{28}H_{24}N_2O_2S_2Zn$) Calcd in %: C, 61.15; H, 4.40; N, 5.09. Found: C, 61.67; H, 4.73; N, 4.75.

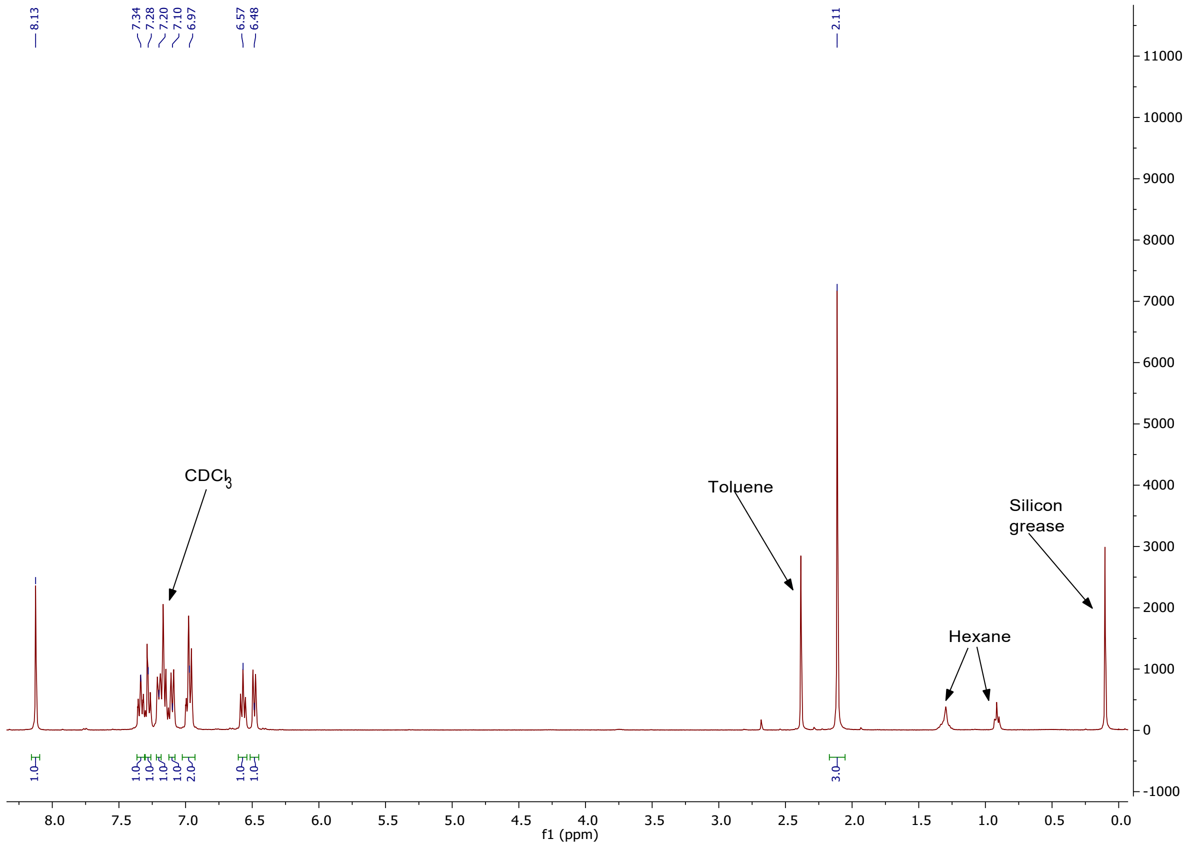

Figure SI27 ^{1}H NMR (400 MHz, $CDCl_3$) of Zn(**5**)$_2$.

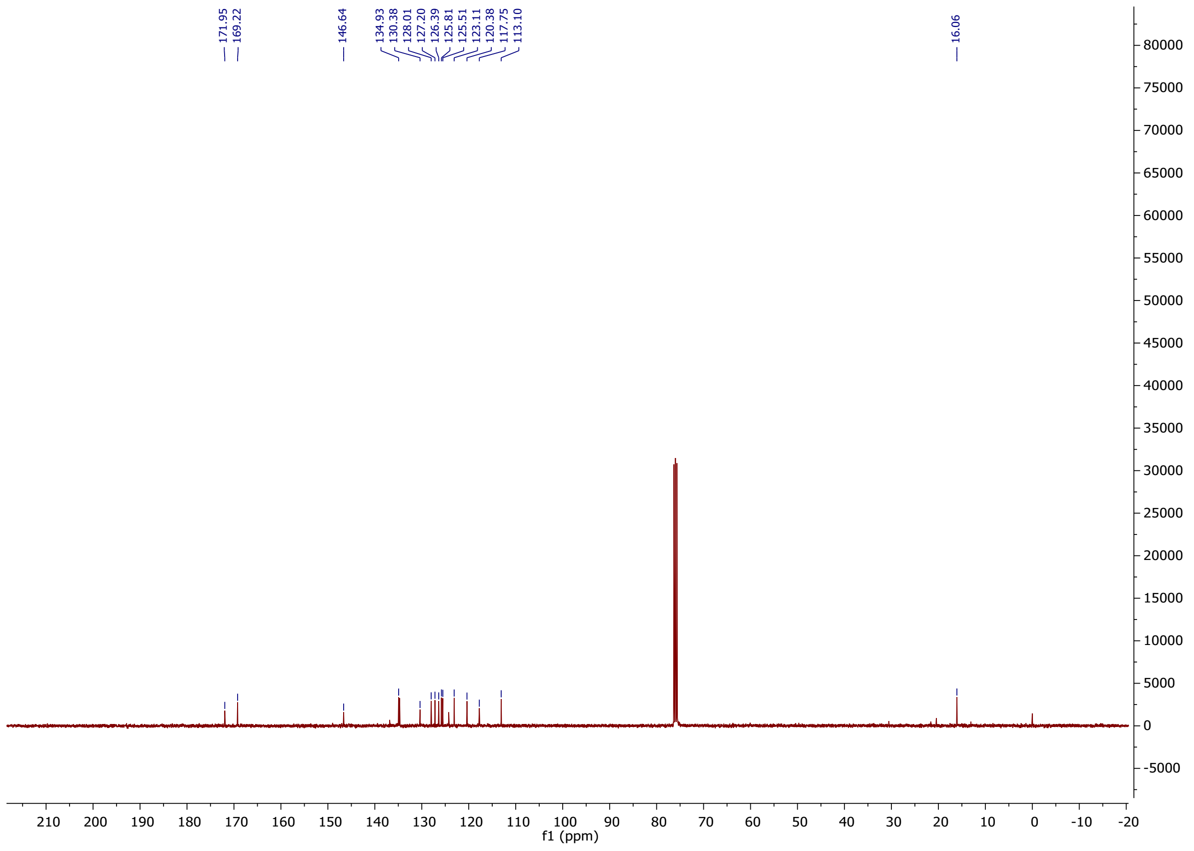

Figure SI28 $^{13}C\{^{1}H\}$ NMR (101 MHz, $CDCl_3$) of $Zn(5)_2$.

$Zn(\mathbf{6})_2$ Ligand **6**H (2 mmol, 0.62 g) was dissolved in toluene (10 mL). $ZnEt_2$ (1 mmol, 1 mL, 1.0 M) was added dropwise and the solution stirred for two hours. The product crystallised from a mixture of toluene and hexane as a yellow solid (0.55 g, 80%).

^{1}H NMR (400 MHz, $CDCl_3$) δ 8.20 (s, 1H, CH), 7.42 (d, J = 2.7 Hz, 1H, Ar), 7.37 – 7.25 (m, 3H, Ar), 7.07 (d, J = 2.8 Hz, 1H, Ar), 6.92 (d, J = 8.0 Hz, 1H, Ar), 2.13 (s, 3H, CH_3).

$^{13}C\{^{1}H\}$ NMR (101 MHz, $CDCl_3$) δ 167.3 (CH), 164.5, 145.9, 133.4, 131.6, 127.3, 127.2, 126.8, 126.6, 124.3, 120.7, 118.5, 116.1 (Ar), 16.4 (S-CH_3). Signals corresponding to toluene are also present.

Elemental analysis ($C_{28}H_{20}N_2O_2S_2Cl_4Zn$ + C_7H_8) Calcd in %: C, 53.88; H, 3.61; N, 3.59. Found: C, 52.22; H, 3.60; N, 3.54.

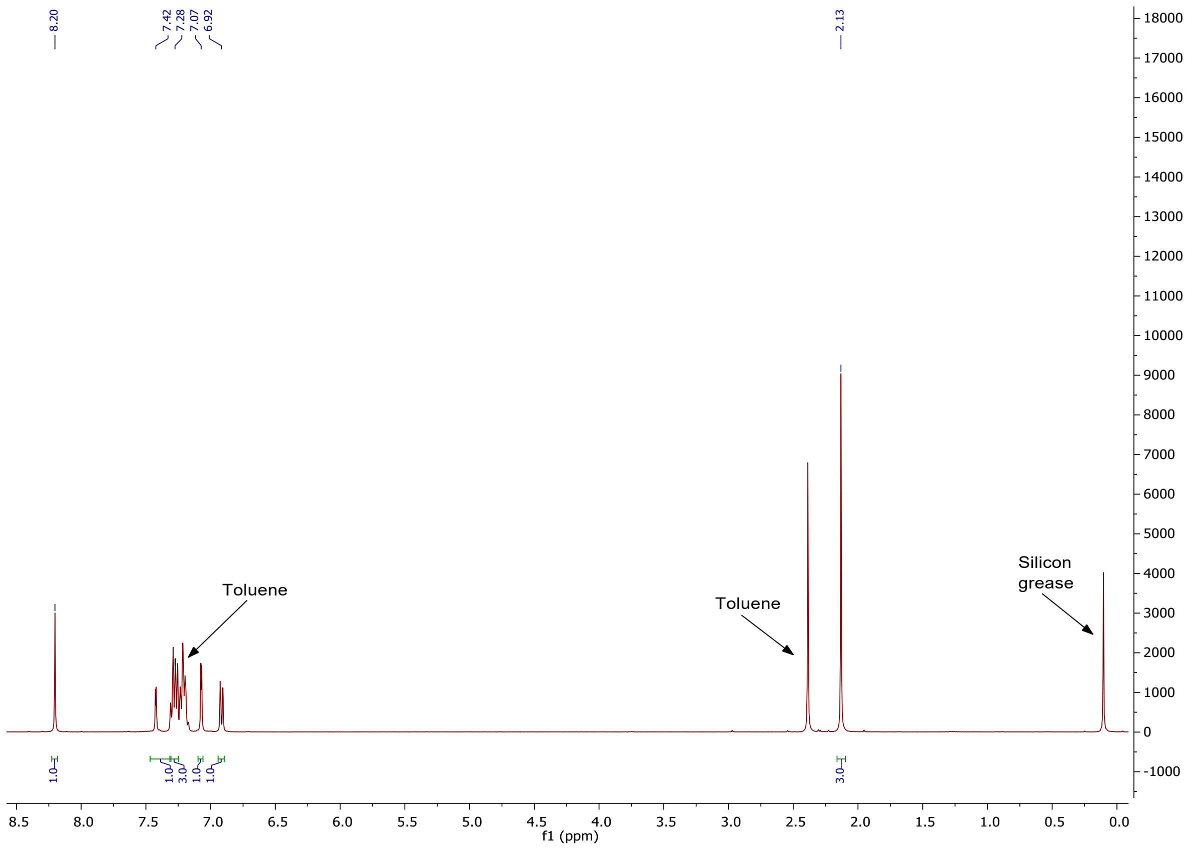

Figure SI29 ^{1}H NMR (400 MHz, $CDCl_3$) of Zn(**6**)$_2$.

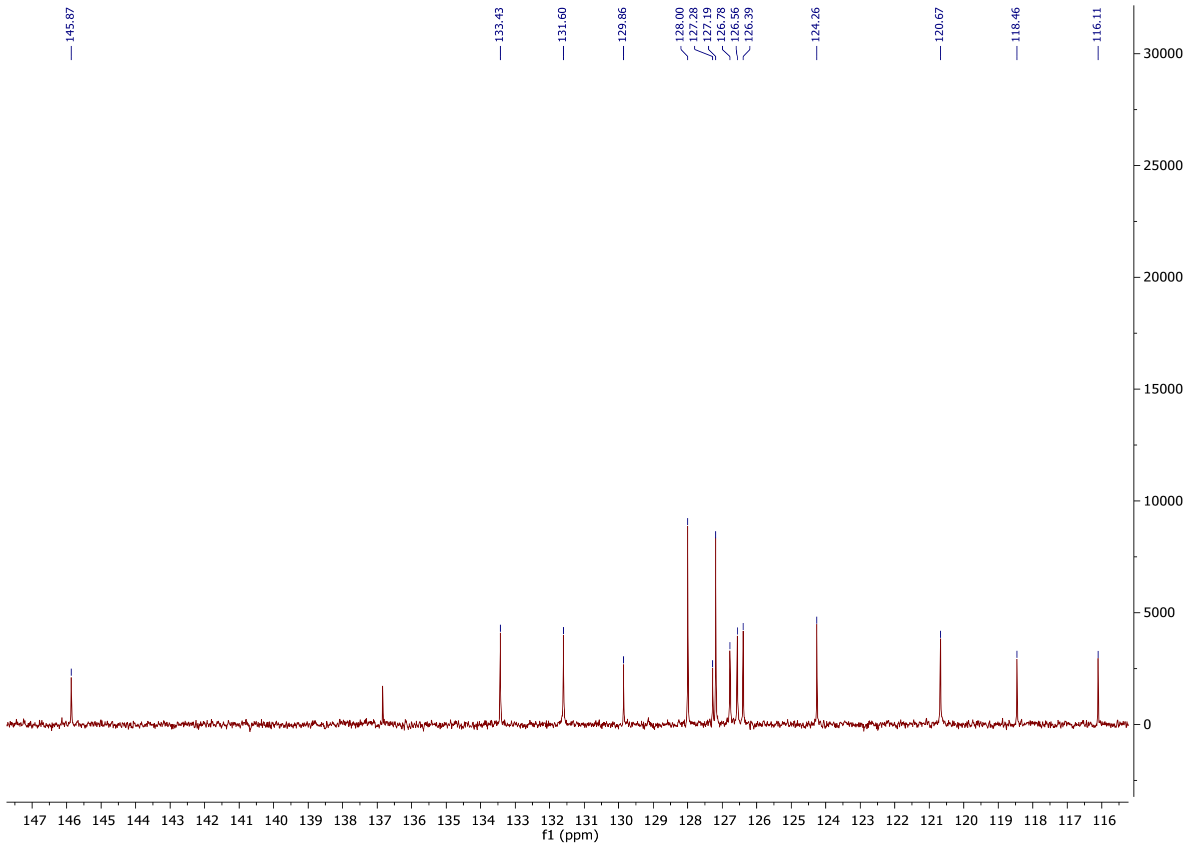

Figure SI30 $^{13}C\{^{1}H\}$ NMR (101 MHz, $CDCl_3$) of Zn(**6**)$_2$.

Zn(**7**)$_2$ Ligand **7**H (2 mmol, 0.82 g) was dissolved in toluene (10 mL). $ZnEt_2$ (1 mmol, 1 mL, 1.0 M) was added dropwise and the solution stirred for two hours. The product crystallised from hexane as a yellow solid (0.21 g, 24%).

^{1}H NMR (500 MHz, $CDCl_3$) δ 8.01 (s, 1H, CH), 7.51 (d, *J* = 7.0 Hz, 1H, Ar), 7.46 (d, *J* = 2.7 Hz, 1H, Ar), 7.12 (m, 2H, Ar), 6.82 (d, *J* = 2.6 Hz, 1H, Ar), 6.53 (dd, *J* = 7.2, 2.1 Hz, 1H, Ar), 1.36 (s, 9H, C(CH_3)), 1.23 (s, 9H, C(CH_3)).

^{13}C{^{1}H} NMR (101 MHz, $CDCl_3$) δ 173.4 (CH), 170.7, 153.5, 142.5, 137.9, 135.7, 132.5, 131.8, 129.9, 126.6, 124.2, 117.0, 110.9 (Ar), 35.5, 33.9 (CH), 31.2, 29.4 (CH_3).

Elemental analysis ($C_{44}H_{50}N_2O_2S_2F_6Zn$) Calcd in %: C, 59.89; H, 5.71; N, 3.17. Found: C, 58.71; H, 5.84; N, 3.08.

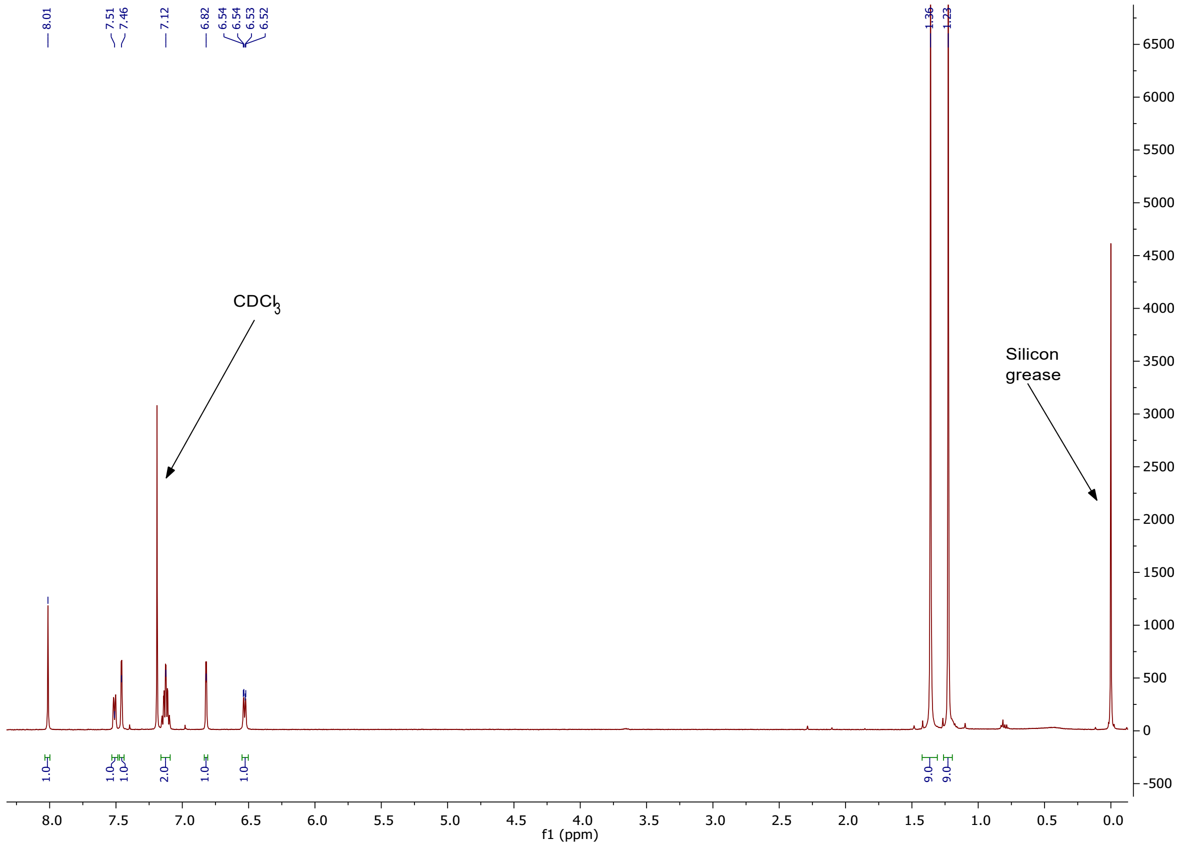

Figure SI31 ^{1}H NMR (500 MHz, $CDCl_3$) of Zn(**7**)$_2$.

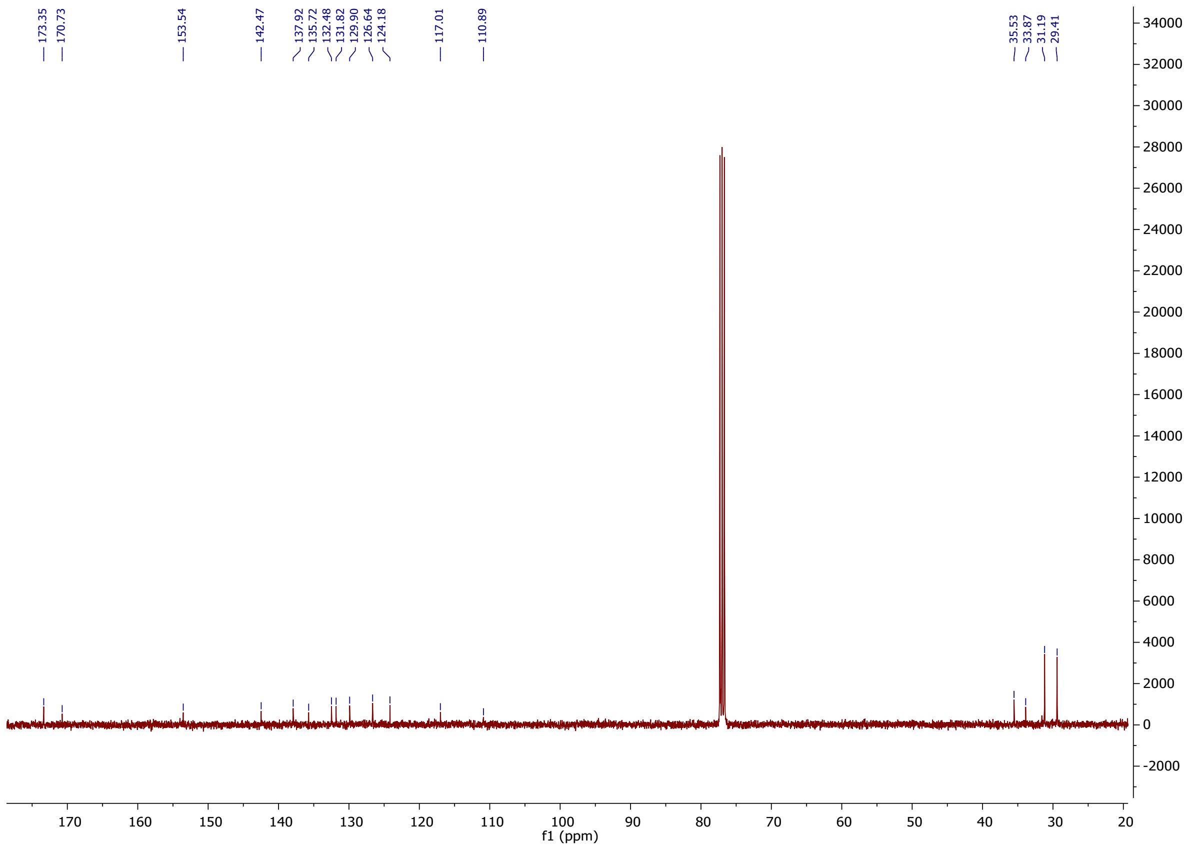

Figure SI32 $^{13}C\{^1H\}$ NMR (101 MHz, $CDCl_3$) of $Zn(7)_2$.

$Zn(\mathbf{8})_2$ Ligand **8**H (2 mmol, 0.59 g) was dissolved in toluene (10 mL). $ZnEt_2$ (1 mmol, 1 mL, 1.0 M) was added dropwise and the solution stirred for two hours. The product crystallised from a mixture of toluene and hexane as a yellow solid (0.33 g, 50%).

^1H NMR (400 MHz, $CDCl_3$) δ 8.11 (s, 1H, CH), 7.66 (d, *J* = 7.7 Hz, 1H, Ar), 7.40 (ddd, *J* = 8.9, 6.9, 1.9 Hz, 1H, Ar), 7.38 – 7.33 (m, 1H, Ar), 7.31 – 7.25 (m, 1H, Ar), 7.12 (dd, *J* = 8.0, 1.8 Hz, 1H, Ar), 6.95 (d, *J* = 8.7 Hz, 1H, Ar), 6.90 – 6.78 (m, 1H, Ar), 6.64 (ddd, *J* = 7.8, 6.9, 1.1 Hz, 1H, Ar).

$^{13}C\{^1H\}$ NMR (101 MHz, $CDCl_3$) δ 173.1 (CH), 172.3, 152.6, 138.1, 136.8, 136.6, 132.6, 127.3, 124.1, 124.0, 118.0, 115.5, 115.1 (Ar).

Elemental analysis ($C_{28}H_{18}N_2O_2S_2F_6Zn$) Calcd in %: C, 51.11; H, 2.76; N, 4.26. Found: C, 50.36; H, 2.99; N, 4.10.

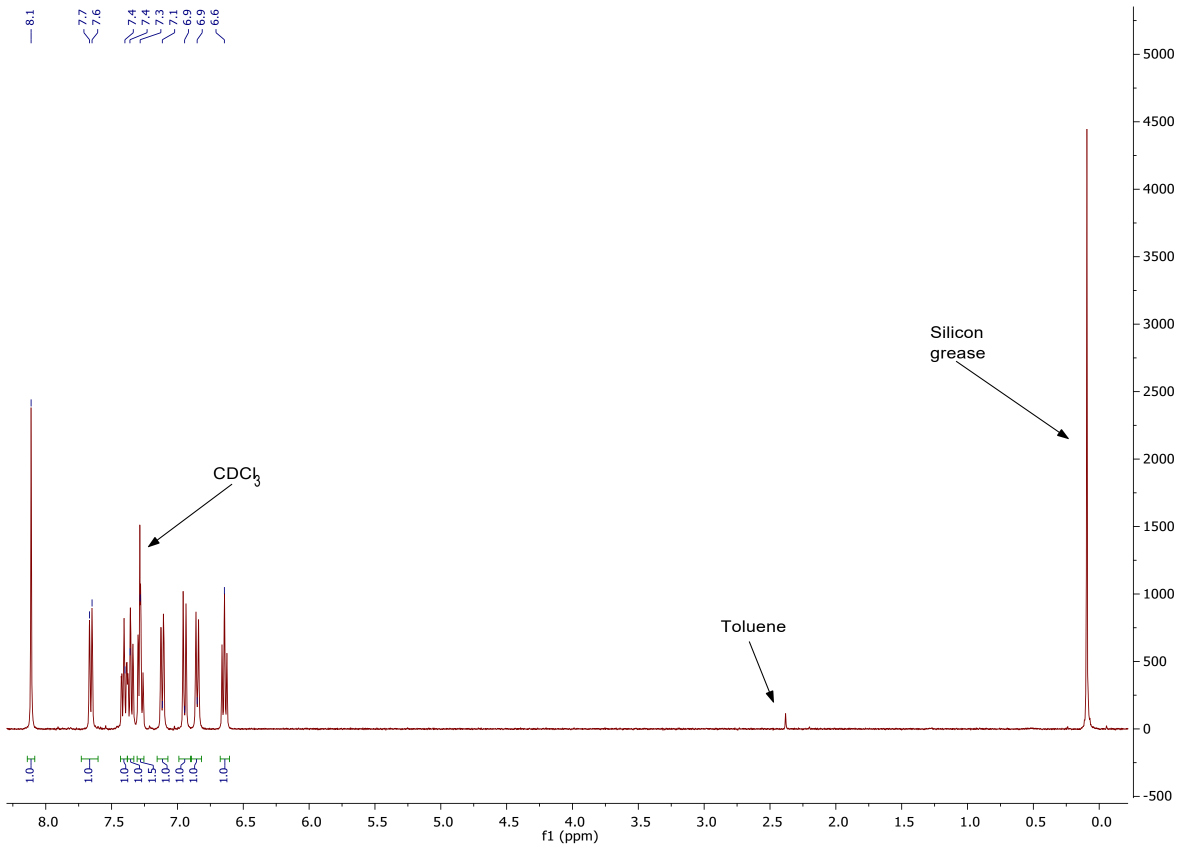

Figure SI33 ^1H NMR (400 MHz, $CDCl_3$) of Zn(**9**)$_2$.

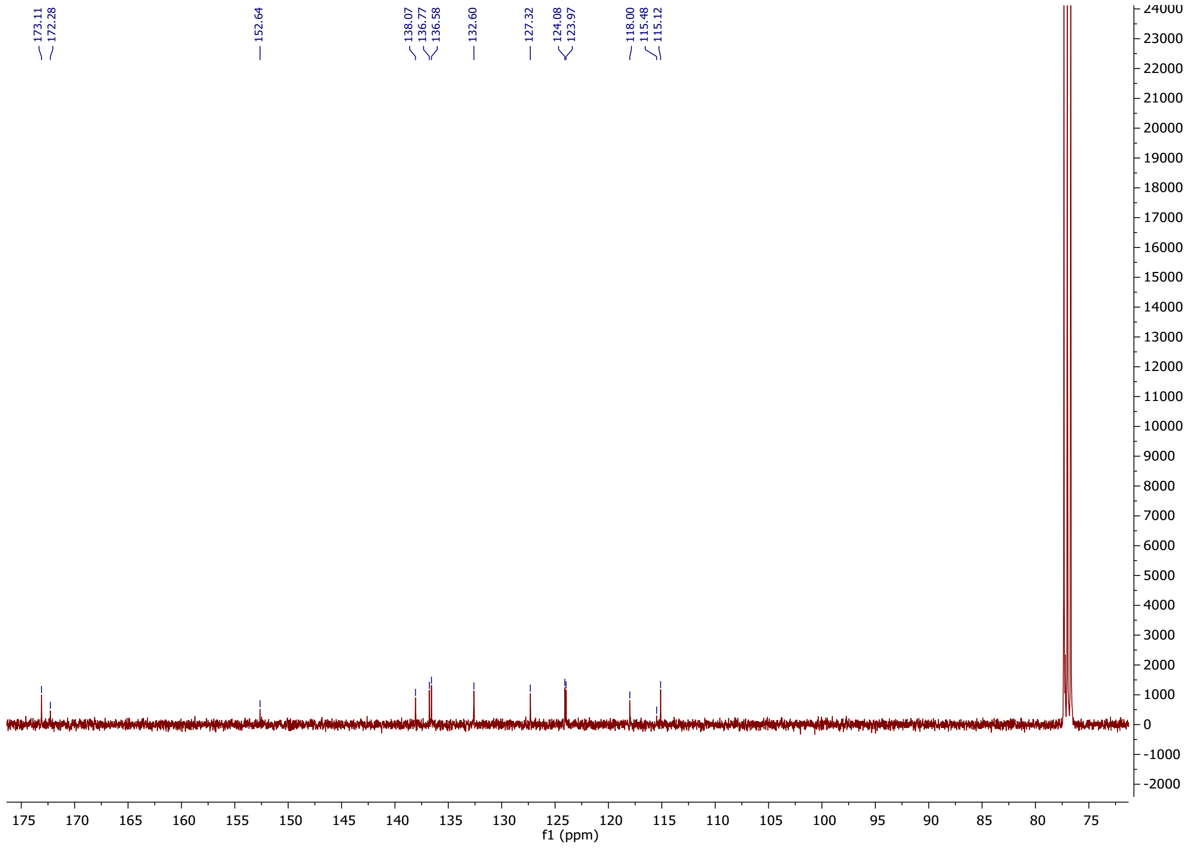

Figure SI34 $^{13}C\{^1H\}$ NMR (101 MHz, $CDCl_3$) of Zn(**8**)$_2$.

Zn(**9**)$_2$ Ligand **9**H (2 mmol, 0.73 g) was dissolved in toluene (10 mL). $ZnEt_2$ (1 mmol, 1 mL, 1.0 M) was added dropwise and the solution stirred for two hours. The product crystallised from a mixture of toluene and hexane as a yellow solid (0.54 g, 68%).

^{1}H NMR (400 MHz, $CDCl_3$) δ 8.10 (s, 1H, CH), 7.68 (d, *J* = 7.8 Hz, 1H, Ar), 7.47 (t, *J* = 7.7 Hz, 1H, Ar), 7.43 – 7.39 (m, 1H, Ar), 7.33 (t, *J* = 7.7 Hz, 1H, Ar), 7.11 (d, *J* = 7.9 Hz, 1H, Ar), 7.04 (d, *J* = 2.7 Hz, 1H, Ar).

^{13}C{^{1}H} NMR (101 MHz, $CDCl_3$) δ 171.7 (CH), 138.5, 135.2, 133.0, 132.8, 130.5, 129.0, 128.6, 128.2, 128.1, 125.9, 124.0, 118.7 (Ar).

Elemental analysis ($C_{28}H_{14}N_2O_2S_2F_6Cl_4Zn$) Calcd in %: C, 42.26; H, 1.77; N, 3.52. Found: C, 44.22; H, 2.27; N, 3.32.

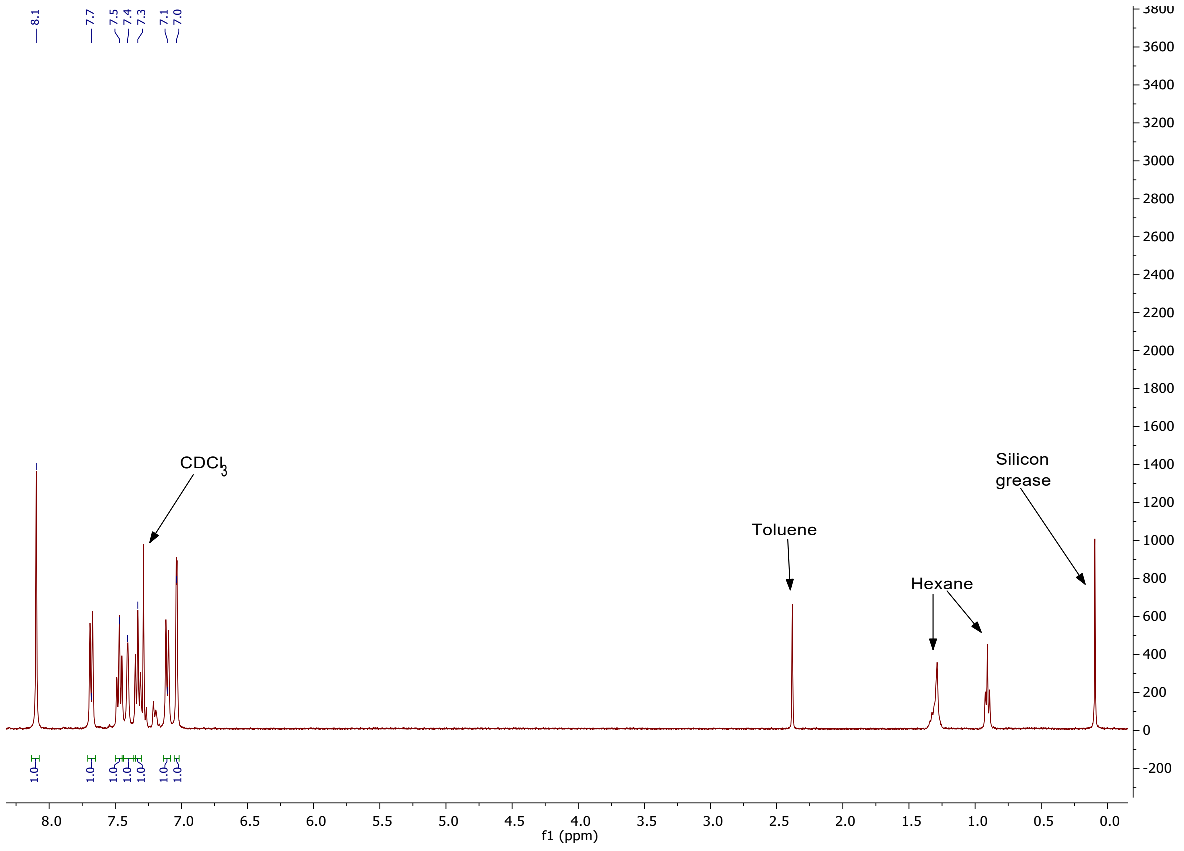

Figure SI35 ^{1}H NMR (400 MHz, $CDCl_3$) of Zn(**9**)$_2$.

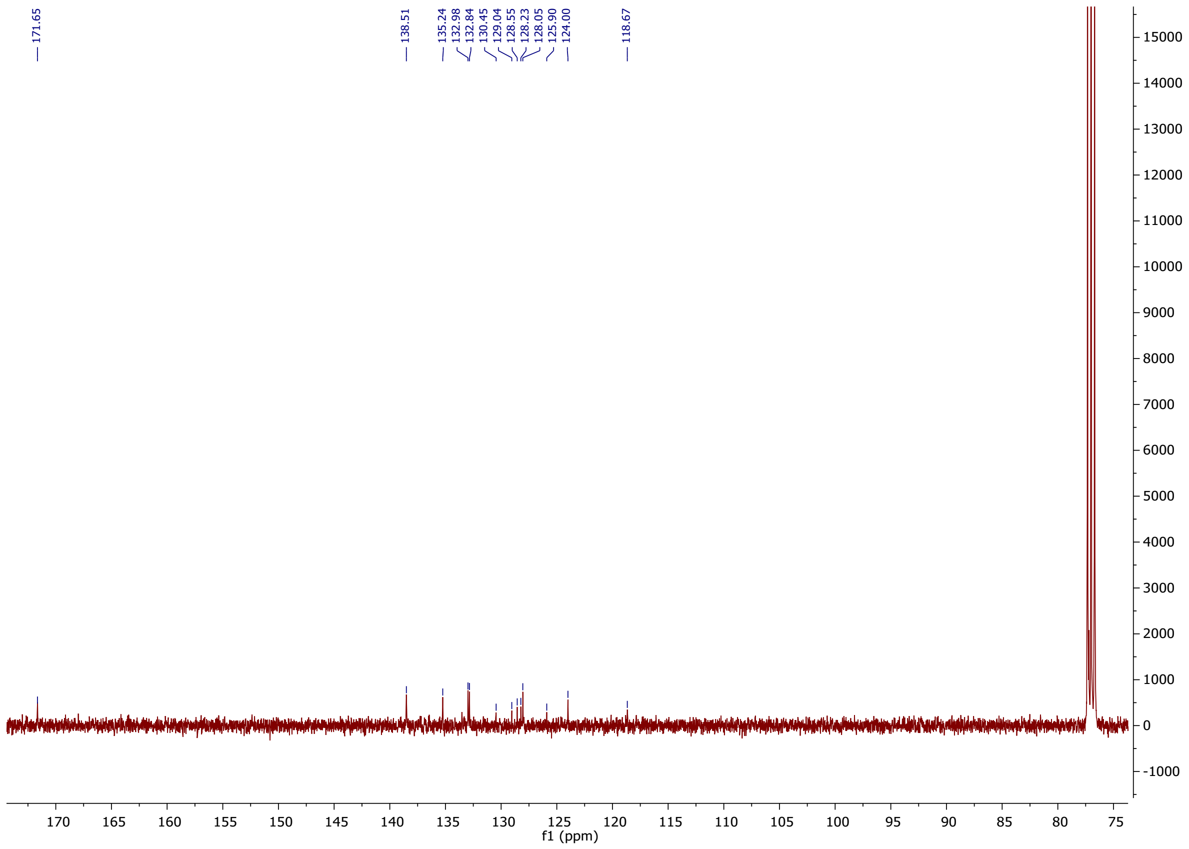

Figure SI36 $^{13}C\{^{1}H\}$ NMR (101 MHz, $CDCl_3$) of Zn(**9**)$_2$.

DOSY NMR

The equations below can be used to estimate the van der Waals radii of the samples, using only the ratio of the diffusion rates and an estimated radius for the solvent. The complex formed from **9**H is dimeric in the solid state so comparison with monomeric complexes should give an indication as to its nature in solution.

Table SI1 Diffusion coefficients D_t (10^{-9} m^2 s^{-1}) for the solvent (C_6D_6) for the three samples.

Sample	D_t^{so}	D_t^{sa}	Ratio (D_t^{so} / D_t^{sa})
Zn(**4**)$_2$	1.722	0.4731	3.64
Zn(**7**)$_2$	1.718	0.5590	3.07
Zn(**9**)$_2$	1.675	0.4710	3.56

$$\frac{D_t^{so}}{D_t^{sa}} = \frac{c^{so} r_H^{so}}{c^{sa} r_H^{sa}}$$

$$c = \frac{6}{\left[1 + 0.695\left(\frac{r_{solv}}{r_H}\right)^{2.234}\right]}$$

For the solvent, c^{so} = 4.16.

For benzene-d6, the rH is reported at 2.68 Å and will be similar.

This enables the calculation of $c^{sa}r_H^{sa}$, and this can then be determined.

$$c^{sa} r_H^{sa} = c^{so} r_H^{so} \times \frac{D_t^{so}}{D_t^{sa}}$$

Table SI2 r_H, c and V for the three samples.

Sample	$c_{sa}r_H^{sa}$	rH / Å	c	V / Å^3
Zn(**4**)$_2$	40.58	7.25	5.58	1,596
Zn(**7**)$_2$	34.26	6.30	5.44	1,047
Zn(**9**)$_2$	39.69	7.13	5.57	1,518

The data suggest that Zn(**4**,**7**,**9**)$_2$ are of similar size and are likely to be monomeric in solution.

Polymer characterisation

Homonuclear decoupled spectra examples

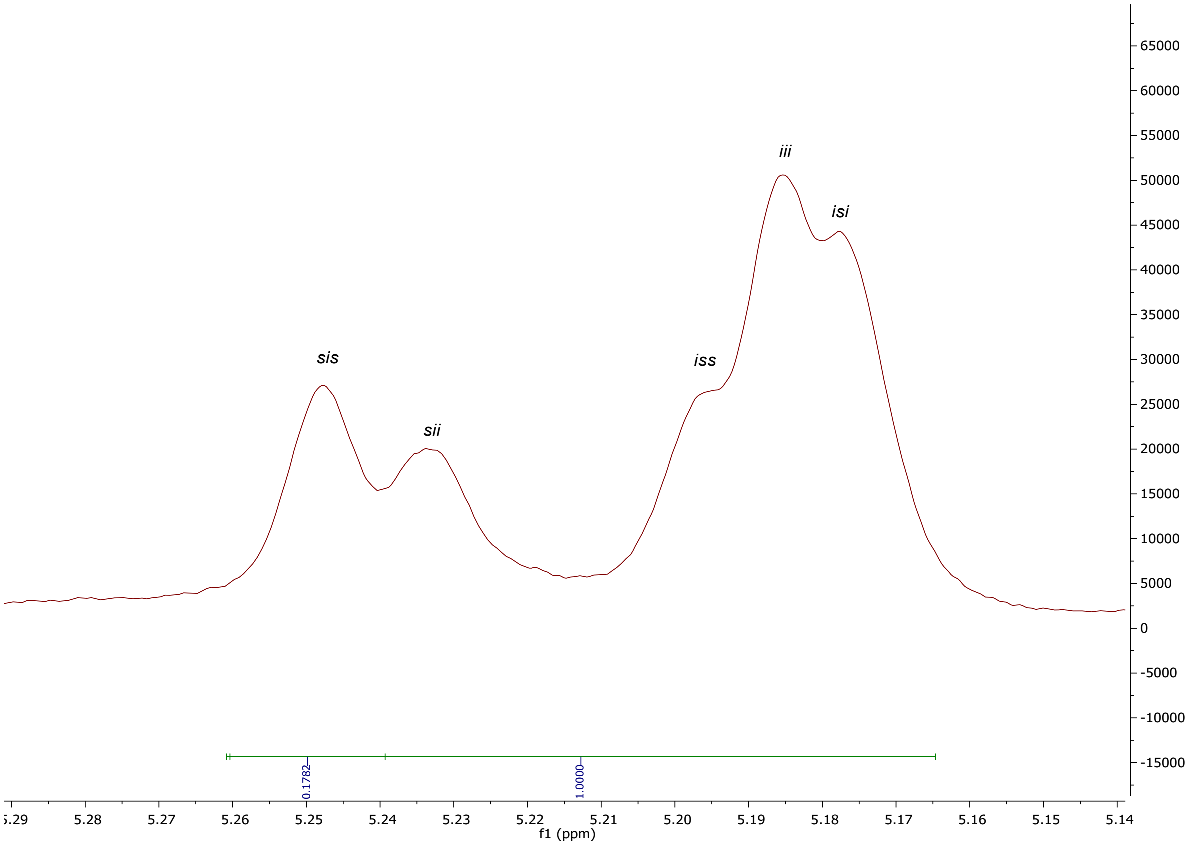

Figure SI37 $^{1}H\{^{1}H\}$ NMR spectrum of atactic PLA from $Zn(6)_2$. P_m = 0.61.

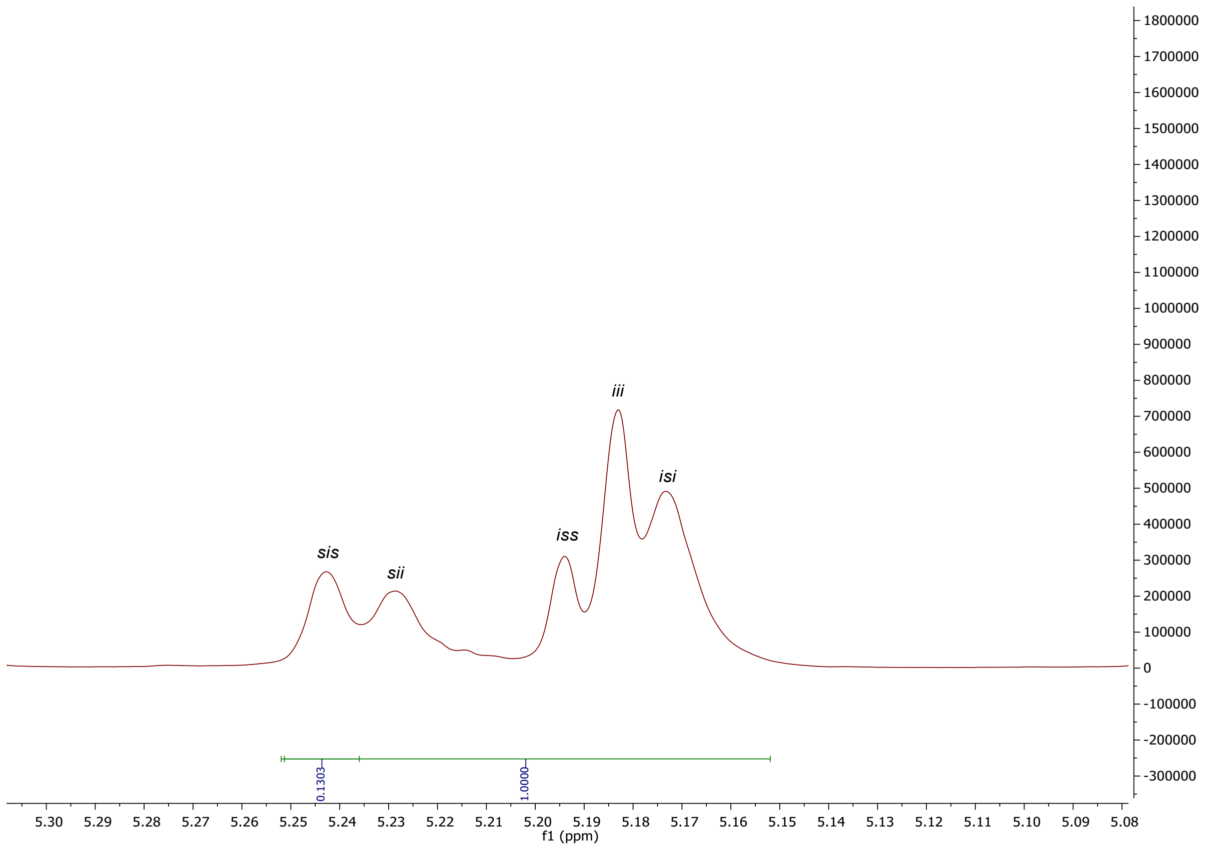

Figure SI38 $^{1}H\{^{1}H\}$ NMR spectrum of atactic PLA from $Zn(8)_2$. $P_m = 0.51$.

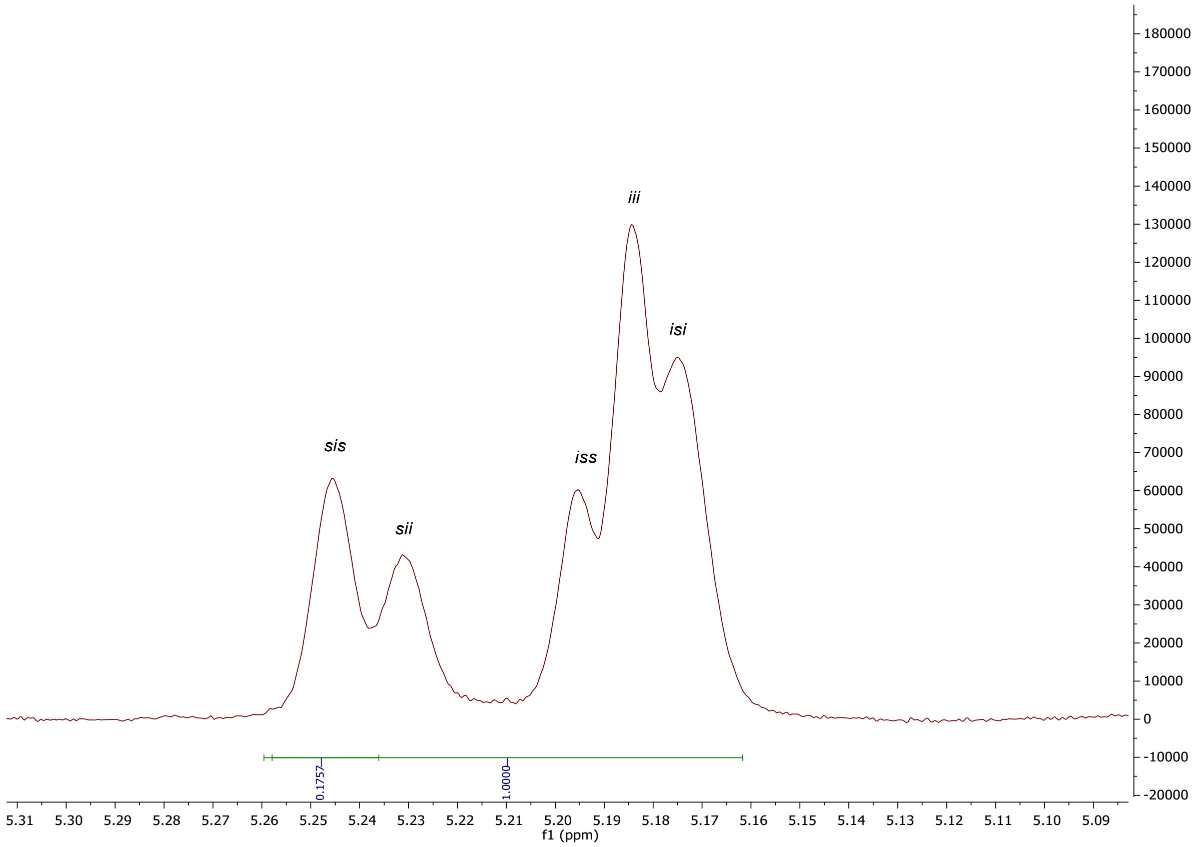

Figure SI39 $^{1}H\{^{1}H\}$ NMR spectrum of atactic PLA from $Zn(9)_2$. $P_m = 0.60$.

GPC

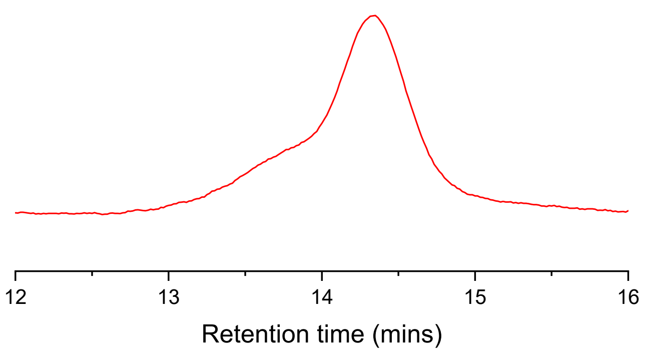

Figure SI40 GPC trace of PLA initiated by Zn(**2**)$_2$ (130 °C, 30 mins) at a ratio of 3000:1:10 ([LA]:[I]:[BnOH]) in the melt. M_n (GPC) = 19200 gmol^{-1}, Đ = 1.14, M_n (theo.) = 31650 gmol^{-1}.

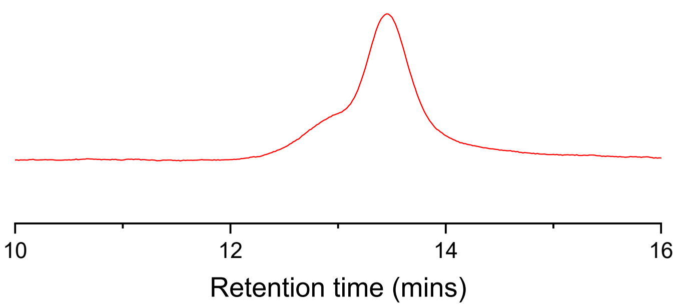

Figure SI41 GPC trace of PLA initiated by Zn(**6**)$_2$ (180 °C, 40 mins) at a ratio of 3000:1:10 ([LA]:[I]:[BnOH]) in the melt. M_n (GPC) = 38400 gmol^{-1}, Đ = 1.16, M_n (theo.) = 23050 gmol^{-1}.

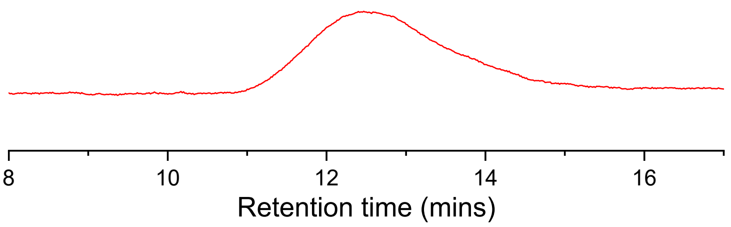

Figure SI42 GPC trace of PLA initiated by Zn(**9**)$_2$ (180 °C, 12 mins) at a ratio of 10000:1:30 ([LA]:[I]:[BnOH]) in the melt. M_n (GPC) = 42050 gmol^{-1}, Đ = 1.68, M_n (theo.) = 31400 gmol^{-1}.

MALDI-ToF

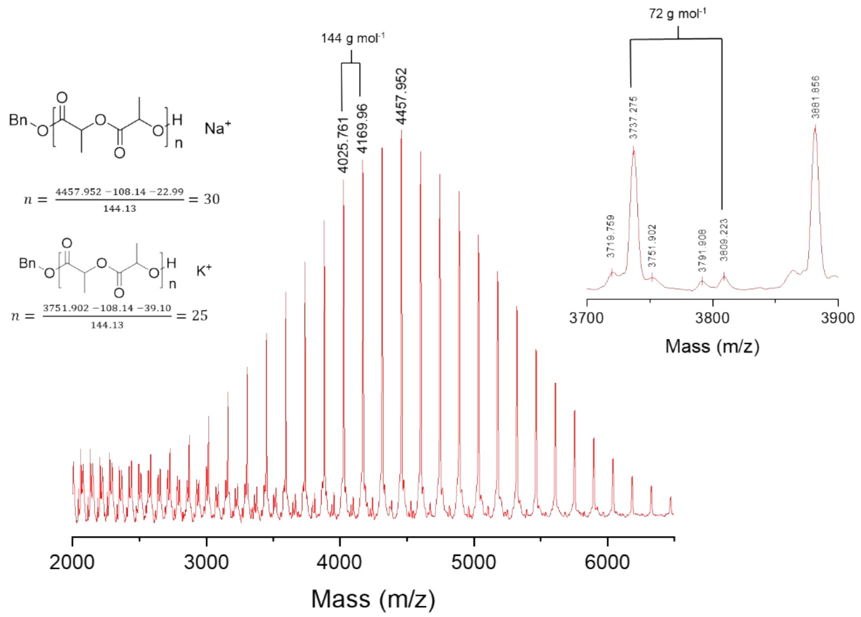

Figure SI43 MALDI – ToF spectrum of PLA derived from Zn(**3**)$_2$ (130 °C, 60 minutes) at a ratio of 3000:1:10 ([LA]:[I]:[BnOH]) solvent free. $M_{n\ GPC}$ = 8150 gmol^{-1}, M_n(Theo.) = 5850 gmol^{-1}. Main series is linear polymer with BnO + H end groups. Series ionised by potassium and transesterified polymer also present.

Figure SI44 White PLLA produced from Zn(**7**)$_2$ at [LA]:[Zn]:[BnOH] = 10000:1:30.

DSC

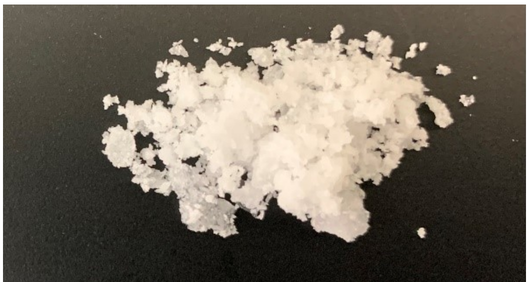

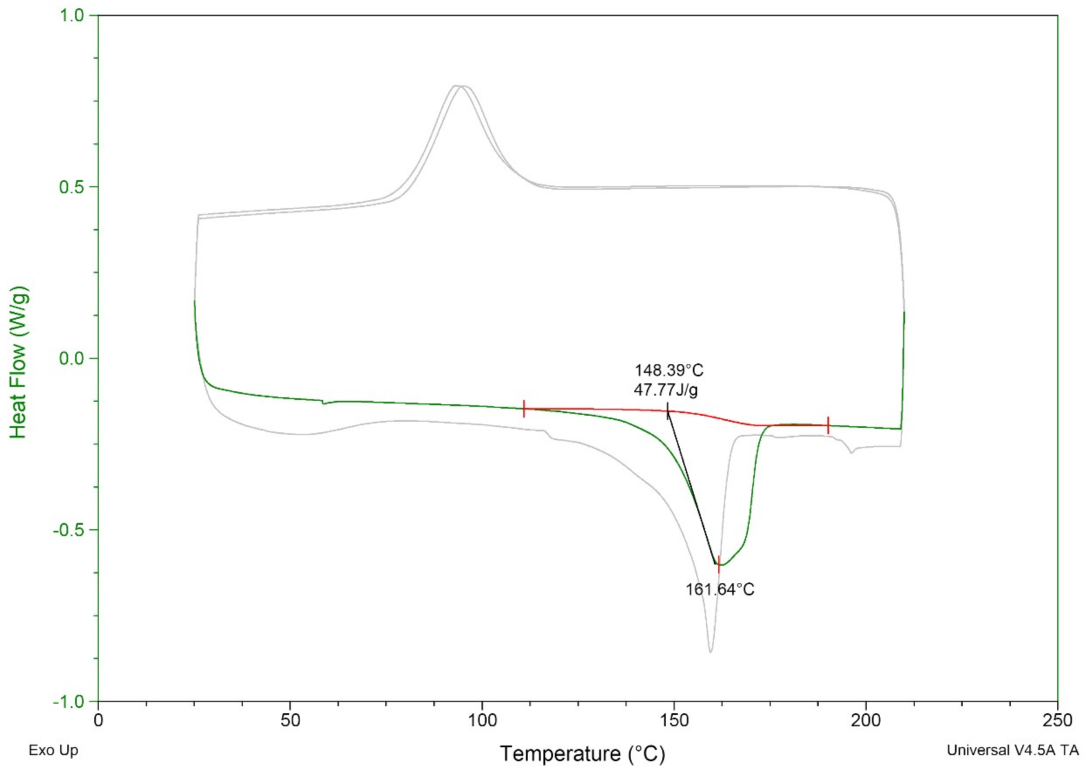

Figure SI45 DSC analysis of PLLA derived from Zn(**5**)$_2$ (180 °C, 4 minutes) at a ratio of 10000:1:30 ([LA]:[I]:[BnOH]) solvent free. Second heating cycle is highlighted and was used for data collection.

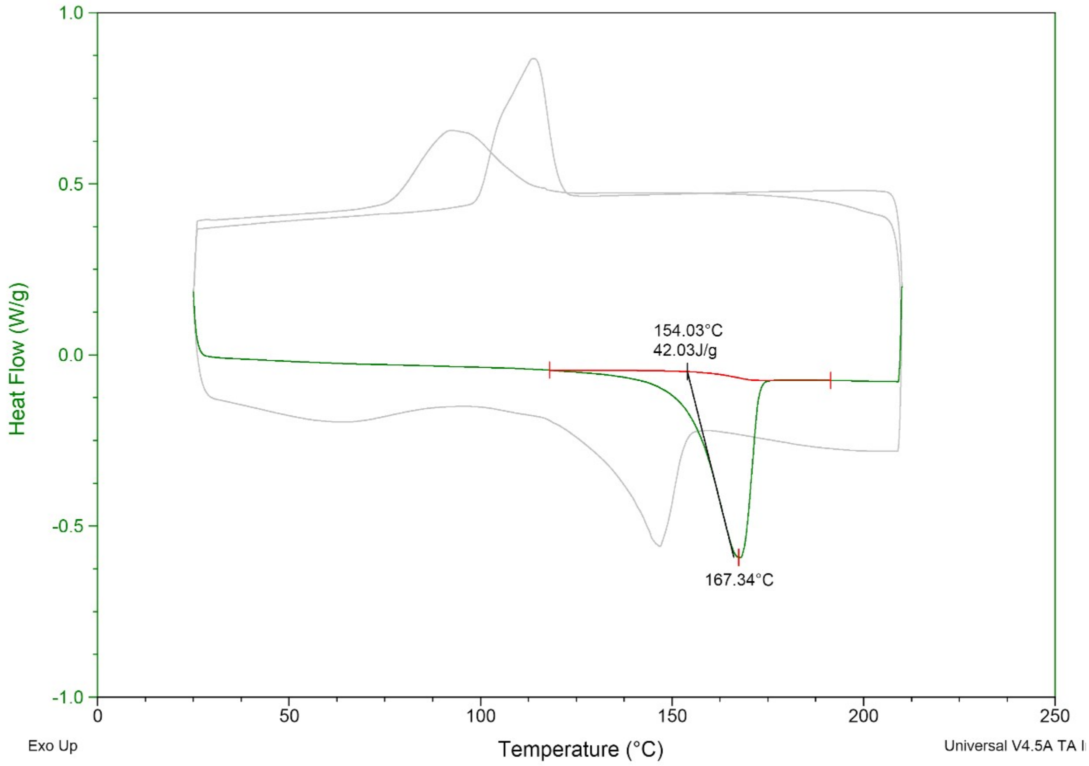

Figure SI 46 DSC analysis of PLLA derived from Zn(**7**)$_2$ (180 °C, 6 minutes) at a ratio of 10000:1:30 ([LA]:[I]:[BnOH]) solvent free. Second heating cycle is highlighted and was used for data collection.

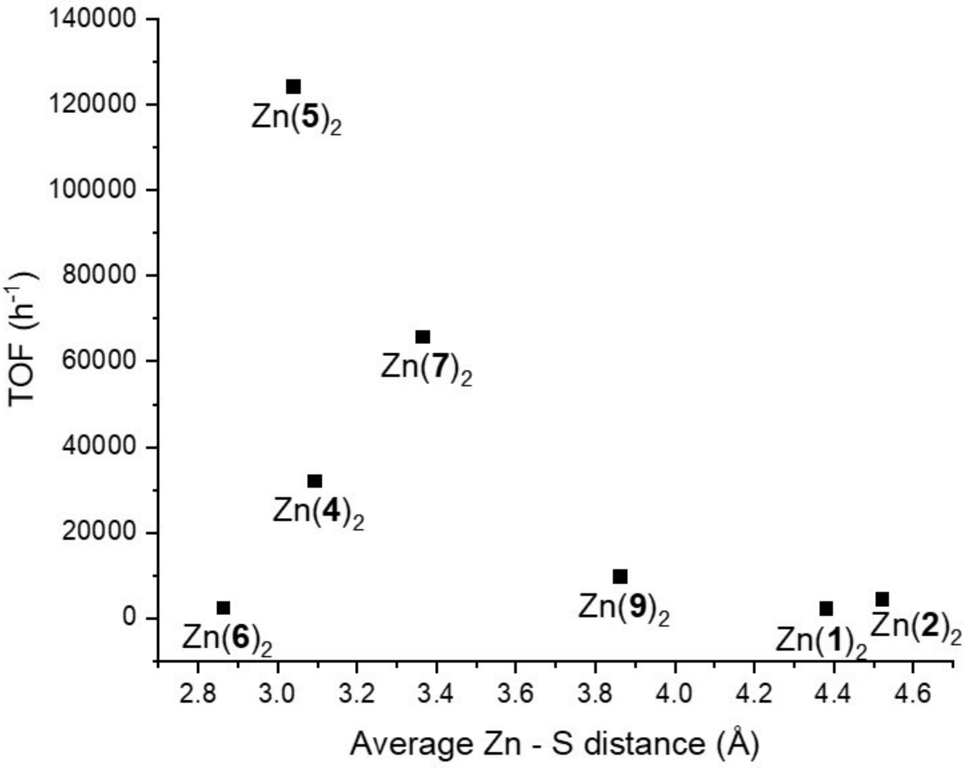

Figure SI47 Average Zn – S bond distances for selected complexes plotted against TOF at ratio of 3000:1:10 ([LA]:[I]:[BnOH]) showing a volcano-type relationship.

Stoichiometric NMR study

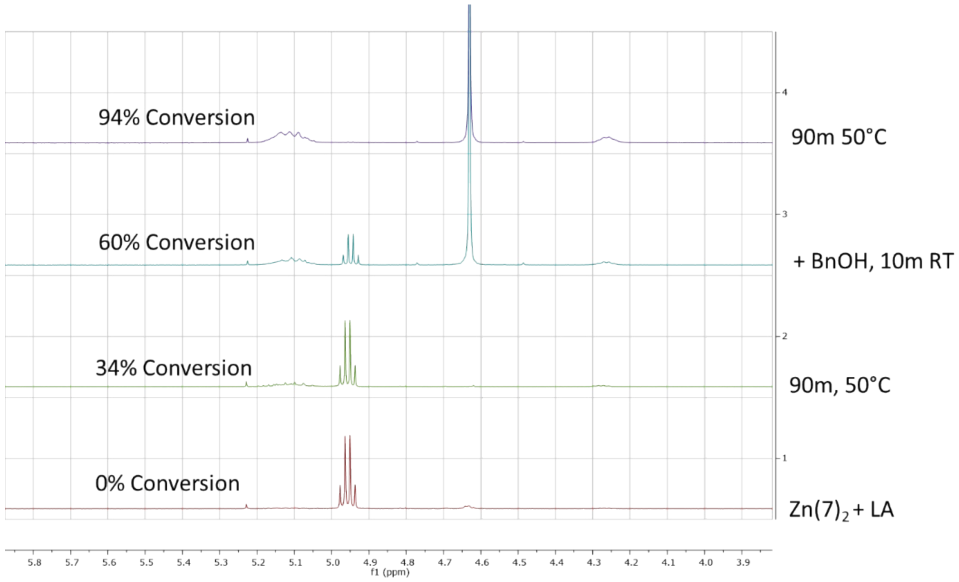

Figure SI48 ^{1}H NMR spectra ($CDCl_3$, 500MHz) of the methine region for stoichiometric reaction between Zn(**7**)$_2$, *rac*-LA and BnOH.

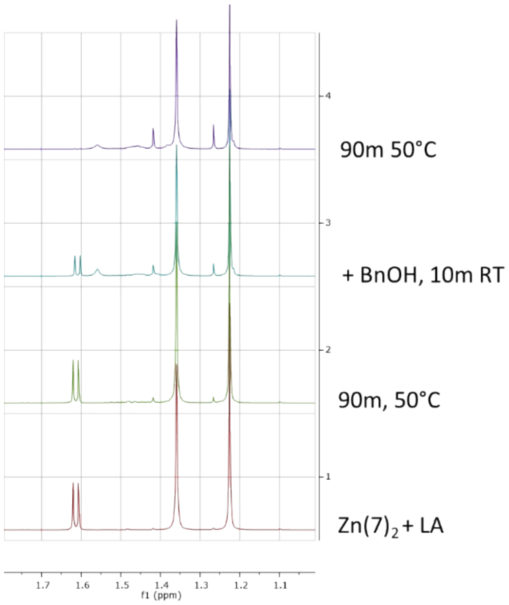

Figure SI49 ^{1}H NMR spectra ($CDCl_3$, 500MHz) of the methine region for stoichiometric reaction between Zn(**7**)$_2$, *rac*-LA and BnOH.

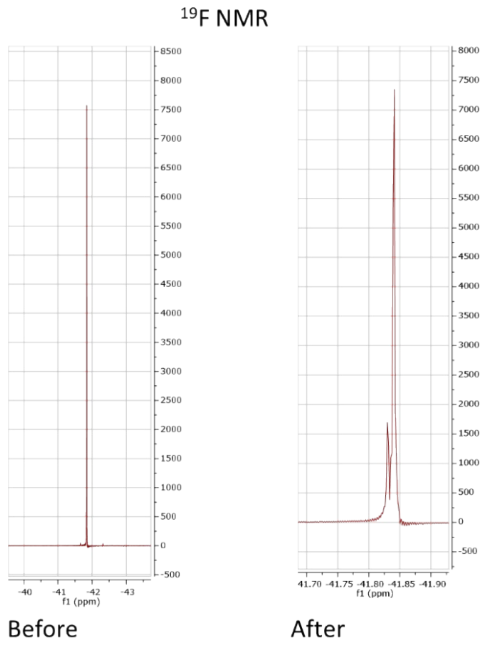

Figure SI50 ^{19}F NMR spectra ($CDCl_3$, 500MHz) of the methine region before and after stoichiometric reaction between Zn(**7**)$_2$, *rac*-LA and BnOH.

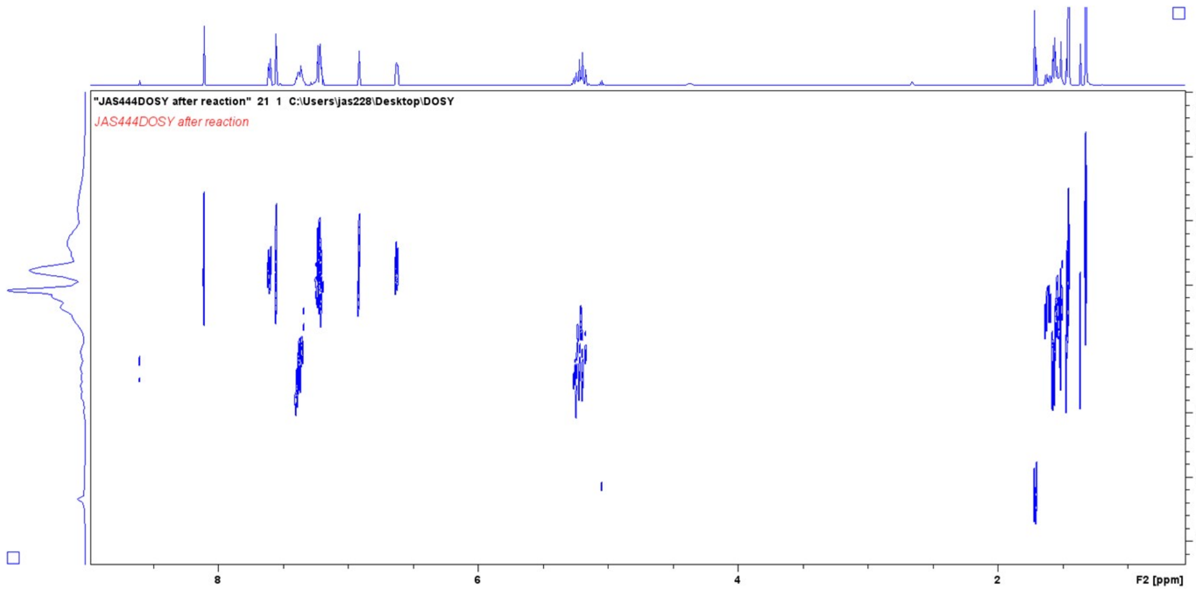

Figure SI51 DOSY NMR spectrum ($CDCl_3$, 500 MHz) after stoichiometric reaction between Zn(**7**)$_2$, *rac*-LA and BnOH.

Raman kinetics

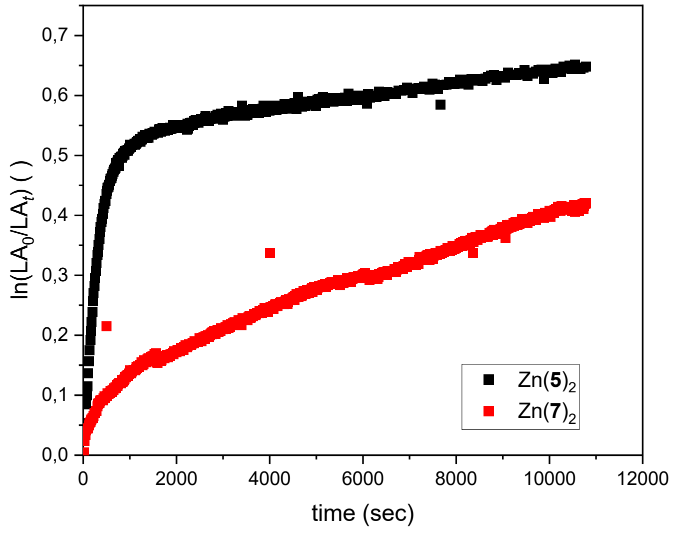

Figure SI52 Kinetic analysis for Zn(**5**,**7**)$_2$. [LA]:[Zn]:[4-MeBnOH] = 3000:1:10, T = 150 °C, 260 rpm, t = 180 min**.** Zn(**5**)$_2$: conversion = 48%, $M_{n\ gpc}$ = 17900 gmol^{-1}; $M_{n\ theo.}$ = 20700; Đ = 1.06. Zn(**7**)$_2$: conversion = 32%.

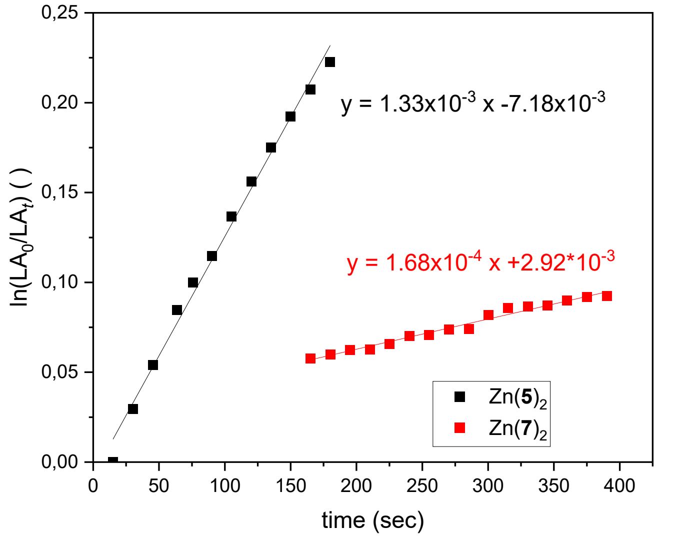

Figure SI53 Kinetic analysis for Zn(**7**,**5**)$_2$. [LA]:[Zn]:[4-MeBnOH] = 3000:1:10. Initial rate.

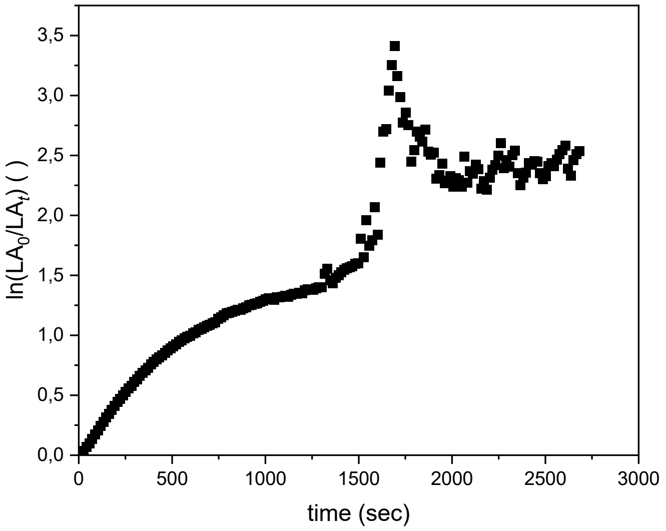

Figure SI54 Kinetic analysis for Zn(7)$_{2.}$ [LA] :[Zn]:[BnOH] = 3000:1:10, T = 150 °C, 260 rpm, t = 45 min, conversion = 75%, $M_{n\ GPC}$ = 31200 gmol^{-1} ; $M_{n\ theo.}$ = 32400 gmol^{-1}; Đ = 1.22.

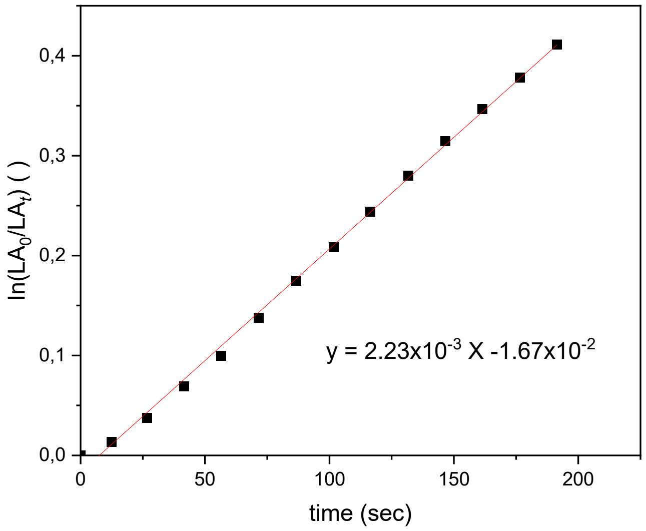

Figure SI55 Kinetic analysis for Zn(**7**)$_{2.}$ [LA] :[Zn]:[BnOH] = 3000:1:10. Initial rate.

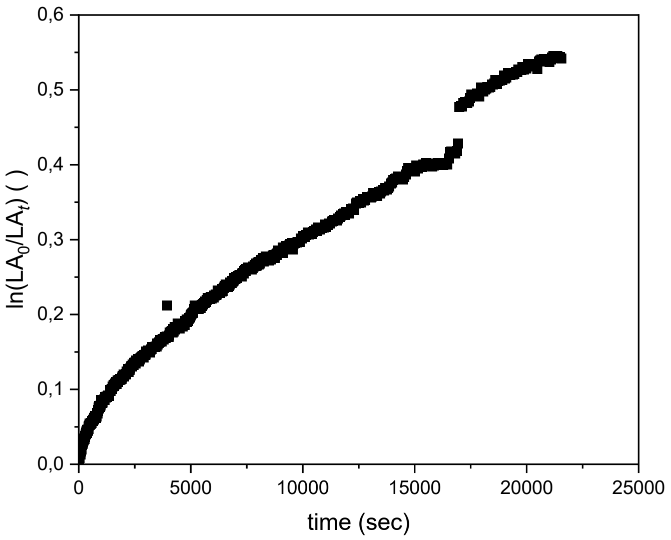

Figure SI56 Kinetic analysis for Zn(**7**)$_2$. [LA] :[Zn]:[BnOH] = 10000:1:100. *T* = 150 °C, 260 rpm, *t* = 45 min, conversion = 34%, $M_{n\ gpc}$ = 2900 gmol^{-1}; $M_{n\ theo.}$ = 4800 gmol^{-1}; Đ = 1.20.

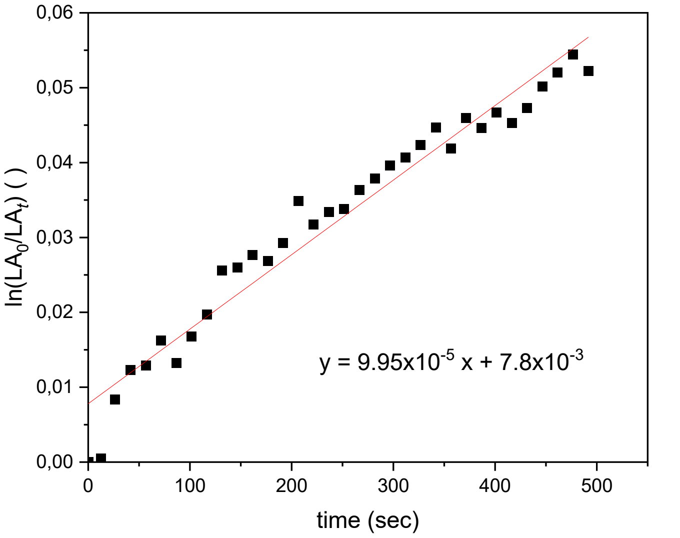

Figure SI57 Kinetic analysis for Zn(**7**)$_2$. [LA] :[Zn]:[BnOH] = 10000:1:100. Initial rate.

Degradation Characterisation

The following equations were used to calculate internal methine conversion (X_{Int}), Me-La selectivity ($S_{Me\text{-}La}$) and Me-La yield ($Y_{Me\text{-}La}$).

$$X_{int} = 1 - \frac{[int]}{[int]_0}$$

$$S_{Me-LA} = \frac{[Me-LA]}{[int]_0 - [int]}$$

$$Y_{Me-LA} = X_{int} S_{Me-LA}$$

[Me-LA] = 1/(1+2.21+1.05) = 23.5%

[Int] = (2.21-1.05)/(1+2.21+1.05) = 27.2%

[CE] = 1.05/(1+2.21+1.05) = 24.6%

[CE] = 1.05/(1+2.21+1.05) = 24.6%

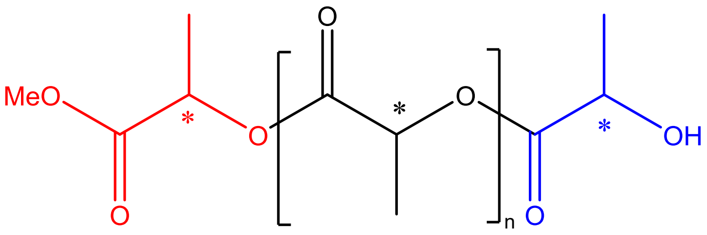

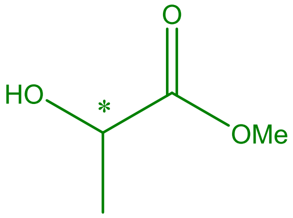

**

* Internal methine

** Chain end methine

* Methyl lactate methine

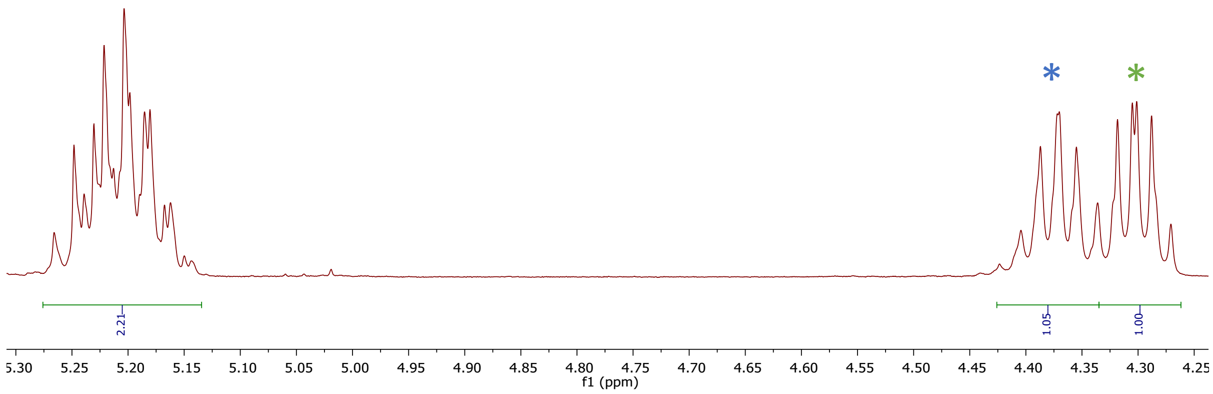

Figure SI58 ^{1}H NMR ($CDCl_3$, 400 MHz) spectrum of PLA Vegware cup degradation into methyl lactate (Me-LA) using Zn(**1**)$_2$ (8 wt% - mol% relative to ester linkages) at 80 °C for 8 h in THF (**Table 6**).

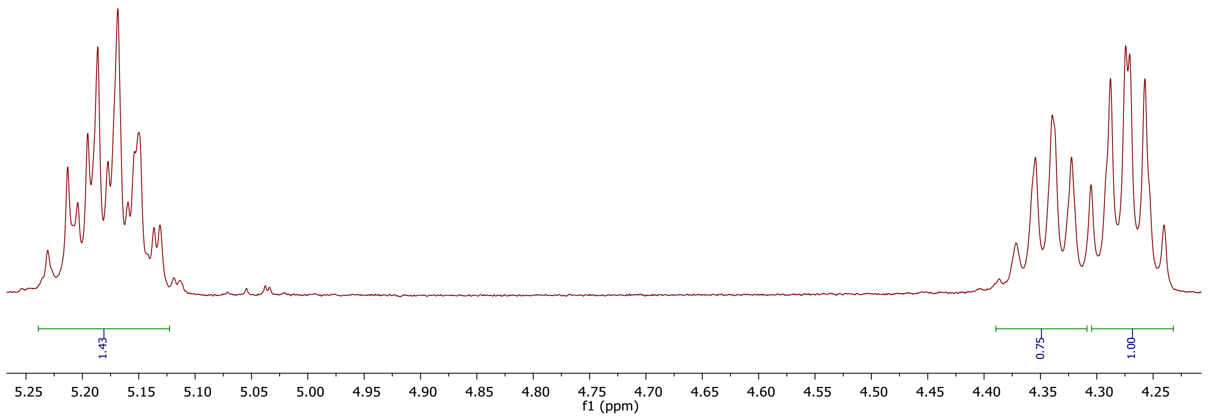

Figure SI 59 ^{1}H NMR ($CDCl_3$, 400 MHz) spectrum of PLA Vegware cup degradation into methyl lactate (Me-LA) using Zn(**6**)$_2$ at 80 °C for 8 h in THF (**Table 6**).

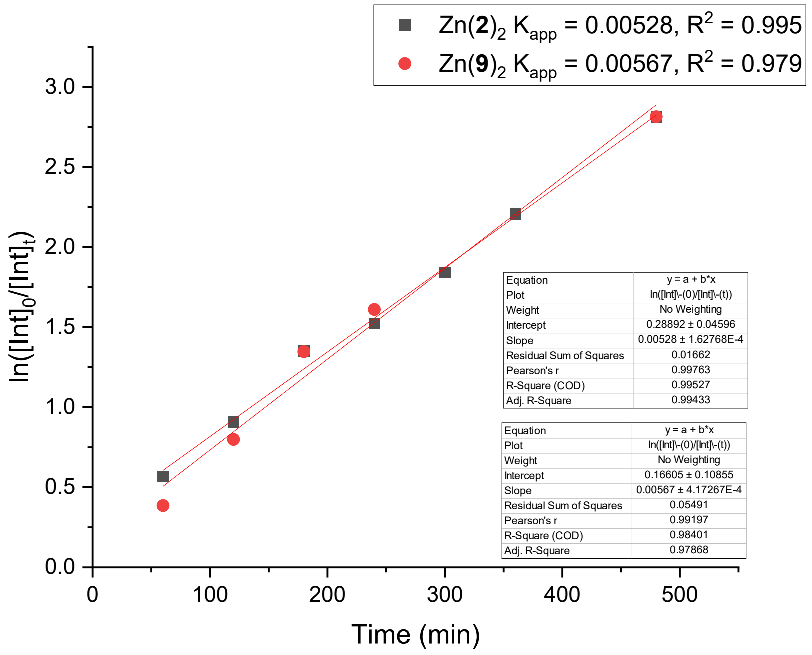

Figure SI60 Pseudo-first-order logarithmic plot for the degradation of PLLA cup using Zn(**2**)$_2$ and Zn(**9**)$_2$ in THF at 80 °C.

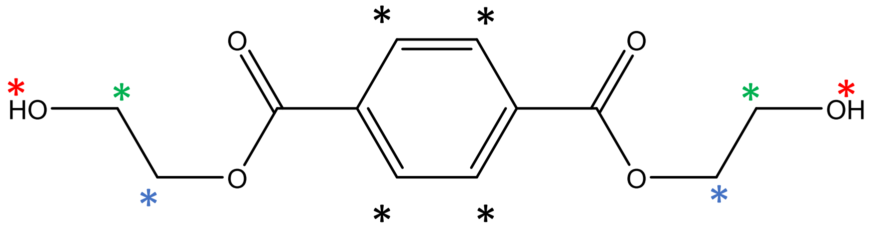

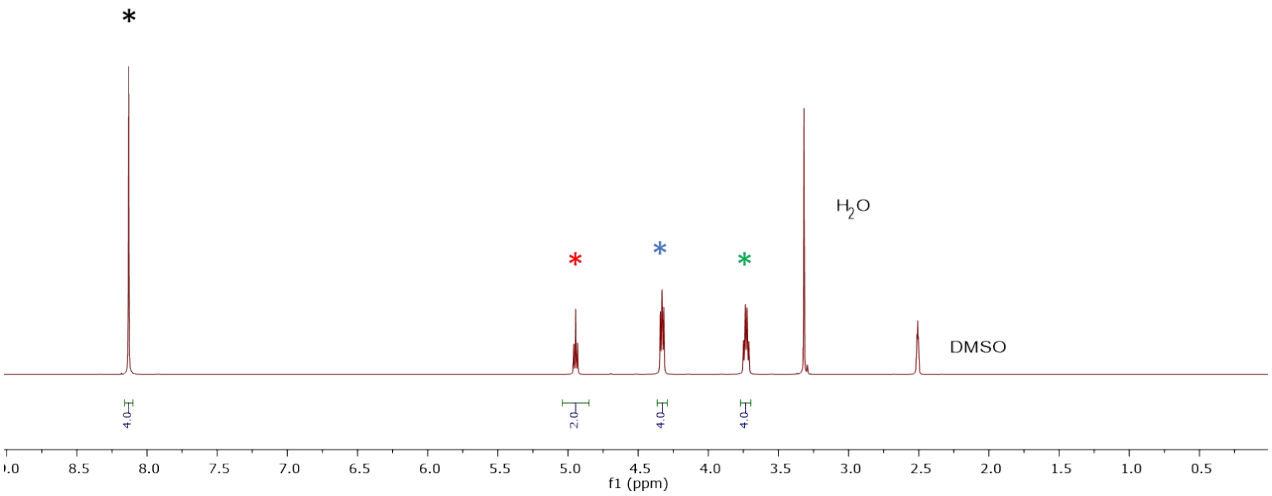

Figure SI61 ^{1}H NMR (D_6-DMSO, 400 MHz) spectrum of recrystallised BHET from PET carbonated drink bottle degradation using Zn(**2**)$_2$.

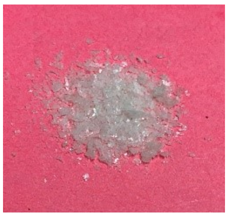

Figure SI62 White crystalline BHET from Zn(**2**)$_2$.

QTAIM analysis

QTAIM[1] analyses were undertaken by using the atomic coordinates from the single-crystal X-ray analysis for a single-point energy calculation using density functional theory. The M06 functional [2] was used along with the def2-TZVP triple-zeta basis set [3] on all centres. The resulting data were used for the QTAIM analysis. Density functional calculations were undertaken with Gaussian 09 [4] and QTAIM analyses with AIMAll version 19.10.12. [5]

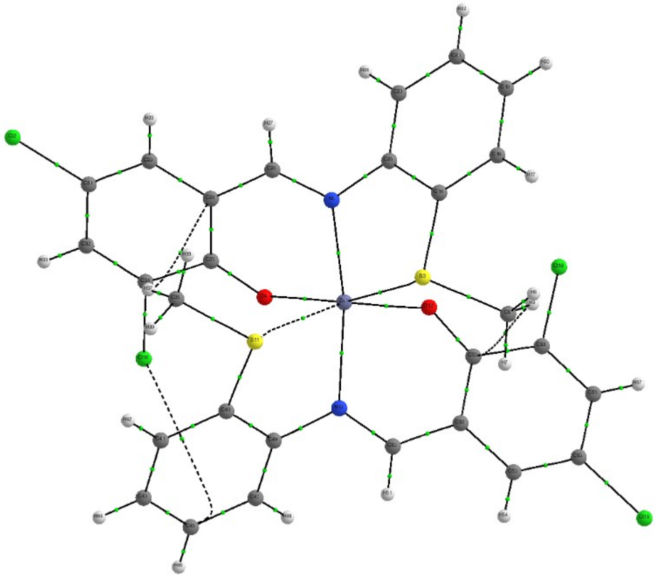

Figure SI63 QTAIM analysis of Zn(**6**)$_2$ showing bond critical points between Zn and S(2).

Compound reference	Zn(**1**)$_1$	Zn(**2**)$_2$	Zn(**4**)$_2$	Zn(**5**)$_2$	Zn(**6**)$_2$	Zn(**7**)$_2$	Zn(**9**)$_2$
Chemical formula	$C_{36}H_{56}N_2O_2S_2$ Zn	$C_{20}H_{24}N_2O_2S$ $_2$Zn	$C_{44}H_{56}N_2O_2S$ $_2$Zn	$C_{91}H_{80}N_6O_6S_6$ Zn_3	$C_{42}H_{36}Cl_4N_2O_2S_2$ Zn	$C_{47}H_{57}F_6N_2O_2S_2$ Zn	$C_{63}H_{36}Cl_8F_{12}$ $N_4O_4S_4Zn_2$
Formula Mass	678.31	453.90	774.39	1742.08	872.02	925.43	1683.54
Crystal system	Monoclinic	Monoclinic	Monoclinic	Orthorhombic	Monoclinic	Monoclinic	Triclinic
a/Å	15.4847(12)	12.8046(4)	18.2174(8)	15.82481(12)	13.8442(5)	15.2189(5)	12.6757(2)
b/Å	18.6395(6)	4.77740(10)	9.9165(2)	28.2281(2)	13.0020(4)	18.0387(7)	13.2778(3)
c/Å	13.4320(3)	16.6707(5)	23.3919(5)	18.02999(15)	22.5589(7)	18.3723(8)	21.3664(4)
α/°	90	90	90	90	90	90	100.5900(14)
β/°	95.841(3)	94.497(3)	106.812(3)	90	97.185(3)	108.104(4)	97.3928(13)
γ/°	90	90	90	90	90	90	103.1820(16)
Unit cell volume/Å^3	3856.7(3)	1016.65(5)	4045.2(2)	8054.07(11)	4028.8(2)	4794.0(3)	3386.58(12)
Temperature/K	150(2)	100(2)	150(2)	150(2)	150(2)	150(2)	150(2)
Space group	*P*2$_1$/*c*	*P*2/*n*	*C*2/*c*	*Pccn*	*P*2$_1$/*c*	*P*2$_1$/*c*	*P***Error!**
No. of formula units per unit cell, *Z*	4	2	4	4	4	4	2
Radiation type	Cu Kα	Cu Kα	Cu Kα	Cu Kα	Mo Kα	Mo Kα	Cu Kα
Absorption coefficient, *μ*/mm^{-1}	2.122	3.737	2.094	2.969	1.017	0.660	5.667
No. of reflections measured	25585	5704	12223	53187	58997	37796	27407
No. of independent reflections	7324	2032	3570	7789	8262	9460	13330
R_{int}	0.0624	0.0466	0.0325	0.0388	0.0610	0.0546	0.0223
Final R_1 values ($I > 2\sigma(I)$)	0.0603	0.0456	0.0292	0.0405	0.0356	0.0468	0.0298
Final $wR(F^2)$ values ($I > 2\sigma(I)$)	0.1663	0.1317	0.0708	0.1066	0.0684	0.0897	0.0775
Final R_1 values (all data)	0.0710	0.0516	0.0330	0.0445	0.0557	0.0753	0.0318
Final $wR(F^2)$ values (all data)	0.1751	0.1361	0.0731	0.1112	0.0750	0.1002	0.0792
Goodness of fit on F^2	1.048	1.103	1.068	1.033	1.015	1.015	1.020

Crystallographic data

Table SI3 X-ray crystallographic parameters.

References

[1] P. S. V. Kumar, V. Raghavendra and V. Subramanian, *Journal of Chemical Sciences*, **2016**, *128*, 1527–1536.

[2] Y. Zhao and D. G. Truhlar, Theor. *Chem. Acc.*, **2008**, *120*, 215–241.

[3] F. Weigend and R. Ahlrichs, *Phys. Chem. Chem. Phys.*, **2005**, *7*, 3297.

[4] M. J. Frisch, G. W. Trucks, H. B. Schlegel, G. E. Scuseria, M. A. Robb, J. R. Cheeseman, G. Scalmani, V. Barone, B. Mennucci, G. A. Petersson, H. Nakatsuji, M. Caricato, X. Li, H. P. Hratchian, A. F. Izmaylov, J. Bloino, G. Zheng, J. L. Sonnenberg, M. Hada, M. Ehara, K. Toyota, R. Fukuda, J. Hasegawa, M. Ishida, T. Nakajima, Y. Honda, O. Kitao, H. Nakai, T. Vreven, J. A. Montgomery Jr, J. E. Peralta, F. Ogliaro, M. Bearpark, J. J. Heyd, E. Brothers, K. N. Kudin, V. N. Staroverov, T. Keith, R. Kobayashi, J. Normand, K. Raghavachari, A. Rendell, J. C. Burant, S. S. Iyengar, J. Tomasi, M. Cossi, N. Rega, J. M. Millam, M. Klene, J. E. Knox, J. B. Cross, V. Bakken, C. Adamo, J. Jaramillo, R. Gomperts, R. E. Stratmann, O. Yazyev, A. J. Austin, R. Cammi, C. Pomelli, J. W. Ochterski, R. L. Martin, K. Morokuma, V. G. Zakrzewski, G. A. Voth, P. Salvador, J. J. Dannenberg, S. Dapprich, A. D. Daniels, O. Farkas, J. B. Foresman, J. V. Ortiz, J. Cioslowski and D. J. Fox, *Gaussian 09, Revision D.01*, Gaussian Inc., Wallingford CT, **2010**.
[5] T. A. Keith, AIMAll, TK Gristmill Software, Overland Park KS, USA, **2017**.
